# Synthesis, Characterization, and Evaluation of the Antifungal Properties of 3-Indolyl-3-Hydroxy Oxindole Derivatives Against Plant Pathogenic Fungi

**DOI:** 10.3390/molecules30051079

**Published:** 2025-02-26

**Authors:** Zhiqiang Bai, Kunrong Dang, Jinrui Tang, Rongjing Yang, Liming Fan, Qiu Li, Yue Yang, Min Ye, Fawu Su

**Affiliations:** 1 State Key Laboratory for Conservation and Utilization of Bio-Resources in Yunnan, College of Plant Protection, Yunnan Agricultural University, Kunming 650201, China; baizhiqiang10@mails.ucas.ac.cn (Z.B.); dang_kr@163.com (K.D.); tjinrui2020@163.com (J.T.); yronjing@163.com (R.Y.); fanliming1976@163.com (L.F.); qiuli0199@163.com (Q.L.); yangyueaq@sina.com (Y.Y.); 2 College of Science, Yunnan Agricultural University, Kunming 650201, China

**Keywords:** 3-indolyl-3-hydroxy oxindoles, phytopathogenic fungi, antifungal activity, structure–activity relationship

## Abstract

To discover novel fungicides with good inhibitory effects on plant fungal diseases, twenty-five 3-indolyl-3-hydroxy oxindole derivatives (3a–3y) were synthesized. These newly derivatives were characterized by NMR and HRMS. Their antifungal activities against five plant pathogenic fungi were assessed in vitro. Most of the compounds exhibited moderate to excellent antifungal activities against the five pathogenic fungi. Notably, compounds **3t**, **3u**, **3v**, and **3w** displayed remarkable and broad-spectrum antifungal activities comparable to or superior to those of the fungicides carvacrol (CA) and phenazine-1-carboxylic acid (PCA). Among them, compound **3u** displayed the most excellent antifungal activity against *Rhizoctonia solani* Kühn (*R. solani*), with an EC_50_ of 3.44 mg/L, which was superior to CA (7.38 mg/L) and PCA (11.62 mg/L). Preliminary structure–activity relationship (SAR) results indicated that the introduction of I, Cl, or Br substituents at position 5 of the 3-hydroxy-2-oxindole and indole rings is crucial for compounds to exhibit good antifungal activity. The in vivo antifungal activity assay showed that compound 3u has good curative effects against *R. solani*. The current results suggest that these compounds are capable of serving as promising lead compounds.

## 1. Introduction

Plant pathogenic fungi pose a severe threat to global agriculture and food security, causing devastating yield losses and substantial economic damage through diseases such as rice sheath blight (caused by *Rhizoctonia solani*), rice blast (*Pyricularia oryzae*/*Magnaporthe oryzae*), gray mold (*Botrytis cinerea*), southern corn leaf blight (*Bipolaris maydis*), and anthracnose (*Colletotrichum gloeosporioides*) [1,2]. These pathogens employ diverse infection strategies: *R. solani*, a soil-borne fungus, persists in soil for extended periods and infects multiple plant tissues, leading to widespread destruction of cereals, legumes, and forage crops [3,4]. *P. oryzae* targets all aerial parts of rice plants throughout their growth cycle [5], while *B. maydis* severely impairs photosynthetic efficiency by damaging functional leaves, with yield losses exceeding 58% in susceptible corn varieties [6,7]. Notably, *C. gloeosporioides* and *B. cinerea* not only reduce postharvest quality but also accelerate the evolution of fungicide resistance through their rapid adaptive mechanisms [8,9,10]. Although chemical fungicides remain the primary control strategy, their prolonged application has exacerbated critical issues such as pesticide resistance, residue accumulation, and ecological degradation [11,12,13]. Consequently, there is an urgent demand for eco-friendly alternatives with novel modes of action to achieve sustainable crop protection and mitigate reliance on conventional agrochemicals [14].

**Figure 1:**
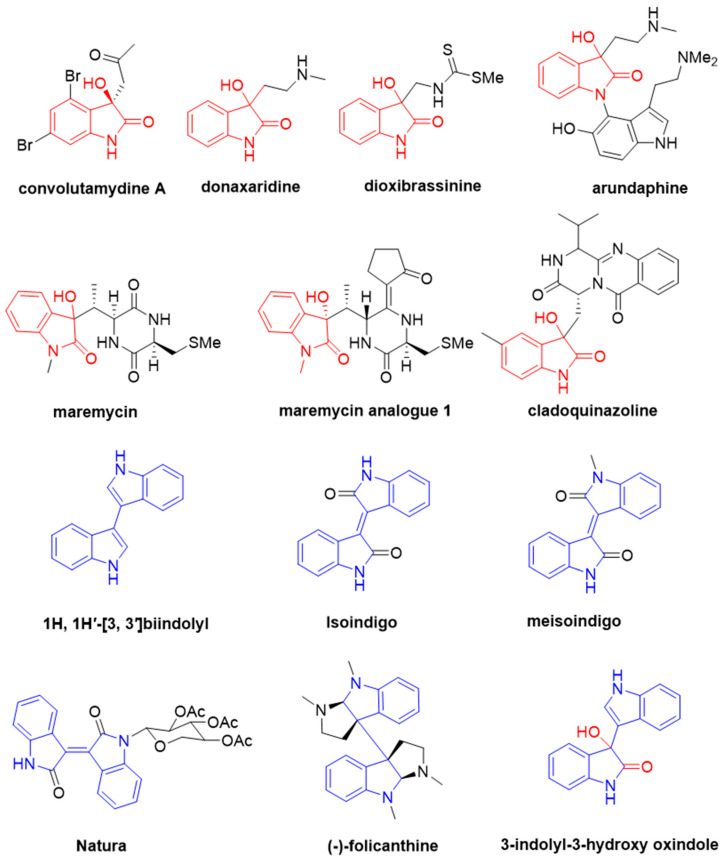
Representative natural 3-hydroxy-2-oxindole and bisindole alkaloids.

Natural products are instrumental in the development of new pesticides, offering numerous models and templates for their design [15]. The 3-hydroxy-2-oxindole structural unit has been identified in various bioactive natural products and molecules (Figure 1), including convolutamydine A, donaxaridine, dioxibrassinine, arundaphine, maremycin, and cladoquinazoline [16,17,18,19,20]. These compounds exhibit anti-tumor, anti-cancer, antioxidant, anti-inflammatory, and proteasome inhibition activities [16,17,20]. Although research on the antifungal activity of 3-hydroxy-2-oxoindole compounds is relatively scarce, there are still some literature reports on the inhibitory effects of these compounds on fungi. For example, the maremycin analogue isolated from the endophytic actinobacterium associated with infected soybeans and obtained from the *Streptomyces capitiformicae* (KX777629) strain DDPA2-14 exhibits high activity against *Sclerotinia sclerotiorum*, with an EC_50_ value of 3.70 mg/L [21].

Bisindole alkaloids, composed of two monomeric indole alkaloid units and widely distributed in nature primarily as metabolites produced by terrestrial and marine organisms (Figure 1) [22,23], exhibit a wide array of biological and pharmacological activities, including antibacterial [24,25] and antifungal properties [26]. Importantly, bisindole alkaloids tend to exhibit higher biological activity compared to their individual monomers [27]. The 3,3′-bisindole motif is a crucial structural component in bisindole alkaloids, a class of natural products known for their complex molecular structures and promising biological activities [28,29]. This class of natural products encompasses 1H, 1H′-[3, 3′]biindolyl, isoindigo, and hexahydropyrroloindole, among others (Figure 1). 1H, 1H′-[3, 3′]biindolyl, a natural product from the terrestrial fungus *Gliocladium catenulatum*, has specific antibacterial activity against the honey bee enthomopathogenic bacterium *P. larvae*. As a compound structurally isomeric with the well-known indigo, isoindigo not only enjoys widespread applications in the pharmaceutical field but also serves as a critical component in various functional materials [30,31]. Its derivatives, such as meisoindigo, have been applied in the treatment of chronic myeloid leukemia in China [32]. Natura is an efficient inhibitor of cell cycle-dependent kinases (CDKs) [33]. The hexahydropyrroloindole-type compound (-)-folicanthine was isolated from the active methanol extract of the seeds of *Chimonanthus praecox* Link and exhibits significant inhibitory activity against five plant pathogenic fungi: *Exserohilum turcicum*, *Bipolaris maydis*, *Alternaria solani*, *Sclerotinia sclerotiorum*, and *Fusarium oxysporum* [34].

3-Indolyl-3-hydroxy oxindoles, which consist of a bisindole and a 3-hydroxy-2-oxindole unit, are significant substrates for the investigation of biological activities [35] and serve as valuable synthetic intermediates for drug candidates and alkaloids [36]. These compounds have demonstrated notable anti-proliferative effects against various cancer cell lines, including leukemia (U937, THP-1), lung (A549), and breast cancer (MCF7) cells [37]. Polymethylene-linked 3-indolyl-3-hydroxy-2-oxindole dimers exhibit selectivity as butyrylcholinesterase (BChE) inhibitors [38]. Research into other biological activities is currently less reported. Recently, we reported that compounds bearing a bisindole structure, specifically bis(indolyl)-hydrazide-hydrazone derivatives, demonstrated potent antifungal activity against various plant fungal diseases [39]. In this study, we fused the 3-hydroxy oxindole and bisindole structures to synthesize 25 3-indolyl-3-hydroxy oxindoles and assessed their antifungal properties against five pathogenic fungi: *Rhizoctonia solani* Kühn (*R. solani*), *Pyricularia oryzae* Cav. (*P. oryzae*), *Colletotrichum gloeosporioides* Penz. (*C. gloeosporioides*), *Botrytis cinerea* Pers.:Fr. (*B. cinerea*), and *Bipolaris maydis* (Nishik.) Shoemaker (*B. maydis*). Among the synthesized compounds, **3u** demonstrated the most potent antifungal activity against *R. solani*, with an efficacy surpassing both carvacrol (CA) and the commercial fungicide phenazine-1-carboxylic acid (PCA). To further explore its practical application potential, the in vivo antifungal performance of compound **3u** was assessed using a broad bean leaf bioassay, where PCA served as the positive control. Additionally, we analyzed the structure–activity relationship of these compounds.

## 2. Results

### 2.1. Chemistry

**Scheme 1:**
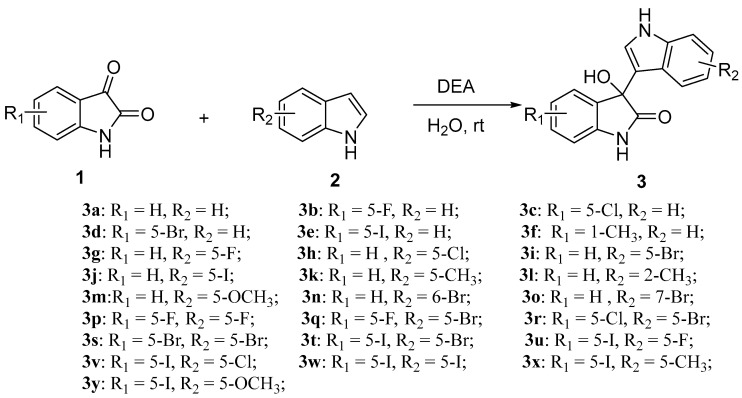
Synthesis route for 3-indolyl-3-hydroxy oxindoles.

As shown in Scheme 1, the general synthetic route for the preparation of 3-indolyl-3-hydroxy oxindole derivatives (**3a**–**3y**) is consistent with the method reported by Prathima et al. [38]. This method uses water as the solvent and diethanolamine as the catalyst to synthesize R-configured 3-indolyl-3-hydroxy oxindole derivatives from isatin (1) and indole (2). A total of twenty-five synthesized derivatives (**3a**–**3y**) were prepared, with yields ranging from 28% to 90%.

### 2.2. In Vitro Antifungal Activity

The inhibitory effects of 3-indolyl-3-hydroxy oxindole derivatives on five plant pathogenic fungi are presented in Table 1. The antifungal activities of the target compounds were assessed in vitro against the mycelium growth of the phytopathogens *R. solani*, *P. oryzae*, *C. gloeosporioides*, *B. cinerea*, and *B. maydis* at concentrations of 50 mg/L, using the commercialized fungicides carvacrol (CA) and phenazine-1-carboxylic acid (shenqinmycin, PCA) as positive controls. The results showed that most of the synthesized compounds exhibited moderate antifungal activity against the five tested fungi. According to the data presented in Table 1, compound **3u** showed favorable antifungal activities against *R. solani*, with inhibition rates of 100% at 50 mg/L, which was superior to that of CA (91.56%) and PCA (81.07%). The inhibitory activity of compounds **3t** (82.48%) and **3w** (87.37%) is comparable to that of the positive control. For *P. oryzae*, compounds **3t**, **3u**, **3v**, and **3w** demonstrated good antifungal activities (inhibition rate > 75%), which were better than that of carvacrol (74.94%) and PCA (53.64%) at 50 mg/L. The compounds **3t** and **3w** exhibited moderate inhibitory activity against *C. gloeosporioides* at a concentration of 50 mg/L, with inhibition rates of 61.62% and 66.67%, respectively. This performance is comparable to that of CA (62.65%) but lower than that of PCA (77.96%). Three compounds exhibited inhibition rates against *B. cinerea* that exceeded 80% at a concentration of 50 mg/L. Among them, compound **3u** achieved an inhibition rate of 91.05%, which was superior to that of CA (84.38%) and PCA (81.86%). Compounds **3h** and **3i** showed inhibition rates of 80.82% and 80.42%, respectively, which were comparable to those of CA and PCA. Regarding *B. maydis*, the four compounds **3t**, **3u**, **3v**, and **3w** exhibited significant antifungal activity, with inhibition rates of 86.56%, 81.13%, 92.22%, and 89.85%, respectively, at a concentration of 50 mg/L, which is superior to CA’s 64.36% and comparable to PCA’s 98.01%. In general, compounds **3t**, **3u**, **3v**, and **3w** exhibited noteworthy broad-spectrum antifungal activities against the five fungi.

To more thoroughly study the inhibitory performance of the target compounds on plant pathogenic fungi, the EC_50_ values of the compounds with favorable inhibition rates at a concentration of 50 mg/L were tested. As shown in Table 2, the title compounds showed good antifungal activities against *R. solani*, *P. oryzae*, *B. cinerea*, and *B. maydis*. For *R. solani*, compound **3u** demonstrated the highest inhibitory activity, with an EC_50_ of 3.44 mg/L, which was superior to CA (7.38 mg/L) and PCA (11.62 mg/L). Compounds **3v** and **3w** exhibited EC_50_ values of 14.72 and 15.69 mg/L for *P. oryzae*, respectively, which were superior to CA and PCA (25.30 and 64.53 mg/L, respectively). Compounds **3h** and **3u** showed EC_50_ values of 12.05 and 11.89 mg/L for *B. cinerea*, respectively, which were superior to CA and PCA (21.36 and 14.75 mg/L, respectively). Compounds **3t**, **3u**, **3v**, and **3w** demonstrated EC_50_ values of 13.62, 12.76, 13.47, and 10.55 mg/L, respectively, for *B. maydis*, which were all superior to CA (18.58 mg/L) and higher than PCA (3.09 mg/L). It is noteworthy that compounds **3t**, **3u**, **3v**, and **3w** exhibited broad-spectrum antifungal activities that were comparable to or superior to those of the fungicides CA and PCA, thereby qualifying them as priority candidates for further study.

### 2.3. In Vivo Antifungal Activity

As summarized in Table 2, compound **3u** exhibited the most potent antifungal activity against *R. solani*, with an EC_50_ value of 3.44 mg/L, demonstrating superior efficacy to both carvacrol (CA; EC_50_ = 7.38 mg/L) and phenazine-1-carboxylic acid (PCA; EC_50_ = 11.62 mg/L).

**Figure 2:**
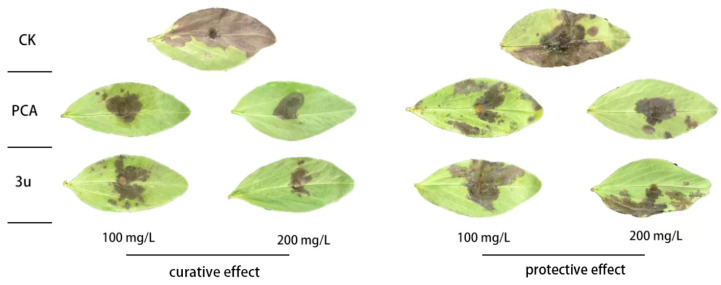
In vivo protective effects of compound **3u** and PCA (phenazine-1-carboxylic acid) against *R. solani*. CK, control.

To further investigate its practical potential, the in vivo antifungal performance of compound **3u** was evaluated using a broad bean leaf bioassay, with the commercial fungicide PCA as the positive control. As illustrated in Figure 2 and Table 3, compound **3u** displayed promising curative effects, achieving control efficacies of 58.41% and 81.93% at concentrations of 100 and 200 mg/L, respectively. Although these values were slightly lower than those of PCA (71.62% and 83.84% at the same concentrations), the high activity at 200 mg/L suggests comparable therapeutic potential under elevated dosage conditions.

In contrast, the protective efficacy of compound **3u** was relatively limited, with control rates of 16.30% and 28.46% at 100 and 200 mg/L, respectively. This performance was significantly inferior to PCA, which showed protective efficacies of 53.43% and 61.18% at corresponding concentrations. The marked disparity between curative and protective effects implies that compound **3u** may function primarily through direct antifungal action rather than systemic induction of plant defense mechanisms.

These findings highlight compound **3u** as a promising candidate for therapeutic intervention against *R. solani*, while further structural optimization may be required to enhance its preventive capabilities.

## 3. Discussion

The target compounds were synthesized following the method reported by Prathima et al. [38], wherein diethanolamine was identified as an efficient catalyst for the aqueous-phase synthesis of 3-indolyl-3-hydroxy oxindole derivatives. While our synthetic yields (ranging from 28% to 90%) were moderately lower than those described in the literature (80–98%) [30], this discrepancy likely arose from losses during purification steps, particularly recrystallization and column chromatography. Given that our primary objective focused on evaluating the biological activity of these compounds, further optimization of synthetic yields was not pursued. Notably, although the method reported by Prathima et al. [38] demonstrated the catalytic superiority of diethanolamine over other amine catalysts in water, the underlying mechanism remains unexplored. We hypothesize that the emulsifying properties of diethanolamine may enhance the solubility of isatin and indole derivatives in aqueous media, thereby facilitating the electrophilic addition. Furthermore, the stereochemical configuration of the asymmetric carbon adjacent to the hydroxyl group was unambiguously assigned as R based on X-ray crystallographic data from analogous compounds reported by Prathima et al. [38].

**Figure 3:**
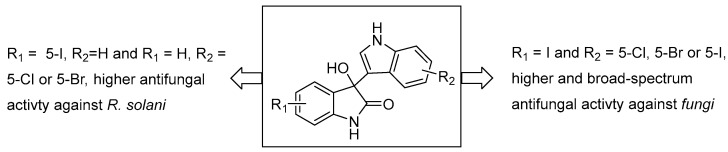
In vitro structure–activity relationship of 3-indolyl-3-hydroxy oxindoles.

By analyzing the results in Table 1, we found that the compounds studied in this work exhibited higher sensitivity to *R. solani*, *P. oryzae*, *B. cinerea*, and *B. maydis* compared to *C. gloeosporioides*. Based on the data listed in Table 1 and Table 2, we conducted an analysis of the in vitro structure–activity relationship of the target compounds. When the ring of the isatin fragment is substituted with Cl, Br, or I, the inhibitory activity of the compound is higher. In comparison, when the ring of the indole fragment is substituted with Cl and Br, the compound exhibits stronger antifungal activity; compounds with F, I, and other substituents typically have lower inhibitory activity (as shown in Figure 3). It was found that compounds with substitution at position 5 are the most active; for example, compound **3i** (5-Br) showed a much higher inhibition rate against *B. cinerea* at 50 mg/L concentration than compounds **3n** (6-Br) and **3o** (7-Br). When both the isatin and indole rings have substituents, compounds composed of an I substituent at position 5 of the isatin and Cl, Br, and I substituents at position 5 of the indole exhibit excellent broad-spectrum antifungal activity. Compounds with other substituents generally have lower antifungal activity. These research results indicate that iodine substitution plays a crucial role in the antifungal activity of the compounds.

The structure–activity relationships (SARs) of bis-indole compounds have been extensively studied in multiple reports [40,41,42,43,44,45,46,47], and while some discrepancies exist among certain findings [40], the overall trend indicates that halogen-substituted bis-indole compounds generally exhibit enhanced antibacterial activity [43,44,45,46,47]. Our study demonstrates that indole-ring halogen-substituted compounds possess superior antifungal activity, consistent with the previous literature [43,44,45,46,47]. For example, Guo et al. observed that brominated nortopsentin analogues exhibited higher antifungal efficacy against *Alternaria solani* [45], while Rehberg et al. reported that 5-chlorinated bis-indole derivatives showed the strongest antibacterial activity against methicillin-resistant *Staphylococcus aureus* [46]. Similarly, Yan et al. found that F-, Cl-, and Br-substituted compounds displayed stronger antibacterial effects against two Gram-positive strains, *Staphylococcus aureus* and *Bacillus subtilis*. However, the influence of specific substitution positions on biological activity remains unclear; Huang et al. noted that 4-substituted bis-indole compounds exhibited stronger antifungal activity [39], whereas Guo et al. focused on substitutions at the 5th and 6th positions [45], and Rehberg et al. investigated substitution at the 5th position [46]. In contrast, Yan et al. observed potent antibacterial activity for halogenated compounds at positions 4, 5, 6, and 7 [47], while our results indicated that 5-substituted compounds exhibit better antifungal activity. Additionally, dihalogenated compounds often display enhanced biological activity [47], aligning with our findings. Notably, most previously reported bis-indole derivatives lacked iodine (I)-substituted analogues, whereas our study revealed that I-substituted compounds exhibit superior biological activity. These findings emphasize that both the specific position of substitution and the nature of the substituent significantly impact compound bioactivity, providing a promising avenue for the development of more effective antifungal agents.

## 4. Materials and Methods

### 4.1. General Information

All indole compounds were purchased from Shanghai Energy Chemical Technology Co., Ltd. (Shanghai, China). and Shanghai Adamas Beta Chemical Reagent Co., Ltd. (Shanghai, China). Shenqinmycin (phenazine-1-carboxylic acid, 98%) was purchased from Meryer (Shanghai) Biochemical Technology Co., Ltd. (Shanghai, China). Other reagents and solvents were of reagent grade or purified according to standard methods before use. Analytical thin-layer chromatography (TLC) was performed with silica gel plates using silica gel 60 GF254 (Qingdao Haiyang Chemical Co., Ltd., Qingdao, China). The ^1^H NMR (400 MHz) and ^13^C NMR (101 MHz) were recorded on an Bruker AVANCE NEO 400MHZ FT-NMR spectrometer (Bruker Corporation, Billerica, MA, USA) with DMSO-*d_6_* as the solvent and TMS as the internal standard. ^1^H NMR and ^13^C NMR chemical shifts are reported in ppm (δ), with the solvent (DMSO-*d_6_*) peaks employed as the internal standard (3.33 ppm for ^1^H and 39.52 ppm for ^13^C). Data are reported as follows: chemical shift, multiplicity (s = singlet, brs = broad singlet, d = doublet, t = triplet, q = quartet, m = multiplet, dd = doublet of doublets, dt = doublet of triplets, and td = triplet of doublets), coupling constants (Hz), and integration. High-resolution mass spectra (HRMS) were determined with a Thermo Fisher Scientific Q Exactive Focus (Thermo Fisher Scientific Inc., Waltham, MA, USA).

### 4.2. Synthetic Procedures

The synthesis of 3-indolyl-3-hydroxy oxindole derivatives **3** was according to the method described by Prathima et al. [38]. At room temperature, indole (2 mmol) and diethanolamine (20 mol%) were slowly added to a solution of isatin (2 mmol) in water (8 mL). After the reaction was complete as monitored by TLC, the reaction mixture was extracted with ethyl acetate and washed with a brine solution. The organic layer was dried over anhydrous Na_2_SO_4_ and concentrated under reduced pressure to obtain a crude product, which was subsequently purified through recrystallization from ethanol or isolated via column chromatography to obtain the compound. The chemical structure of the title compounds was characterized and confirmed by ^1^H NMR, ^13^C NMR, and HRMS. The characterization data of compounds **3a**–**3y** are listed as follows:

3-hydroxy-3-(1H-indol-3-yl)indolin-2-one (**3a**). Isolated yield: 68%; orange-yellow solid; ^1^H NMR (400 MHz, DMSO-*d_6_*) δ 10.94 (s, 1H), 10.30 (s, 1H), 7.35–7.27 (m, 2H), 7.22 (m, 2H), 7.04 (d, *J* = 2.6 Hz, 1H), 7.00 (t, *J* = 8.1 Hz, 1H), 6.93 (t, *J* = 7.5, 1H), 6.88 (d, *J* = 7.6 Hz, 1H), 6.84 (t, *J* = 8.1 Hz, 1H), 6.31 (s, 1H). ^13^C NMR (101 MHz, DMSO-*d_6_*) δ 178.43, 141.65, 136.78, 133.42, 129.02, 124.90, 124.74, 123.49, 121.66, 121.03, 120.30, 118.44, 115.42, 111.46, 109.59, 74.89.

5-fluoro-3-hydroxy-3-(1H-indol-3-yl)indolin-2-one (**3b**). Isolated yield: 51%; white solid; ^1^H NMR (400 MHz, DMSO-*d_6_*) δ 10.98 (d, *J* = 2.6 Hz, 1H), 10.33 (s, 1H), 7.38–7.26 (m, 2H), 7.12–6.94 (m, 4H), 6.91–6.78 (m, 2H), 6.45 (s, 1H). ^13^C NMR (101 MHz, DMSO-*d_6_*) δ 178.37, 159.23, 156.87, 137.79 (d), 136.81, 135.18 (d), 124.78, 123.62, 121.16, 120.19, 118.61, 115.41-114.83 (t), 112.26 (d), 111.58, 110.50 (d), 75.21 (d).

5-chloro-3-hydroxy-3-(1H-indol-3-yl)indolin-2-one (**3c**). Isolated yield: 65%; pale-yellow solid; ^1^H NMR (400 MHz, DMSO-*d_6_*) δ 11.00 (d, *J* = 2.7 Hz, 1H), 10.46 (s, 1H), 7.37–7.29 (m, 2H), 7.27 (dd, *J* = 8.3, 2.2 Hz, 1H), 7.17 (d, *J* = 2.2 Hz, 1H), 7.07 (d, *J* = 2.5 Hz, 1H), 7.05–6.96 (m, 1H), 6.94–6.81 (m, 2H), 6.49 (s, 1H). ^13^C NMR (101 MHz, DMSO-*d_6_*) δ 178.05, 140.53, 136.81, 135.47, 128.90, 125.67, 124.70, 124.63, 123.61, 121.20, 120.07, 118.67, 114.68, 111.63, 111.23, 74.99.

5-bromo-3-hydroxy-3-(1H-indol-3-yl)indolin-2-one (**3d**). Isolated yield: 89%; pale-yellow solid; ^1^H NMR (400 MHz, DMSO-*d_6_*) δ 11.00 (s, 1H), 10.47 (s, 1H), 7.40 (dd, *J* = 8.2, 2.1 Hz, 1H), 7.36–7.21 (m, 3H), 7.07 (d, *J* = 2.6 Hz, 1H), 7.01 (t, *J* = 7.7 Hz, 1H), 6.86 (t, *J* = 8.7 Hz, 2H), 6.49 (s, 1H). ^13^C NMR (101 MHz, DMSO-*d_6_*) δ 177.92, 140.94, 136.81, 135.87, 131.75, 127.35, 124.68, 123.60, 121.21, 120.02, 118.69, 114.68, 113.36, 111.78, 111.65, 74.95.

3-hydroxy-3-(1H-indol-3-yl)-5-iodoindolin-2-one (**3e**). Isolated yield: 93%; pale-yellow solid; ^1^H NMR (400 MHz, DMSO-*d_6_*) δ 11.00 (d, *J* = 2.7 Hz, 1H), 10.46 (s, 1H), 7.56 (dd, *J* = 8.1, 1.9 Hz, 1H), 7.42 (d, *J* = 1.9 Hz, 1H), 7.31 (t, *J* = 8.3 Hz, 2H), 7.07 (d, *J* = 2.5 Hz, 1H), 7.01 (t, *J* = 7.6 Hz, 1H), 6.86 (t, *J* = 7.5 Hz, 1H), 6.74 (d, *J* = 8.1 Hz, 1H), 6.46 (s, 1H). ^13^C NMR (101 MHz, DMSO-*d_6_*) δ 177.71, 141.41, 137.55, 136.79, 136.13, 132.87, 124.65, 123.56, 121.19, 119.95, 118.67, 114.75, 112.29, 111.65, 84.47, 74.78.

3-hydroxy-3-(1H-indol-3-yl)-1-methylindolin-2-one (**3f**). Isolated yield: 90%; pale-yellow solid; ^1^H NMR (400 MHz, DMSO-*d_6_*) 10.95 (d, *J* = 2.6 Hz, 1H), 7.27 (m, 4H), 7.05–6.92 (m, 4H), 6.80 (td, *J* = 8.2, 1.0 Hz, 1H), 6.39 (s, 1H), 3.10 (s, 3H). ^13^C NMR (101 MHz, DMSO-*d_6_*) δ 176.66, 143.11, 136.82, 132.76, 129.21, 124.89, 124.34, 123.65, 122.43, 121.14, 120.32, 118.59, 115.16, 111.55, 108.57, 74.68, 26.01.

3-(5-fluoro-1H-indol-3-yl)-3-hydroxyindolin-2-one (**3g**). Isolated yield: 54%; pale-yellow solid; ^1^H NMR (400 MHz, DMSO-*d_6_*) δ 11.05 (d, *J* = 2.7 Hz, 1H), 10.28 (s, 1H), 7.29 (dd, *J* = 8.8, 4.6 Hz, 1H), 7.26–7.18 (m, 2H), 7.09 (dd, *J* = 10.5, 2.6 Hz, 1H), 7.00 (d, *J* = 2.6 Hz, 1H), 6.98–6.91 (m, 1H), 6.87-6.82 (m, 2H), 6.35 (s, 1H). ^13^C NMR (101 MHz, DMSO-*d_6_*) δ 178.31, 157.59, 155.29, 141.66, 133.52, 132.97, 129.21, 125.57, 125.26 (d), 124.81, 121.80, 115.62 (d), 112.53, 112.44, 109.70-109.21 (t), 105.27 (d), 74.70.

3-(5-chloro-1H-indol-3-yl)-3-hydroxyindolin-2-one (**3h**). Isolated yield: 66%; pale-yellow solid; ^1^H NMR (400 MHz, DMSO-*d_6_*) 11.15 (d, *J* = 2.5 Hz, 1H), 10.31 (s, 1H), 7.49 (d, *J* = 2.1 Hz, 1H), 7.32 (d, *J* = 8.6 Hz, 1H), 7.24 (td, *J* = 7.4, 1.3 Hz, 2H), 7.01 (dd, *J* = 8.6, 2.1 Hz, 1H), 6.99–6.92 (m, 2H), 6.90–6.84 (m, 1H), 6.39 (s, 1H). ^13^C NMR (101 MHz, DMSO-*d_6_*) δ 178.25, 141.65, 135.33, 132.90, 129.26, 126.21, 125.29, 124.80, 123.12, 121.84, 121.11, 120.06, 115.33, 113.09, 109.72, 74.67.

3-(5-bromo-1H-indol-3-yl)-3-hydroxyindolin-2-one (**3i**). Isolated yield: 49%; yellow solid; ^1^H NMR (400 MHz, DMSO-*d_6_*) δ 11.15 (d, *J* = 2.7 Hz, 1H), 10.30 (s, 1H), 7.66 (d, *J* = 1.9 Hz, 1H), 7.30–7.18 (m, 3H), 7.11 (dd, *J* = 8.6, 2.0 Hz, 1H), 6.99–6.91 (m, 2H), 6.86 (d, *J* = 7.4 Hz, 1H), 6.38 (s, 1H). ^13^C NMR (101 MHz, DMSO-*d_6_*) δ 178.24, 141.66, 135.56, 132.89, 129.28, 126.95, 125.11, 124.81, 123.64, 123.17, 121.85, 115.27, 113.58, 111.20, 109.72, 74.68. HRMS (ESI): *m*/*z* [M+Na]^+^ calcd for [C_16_H_11_BrN_2_O_2_Na]^+^: 364.9896, found: 364.9882.

3-(5-iodo-1H-indol-3-yl)-3-hydroxyindolin-2-one (**3j**). Isolated yield: 28%; yellow solid; ^1^H NMR (400 MHz, DMSO-*d_6_*) δ 11.13 (s, 1H), 10.31 (s, 1H), 7.89 (d, *J* = 1.7 Hz, 1H), 7.30–7.20 (m, 3H), 7.18 (d, *J* = 8.5 Hz, 1H), 6.97 (td, *J* = 7.5, 1.1 Hz, 1H), 6.91–6.83 (m, 2H), 6.38 (s, 1H). ^13^C NMR (101 MHz, DMSO-*d_6_*) δ 178.21, 141.64, 135.89, 132.92, 129.45, 129.24, 129.03, 127.86, 124.78, 124.58, 121.82, 114.95, 114.05, 109.68, 82.54, 74.70.

3-hydroxy-3-(5-methyl-1H-indol-3-yl)indolin-2-one (**3k**). Isolated yield: 93%; yellow solid; ^1^H NMR (400 MHz, DMSO-*d_6_*) δ 10.80 (d, *J* = 2.7 Hz, 1H), 10.27 (s, 1H), 7.29–7.10 (m, 4H), 6.97–6.73 (m, 4H), 6.26 (s, 1H), 2.23 (s, 3H). ^13^C NMR (101 MHz, DMSO-*d_6_*) δ 178.49, 141.63, 135.19, 133.50, 128.98, 126.64, 125.24, 124.73, 123.50, 122.66, 121.65, 120.14, 114.92, 111.18, 109.56, 74.94, 21.45.

3-hydroxy-3-(2-methyl-1H-indol-3-yl)indolin-2-one (**3l**). Isolated yield: 69%; orange-yellow solid; ^1^H NMR (400 MHz, DMSO-*d_6_*) δ 10.84 (s, 1H), 10.31 (s, 1H), 7.32–7.06 (m, 3H), 6.94–6.86 (m, 4H), 6.70 (t, *J* = 7.4 Hz, 1H), 6.24 (s, 1H), 2.38 (s, 3H). ^13^C NMR (101 MHz, DMSO-*d_6_*) δ 178.66, 141.57, 134.84, 134.13, 133.42, 128.98, 126.61, 124.92, 121.68, 119.78, 119.18, 118.16, 110.23, 109.60, 109.41, 75.86, 13.30.

3-hydroxy-3-(5-methoxy-1H-indol-3-yl)indolin-2-one (**3m**). Isolated yield: 61%; grayish-white solid; **^1^H NMR** (400 MHz, DMSO-*d_6_*) δ 10.78 (d, *J* = 2.7 Hz, 1H), 10.27 (s, 1H), 7.31–7.10 (m, 3H), 6.96 (d, *J* = 2.6 Hz, 1H), 6.93 (td, *J* = 7.5, 1.0 Hz, 1H), 6.89–6.83 (m, 1H), 6.79 (d, *J* = 2.5 Hz, 1H), 6.65 (dd, *J* = 8.8, 2.5 Hz, 1H), 6.28 (s, 1H), 3.57 (s, 3H). ^13^C NMR (101 MHz, DMSO-*d_6_*) δ 178.43, 152.72, 141.69, 133.35, 131.98, 129.04, 125.35, 124.82, 124.21, 121.70, 115.00, 112.02, 110.86, 109.54, 102.64, 74.93, 55.15.

3-(6-bromo-1H-indol-3-yl)-3-hydroxyindolin-2-one (**3n**). Isolated yield: 31%; pale-yellow solid; ^1^H NMR (400 MHz, DMSO-*d_6_*) δ 11.07 (d, *J* = 2.7 Hz, 1H), 10.30 (s, 1H), 7.49 (d, *J* = 1.9 Hz, 1H), 7.36 (d, *J* = 8.6 Hz, 1H), 7.25–7.15 (m, 2H), 7.03–6.96 (m, 2H), 6.92 (td, *J* = 7.5, 1.1 Hz, 1H), 6.85 (dd, *J* = 8.1, 1.1 Hz, 1H), 6.37 (s, 1H). ^13^C NMR (101 MHz, DMSO-*d_6_*) δ 178.24, 141.64, 137.72, 133.06, 129.20, 124.76, 124.57, 124.11, 122.40, 121.79, 121.42, 115.81, 114.08, 113.94, 109.72, 74.70.

3-(7-bromo-1H-indol-3-yl)-3-hydroxyindolin-2-one (**3o**). Isolated yield: 68%; pale-yellow solid; ^1^H NMR (400 MHz, DMSO-*d_6_*) δ 11.19 (d, *J* = 2.7 Hz, 1H), 10.34 (s, 1H), 7.35 (d, *J* = 8.0 Hz, 1H), 7.27–7.16 (m, 3H), 7.03 (d, *J* = 2.6 Hz, 1H), 6.93 (td, *J* = 7.5, 1.1 Hz, 1H), 6.87 (dd, *J* = 7.7, 1.0 Hz, 1H), 6.81 (t, *J* = 7.8 Hz, 1H), 6.43 (s, 1H). ^13^C NMR (101 MHz, DMSO-*d_6_*) δ178.15, 141.69, 135.03, 132.98, 129.28, 126.65, 124.78, 124.65, 123.74, 121.83, 120.06, 119.98, 116.97, 109.76, 104.14, 74.75.

5-fluoro-3-hydroxy-3-(5-fluoro-1H-indol-3-yl)indolin-2-one (**3p**). Isolated yield: 90%; pale-yellow solid; ^1^H NMR (400 MHz, DMSO-*d_6_*) δ 11.09 (d, *J* = 2.7 Hz, 1H), 10.32 (s, 1H), 7.30 (dd, *J* = 8.9, 4.6 Hz, 1H), 7.15 (dd, *J* = 10.5, 2.6 Hz, 1H), 7.11–6.99 (m, 3H), 6.86 (m, 2H), 6.49 (s, 1H). ^13^C NMR (101 MHz, DMSO-*d_6_*) δ 178.26, 159.28, 157.65, 156.92, 155.36, 137.80 (d), 134.69 (d), 133.55, 125.66, 125.17 (d), 115.60-115.01 (q), 112.64-112.26 (q), 110.61 (d), 109.48 (q), 105.27 (d), 75.01 (d).

5-fluoro-3-hydroxy-3-(5-bromo-1H-indol-3-yl)indolin-2-one (**3q**). Isolated yield: 65%; pale-yellow solid; ^1^H NMR (400 MHz, DMSO-*d_6_*) δ 11.21 (d, *J* = 2.6 Hz, 1H), 10.34 (s, 1H), 7.72 (d, *J* = 2.0 Hz, 1H), 7.29 (d, *J* = 8.6 Hz, 1H), 7.14 (dd, *J* = 8.6, 2.0 Hz, 1H), 7.12–7.02 (m, 2H), 6.97 (d, *J* = 2.4 Hz, 1H), 6.86 (dd, *J* = 7.9, 4.4 Hz, 1H), 6.53 (s, 1H). ^13^C NMR (101 MHz, DMSO-*d_6_*) δ 178.17, 156.91, 137.80 (d), 135.59, 134.67 (d), 126.86, 125.22, 123.75, 123.16, 115.55 (d), 114.65, 113.64, 112.40 (d), 111.29, 110.62 (d), 74.97.

3-(5-bromo-1H-indol-3-yl)-5-chloro-3-hydroxyindolin-2-one (**3r**). Isolated yield: 63%; pale-yellow solid; ^1^H NMR (400 MHz, DMSO-*d_6_*) δ 11.22 (d, *J* = 2.7 Hz, 1H), 10.47 (s, 1H), 7.72 (d, *J* = 2.0 Hz, 1H), 7.30 (dd, *J* = 8.4, 2.6 Hz, 2H), 7.22 (d, *J* = 2.3 Hz, 1H), 7.14 (dd, *J* = 8.6, 2.0 Hz, 1H), 6.97 (d, *J* = 2.6 Hz, 1H), 6.89 (d, *J* = 8.2 Hz, 1H), 6.56 (s, 1H). ^13^C NMR (101 MHz, DMSO-*d_6_*) δ 177.85, 140.54, 135.60, 134.88, 129.14, 126.80, 125.80, 125.20, 124.74, 123.78, 123.07, 114.49, 113.69, 111.32 (2C), 74.76.

3-(5-bromo-1H-indol-3-yl)-5-bromo-3-hydroxyindolin-2-one (**3s**). Isolated yield: 81%; white solid; ^1^H NMR (400 MHz, DMSO-*d_6_*) δ 11.21 (d, *J* = 2.7 Hz, 1H), 10.48 (s, 1H), 7.71 (d, *J* = 2.1 Hz, 1H), 7.43 (dd, *J* = 8.3, 2.1 Hz, 1H), 7.37–7.21 (m, 2H), 7.14 (dd, *J* = 8.6, 2.0 Hz, 1H), 6.97 (d, *J* = 2.5 Hz, 1H), 6.84 (d, *J* = 8.2 Hz, 1H), 6.56 (s, 1H). ^13^C NMR (101 MHz, DMSO-*d_6_*) δ 177.71, 140.95, 135.59, 135.27, 131.99, 127.45, 126.78, 125.19, 123.79, 123.03, 114.50, 113.71, 113.48, 111.86, 111.33, 74.72.

3-hydroxy-5-iodo-3-(5-bromo-1H-indol-3-yl)indolin-2-one (**3t**). Isolated yield: 29%; pale-yellow solid; ^1^H NMR (400 MHz, DMSO-*d_6_*) δ 11.22 (d, *J* = 2.6 Hz, 1H), 10.47 (s, 1H), 7.69 (d, *J* = 2.1 Hz, 1H), 7.60 (dd, *J* = 8.1, 1.9 Hz, 1H), 7.48 (d, *J* = 1.8 Hz, 1H), 7.31 (d, *J* = 8.6 Hz, 1H), 7.16 (dd, *J* = 8.6, 2.0 Hz, 1H), 6.98 (d, *J* = 2.6 Hz, 1H), 6.75 (d, *J* = 8.1 Hz, 1H), 6.54 (s, 1H). ^13^C NMR (101 MHz, DMSO-*d_6_*) δ177.50, 141.40, 137.77, 135.57, 135.53, 132.95, 126.75, 125.16, 123.76, 122.95, 114.57, 113.70, 112.35, 111.30, 84.59, 74.54.

3-hydroxy-5-iodo-3-(5-fluoro-1H-indol-3-yl)indolin-2-one (**3u**). Isolated yield: 61%; pale-yellow solid; ^1^H NMR (400 MHz, DMSO-*d_6_*) δ 11.10 (d, *J* = 2.8 Hz, 1H), 10.44 (s, 1H), 7.57 (dd, *J* = 8.1, 1.8 Hz, 1H), 7.46 (d, *J* = 1.8 Hz, 1H), 7.31 (dd, *J* = 8.8, 4.7 Hz, 1H), 7.11 (dd, *J* = 10.4, 2.6 Hz, 1H), 7.03 (d, *J* = 2.6 Hz, 1H), 6.87 (td, *J* = 9.2, 2.6 Hz, 1H), 6.73 (d, *J* = 8.1 Hz, 1H), 6.49 (s, 1H). ^13^C NMR (101 MHz, DMSO-*d_6_*) δ 177.59, 157.65, 155.36, 141.40, 137.73, 135.65, 133.54, 132.95, 125.60-124.99 (t), 114.96 (d), 112.67 (d), 112.36, 109.51 (d), 105.06 (d), 84.57, 74.59. HRMS (ESI): *m*/*z* [M+Na]^+^ calcd for [C_16_H_10_FIN_2_O_2_Na]+: 430.9663, found: 430.9665.

3-hydroxy-5-iodo-3-(5-chloro-1H-indol-3-yl)indolin-2-one (**3v**). Isolated yield: 57%; white solid; ^1^H NMR (400 MHz, DMSO-*d_6_*) δ 11.20 (s, 1H), 10.46 (s, 1H), 7.59 (dd, *J* = 8.1, 1.8 Hz, 1H), 7.51 (d, *J* = 2.1 Hz, 1H), 7.47 (d, *J* = 1.8 Hz, 1H), 7.34 (d, *J* = 8.6 Hz, 1H), 7.03 (dd, *J* = 8.6, 2.1 Hz, 1H), 7.00 (d, *J* = 2.3 Hz, 1H), 6.74 (d, *J* = 8.1 Hz, 1H), 6.53 (s, 1H). **^13^C** NMR (101 MHz, DMSO-*d_6_*) δ 177.53, 141.40, 137.77, 135.56, 135.35, 132.96, 126.01, 125.34, 123.26, 121.26, 119.85, 114.65, 113.24, 112.36, 84.60, 74.55. HRMS (ESI): *m*/*z* [M+Na]+ calcd for [C_16_H_10_ClIN_2_O_2_Na]^+^: 446.9368, found: 446.9383.

3-hydroxy-5-iodo-3-(5-iodo-1H-indol-3-yl)indolin-2-one (**3w**). Isolated yield: 36%; white solid; ^1^H NMR (400 MHz, DMSO-*d_6_*) δ 11.18 (s, 1H), 10.46 (s, 1H), 7.91 (s, 1H), 7.60 (dd, *J* = 8.2, 1.8 Hz, 1H), 7.51 (s, 1H), 7.30 (d, *J* = 8.6 Hz, 1H), 7.20 (d, *J* = 8.6 Hz, 1H), 6.93 (s, 1H), 6.75 (d, *J* = 8.1 Hz, 1H), 6.52 (s, 1H). ^13^C NMR (101 MHz, DMSO-*d_6_*) δ 177.48, 141.38, 137.73, 135.90, 135.56, 132.94, 129.24, 129.15, 127.65, 124.63, 114.25, 114.16, 112.30, 84.53, 82.66, 74.56. HRMS (ESI): *m*/*z* [M-H]^−^ calcd for [C_16_H_9_I_2_N_2_O^2^]^−^: 514.8759, found: 514.9234.

3-hydroxy-5-iodo-3-(5-methyl-1H-indol-3-yl)indolin-2-one (**3x**). Isolated yield: 40%; white solid; ^1^H NMR (400 MHz, DMSO-*d_6_*) 10.85 (d, *J* = 2.6 Hz, 1H), 10.43 (s, 1H), 7.53 (dd, *J* = 8.1, 1.8 Hz, 1H), 7.40 (d, *J* = 1.9 Hz, 1H), 7.18 (d, *J* = 8.3 Hz, 1H), 7.16–7.11 (m, 1H), 6.95 (d, *J* = 2.6 Hz, 1H), 6.82 (dd, *J* = 8.3, 1.7 Hz, 1H), 6.71 (d, *J* = 8.1 Hz, 1H), 6.41 (s, 1H), 2.23 (s, 3H). ^13^C NMR (101 MHz, DMSO-*d_6_*) δ 177.79, 141.40, 137.51, 136.20, 135.21, 132.89, 126.90, 125.00, 123.59, 122.84, 119.80, 114.26, 112.26, 111.36, 84.48, 74.83, 21.49.

3-hydroxy-5-iodo-3-(5-methoxy-indol-3-yl)indolin-2-one (**3y**). Isolated yield: 56%; white solid; ^1^H NMR (400 MHz, DMSO-*d_6_*) 10.87 (d, *J* = 2.7 Hz, 1H), 10.45 (s, 1H), 7.58 (dd, *J* = 8.1, 1.8 Hz, 1H), 7.48 (d, *J* = 1.8 Hz, 1H), 7.23 (d, *J* = 8.8 Hz, 1H), 7.02 (d, *J* = 2.6 Hz, 1H), 6.81 (d, *J* = 2.5 Hz, 1H), 6.76 (d, *J* = 8.1 Hz, 1H), 6.70 (dd, *J* = 8.8, 2.5 Hz, 1H), 6.45 (s, 1H), 3.62 (s, 3H). ^13^C NMR (101 MHz, DMSO-*d_6_*) 177.66, 152.81, 141.41, 137.50, 135.99, 132.95, 131.96, 125.10, 124.25, 114.29, 112.18(2C), 110.96, 102.32, 84.42, 74.77, 55.17.

### 4.3. Biological Assay

Five plant pathogenic fungi, including *Rhizoctonia solani* Kühn, *Pyricularia oryzae* Cav., *Colletotrichum gloeosporioides* Penz., *Botrytis cinerea* Pers.:Fr., and *Bipolaris maydis* (Nishik.) Shoemaker, were selected for testing the efficacy of newly synthesized compounds. These fungi are detrimental to crops such as grains, fruits, and vegetables, exhibiting a variety of harmful effects. The control agents for comparison were the agricultural fungicides carvacrol (CA) and phenazine-1-carboxylic acid (shenqinmycin, PCA). The in vitro antifungal activity of these compounds was assessed using the mycelial growth rate method. The in vivo antifungal activity of compound **3u** against *R. solani* was evaluated through a modified broad bean leaf bioassay, following the procedures outlined in the Chinese National Agricultural Industry Standard [48]. Detailed test procedures are available in the Supplementary Materials.

## 5. Conclusions

A series of twenty-five novel 3-indolyl-3-hydroxy oxindole derivatives (**3a**–**3y**) were successfully synthesized and evaluated for their antifungal activity against plant pathogenic fungi, with most compounds exhibiting moderate to excellent inhibitory effects; notably, **3t**, **3u**, **3v**, and **3w** emerged as potent broad-spectrum candidates, matching or surpassing the efficacies of commercial fungicides carvacrol (CA) and phenazine-1-carboxylic acid (PCA). Compound **3u** demonstrated exceptional activity against *Rhizoctonia solani* (EC50 = 3.44 mg/L), outperforming both CA (7.38 mg/L) and PCA (11.62 mg/L). The practical potential of **3u** was further validated through an in vivo broad bean leaf bioassay, where it achieved control efficacies of 58.41% and 81.93% at concentrations of 100 and 200 mg/L, respectively, slightly lower than PCA (71.62% and 83.84%) but confirming its significant therapeutic potential under elevated dosage conditions. Structure–activity relationship (SAR) analysis revealed that halogen substituents (I, Cl, and Br) at position 5 of the 3-hydroxy-2-oxindole and indole rings play a critical role in enhancing antifungal potency. These findings highlight the potential of 3-indolyl-3-hydroxy oxindole derivatives, particularly compound **3u**, as promising lead compounds for next-generation agricultural fungicides, warranting further studies on mechanistic investigations, field trials, and structural optimization for practical applications in plant disease management.

## Figures and Tables

**Table 1:** Preliminary in vitro antifungal activity of compounds against five fungi at 50 mg/L.

	Average Inhibition Rate ± SD (%) (*n* = 3) ^a^				
	*R. solani*	*P. oryzae*	*C. gloeosporioides*	*B. cinerea*	*B. maydis*
**3a**	29.16 ± 3.32	17.48 ± 0.03	19.72 ± 0.01	24.26 ± 0.03	49.54 ± 0.01
**3b**	19.20 ± 0.06	11.90 ± 0.05	9.39 ± 0.01	27.28 ± 0.01	42.94 ± 0.04
**3c**	22.23 ± 0.02	31.58 ± 0.02	23.93 ± 0.02	23.93 ± 0.02	57.99 ± 0.05
**3d**	24.21 ± 0.02	45.63 ± 0.01	26.29 ± 0.01	38.84 ± 0.01	50.12 ± 0.01
**3e**	29.44 ± 0.02	53.67 ± 0.01	33.55 ± 0.01	61.86 ± 0.01	61.15 ± 0.03
**3f**	37.03 ± 0.02	34.18 ± 0.03	25.84 ± 0.01	35.58 ± 0.01	49.88 ± 0.01
**3g**	37.92 ± 0.01	24.22 ± 0.01	39.28 ± 0.01	52.67 ± 0.01	57.28 ± 0.03
**3h**	28.12 ± 1.29	40.29 ± 0.31	51.01 ± 0.01	80.82 ± 0.02	38.68 ± 1.56
**3i**	52.72 ± 0.01	23.26 ± 0.03	55.87 ± 0.01	80.42 ± 0.01	58.45 ± 0.01
**3j**	39.79 ± 0.00	37.79 ± 0.01	43.34 ± 0.01	52.67 ± 0.00	36.09 ± 0.01
**3k**	33.90 ± 0.08	17.68 ± 0.03	41.57 ± 0.01	18.90 ± 0.04	35.56 ± 0.01
**3l**	56.78 ± 0.02	12.62 ± 3.79	35.92 ± 0.01	26.09 ± 0.03	54.79 ± 0.01
**3m**	35.56 ± 0.01	9.88 ± 0.02	17.31 ± 0.02	19.15 ± 0.03	41.21 ± 0.01
**3n**	48.01 ± 0.01	31.11 ± 0.00	29.11 ± 0.02	55.01 ± 0.01	58.01 ± 0.00
**3o**	33.15 ± 0.01	52.61 ± 0.01	21.44 ± 0.01	54.58 ± 0.00	59.66 ± 0.01
**3p**	68.43 ± 0.01	31.32 ± 0.00	21.22 ± 0.01	38.81 ± 0.01	31.12 ± 0.02
**3q**	47.46 ± 0.01	39.83 ± 0.02	24.25 ± 0.02	38.26 ± 0.08	53.76 ± 0.03
**3r**	27.58 ± 0.04	55.16 ± 0.02	42.25 ± 0.01	52.20 ± 0.03	73.51 ± 0.03
**3s**	57.95 ± 0.01	57.71 ± 0.01	50.10 ± 0.01	64.65 ± 0.01	75.51 ± 0.04
**3t**	82.48 ± 0.02	77.66 ± 0.00	61.62 ± 0.01	55.43 ± 0.01	86.56 ± 0.01
**3u**	100.00 ± 0.00	75.36 ± 0.01	46.73 ± 0.00	91.05 ± 0.01	81.13 ± 0.03
**3v**	77.39 ± 0. 49	83.26 ± 0.01	55.67 ± 0.01	67.25 ± 0.01	92.22 ± 0.00
**3w**	87.37 ± 0.02	81.42 + 0.01	66.67 ± 0.00	73.08 ± 0.01	89.85 ± 0.01
**3x**	50.12 ± 0.00	50.79 ± 0.00	50.56 ± 0.03	62.56 ± 0.02	66.81 ± 0.00
**3y**	23.52 ± 0.01	29.51 ± 0.02	18.59 ± 0.01	24.65 ± 0.00	49.22 ± 0.02
CA	91.56 ± 1.33	74.94 ± 0.42	62.65 ± 2.13	84.38 ± 4.55	64.36 ± 2.73
PCA	81.07 ± 0.89	53.64 ± 3.57	77.96 ± 4.53	81.86 ± 2.72	98.01 ± 0.74

^a^ CA, carvacrol; PCA, phenazine-1-carboxylic acid (shenqinmycin). The text in red indicates an inhibition rate exceeding 60%.

**Table 2:** EC_50_ values (mg/L) of selected compounds against *R. solani*, *P. oryzae*, *B. cinerea*, and *B. maydis* in vitro.

Compound	Regression Equation	R^2^	EC_50_ (mg/L, 95% CI) ^a^	EC_50_ (µM, 95% CI) ^a^
*R. solani*				
**3t**	y = −1.72 + 1.61x	0.921	12.69 (10.79~14.97)	27.05 (23.00~31.91)
**3u**	y = −0.85 + 1.61x	0.985	3.44 (3.01~3.93)	8.43 (7.37~9.63)
**3v**	y = −1.62 + 1.36x	0.925	16.16 (14.36~18.19)	38.06 (33.82~42.84)
**3w**	y = −1.05 + 1.06x	0.884	9.93 (7.91~12.95)	19.24 (15.33~25.09)
CA	y = −1.11 + 0.55x	0.997	7.38 (6.04~8.78)	49.13 (40.21~58.45)
PCA	y = −1.47 + 0.60x	0.994	11.62 (9.92~13.49)	51.83 (44.24~60.17)
*P. oryzae*				
**3v**	y = −1.5 + 1.28x	0.973	14.72 (13.16~16.53)	34.67 (30.99~38.93)
**3w**	y = −1.65 + 1.39x	0.983	15.69 (14.12~17.50)	30.40 (27.36~33.91)
CA	y = −2.16 + 1.54x	0.977	25.30 (22.63~28.40)	170.00 (150.65~189.05)
PCA	y = −2.52 + 1.39x	0.942	64.53 (53.73~84.63)	287.81 (239.64~377.46)
*B. cinerea*				
**3h**	y = −1.46 + 1.35x	0.910	12.05 (9.88~14.73)	40.34 (33.07~49.31)
**3i**	y = −1.94 + 1.58x	0.914	16.54 (13.82~19.81)	48.20 (40.27~57.72)
**3u**	y = −1.47 + 1.37x	0.975	11.89 (10.68~13.25)	29.13 (26.17~32.46)
CA	y = −1.98 + 2.18x	0.911	21.36 (19.97~23.64)	142.19 (132.94~157.37)
PCA	y = −1.32 + 2.00x	0.938	14.75 (12.93~16.17)	65.79 (57.67~72.12)
*B. maydis*				
**3t**	y = −0.90 + 0.80x	0.939	13.62 (10.97~17.23)	29.04 (23.39~36.73)
**3u**	y = −1.69 + 1.53x	0.960	12.76 (11.43~14.17)	31.26 (28.00~34.72)
**3v**	y = −1.21 + 1.09x	0.923	13.47 (11.81~15.42)	31.72 (27.81~33.37)
**3w**	y = −0.91 + 0.91x	0.906	10.55 (7.44~15.38)	20.44 (14.42~29.80)
CA	y = −1.08 + 0.86x	0.947	18.58 (16.98~19.74)	123.69 (113.03~131.41)
PCA	y = −0.59 + 1.34x	0.965	3.09 (1.87~4.29)	13.78 (8.34~19.13)

^a^ Average of three replicates.

**Table 3:** Curative and protective activities of **3u** against *R. solani* in vivo ^a,b.^

Treatment	Concentration (mg/L)	Curative Effect		Protective Effect	
		Lesion Area (cm^2^ ± SD)	Control Efficacy (%)	Lesion Area (cm^2^ ± SD)	Control Efficacy (%)
PCA	100	3.53 ± 0.44 c	71.86	5.61 ± 0.71 d	53.43
	200	2.01 ± 0.64 d	84.08	4.67 ± 0.33 e	61.18
**3u**	100	5.18 ± 0.74 b	58.62	10.07 ± 0.56 b	16.30
	200	2.22 ± 0.65 d	82.26	8.62 ± 0.76 c	28.46
CK	0	12.60 ± 0.64 a		12.04 ± 0.43 a	

^a^ Values are mean ± SD of three replicates. ^b^ Statistical analysis was conducted by a SPSS 27.0 software. Different letters indicate significant differences at *p* < 0.05 in the same group. The alphabetical order is consistent with the long to short order of the lesion area.

## Data Availability

Data are contained within the article and Supplementary Materials.

## Supplementary Materials

The following supporting information can be downloaded at https://www.mdpi.com/article/10.3390/molecules30051079/s1. Figure S1: In vitro antifungal activity evaluation; Figure S2: In vivo antifungal activity evaluation; Table S1. Preliminary in vitro antifungal activity and statistical analysis of compounds against five fungi at 50 mg/L; Figure S3: Copies of ^1^H, ^13^C NMR, and HRMS spectra of compounds.
