# Supplementary material for: Synthesis, Characterization, and Evaluation of the Antifungal Properties of 3-Indolyl-3-Hydroxy Oxindole Derivatives Against Plant Pathogenic Fungi
Source: Molecules. 2025 Feb 26;30(5):1079. doi: 10.3390/molecules30051079 (PMC11901744; doi:10.3390/molecules30051079)
Supplement: Supplementary file 1 [file molecules-30-01079-s001.zip › molecules-3435100-supplementary.pdf]

Supporting Information

Synthesis, characterization, and evaluation of the antifungal properties of 3-indolyl-3-hydroxy oxindole derivatives against plant pathogenic fungi

Zhiqiang Bai [1, 2 †], Kunrong Dang [1 †], Jinrui Tang [1], Rongjing Yang [1], Liming Fan [1], Qiu Li [1], Yue Yang [1], Min Ye [1]*, Fawu Su [1]*

[1] State Key Laboratory for Conservation and Utilization of Bio-Resources in Yunnan, College of Plant Protection, Yunnan Agricultural University, Kunming 650201, China; baizhiqiang10@mails.ucas.ac.cn(B.Z.); dang_kr@163.com (D.K.); tjinrui2020@163.com (T.J); YRonJING163.com (Y.R.); fanliming1976@163.com (F.L.); qiuli0199@163.com (L.Q.); yangyueaq@sina.com (Y.Y.); yeminpc@126.com (Y.M.); su_faw@126.com (S.F.)

[2] College of science, Yunnan Agricultural University, Kunming 650201, China

* Correspondence: yeminpc@126.com; su_faw@126.com

† These authors contributed equally to this work.

Table of Content

1. *In vitro* antifungal activity evaluation …….. S-2

2. In vivo antifungal activity evaluation …….. S-4

3. Table S1. Preliminary in vitro antifungal activity and statistical analysis of compounds against five fungi at 50 mg/L………………………………….........S-5

4. ^{1}H NMR, ^{13}C NMR, and HRMS Spectra of Synthesized Compounds………......S-6

In vitro antifungal activity evaluation

The *in vitro* antifungal activity of the synthesized compounds was evaluated by using the mycelial growth inhibition method against five plant pathogenic fungi, including *Rhizoctonia solani*, *Pyricularia oryzae*, *Colletotrichum gloeosporioides*, *Botrytis cinerea*, and *Bipolaris maydis*, were selected for testing the efficacy of newly synthesized compounds. The mycelial growth rate method was employed to assess the antifungal activity of compounds against various plant pathogenic fungi. The specific procedure was as follows: potato dextrose agar (PDA) culture medium was prepared by mixing water, potato extract, glucose, and agar in a ratio of 1000 : 20 : 20 : 20. The mixture was then divided into conical flasks and sterilized at 121 ℃ for 30 minutes. Next, the tested compounds were individually dissolved in dimethyl sulfoxide (DMSO) and prepared at concentrations of 1000 μg/mL and 5000 μg/mL. Using a pipette, 600 μL of the compound solution was added to a conical flask containing 60 mL of the culture medium, thoroughly mixed, and resulting in toxic culture mediums with concentrations of 10 μg/mL and 50 μg/mL, respectively. A toxic culture medium containing DMSO at an equal concentration served as the blank control, while commercial pesticides carvacrol and phenazine-1-carboxylic acid (shenqinmycin) were used as positive controls. Each culture medium was poured into a culture dish, allowed to cool, and then inoculated with fungal blocks of 0.5 cm diameter. For all experiments, three parallel groups were set up or the experiments were repeated three times to ensure consistency. The culture dishes were placed in a dark incubator for cultivation. Colony diameter measurements were taken using the cross method, with the mycelium in the blank control growing to the edge of the culture dish. The average value was used to determine the inhibition rate of each compound. The calculation of the Inhibition rate was performed using the following formula:

$$\mathrm{Ir} = \frac{\mathrm{Cc}-\mathrm{Tc}}{\mathrm{Cc}-0.5} \times 100 \qquad (1)$$

In formula (1): Ir represents the inhibition rate, Cc represents the diameter of the colony in the control group, Tc represents the diameter of the colony in the treatment group, and 0.5 is the diameter of the inoculated fungi mass.

Based on the results of *in vitro* antifungal activity, the more active compounds were selected to determine their median effective concentration (EC_{50}) according to the same method described above. The stock solution was mixed with the autoclaved PDA medium to prepare a set of mediums containing 64, 32, 16, 8, 4, 2, 1 μg/mL of the tested

compound. Similarly, 1% DMSO in culture medium was used as a blank control. Each test was performed in triplicate. EC_{50} values and their confidence intervals at 95% probability (95% CI) were calculated by using the basic EC_{50} program version SPSS 27.0. The statistical analysis was performed using one-way ANOVA followed by Duncan's post hoc test, and significant differences are indicated with appropriate annotations ($P < 0.05$).

Pure cultures of *R. solani*, *P. oryzae*, *C. gloeosporioides*,*B. cinerea* and *B. maydis* were obtained from the State Key Laboratory for Conservation and Utilization of Bio-Resources in Yunnan, College of Plant Protection, Yunnan Agricultural University (Kunming, China).

In vivo antifungal activity evaluation

In vivo antifungal efficacy of compound 3u against *R. solani* was evaluated through a modified broad bean leaf bioassay, following the procedures outlined in the Chinese National Agricultural Industry Standard NY/T 1156.5-2006, Pesticides guidelines for laboratory bioactivity tests, Part 5: Detached leaf test for fungicide inhibition of *Rhizoctonia solani* on faba bean. Healthy broad bean leaves of uniform size were collected, gently washed with distilled water, and air-dried at room temperature until no surface moisture remained. Compound 3u was dissolved in dimethyl sulfoxide (DMSO) and diluted with 0.05% (v/v) Tween-80 aqueous solution to obtain final concentrations of 100 and 200 mg/L. Control groups included a blank control (0.05% Tween-80 solution without compounds) and a positive control (phenazine-1-carboxylic acid with concentrations of 100 and 200 mg/L). For protective treatment, leaves were fully immersed in the test solutions for 5 s, air-dried, and inoculated with fungal plugs after 24 h. For curative treatment, leaves were first inoculated with fungal plugs, followed by compound application after 24 h. Fungal plugs (0.5 cm diameter) from actively growing *R. solani* cultures were centrally placed on each leaf, with each treatment group consisting of 15 leaves. Treated leaves were placed in 500 mL transparent containers lined with sterile moist gauze (3 leaves/container) and maintained at 27°C with 12 h light/dark cycles. Disease severity was recorded when lesion coverage exceeded 80% in blank controls. The lesion area was quantified using image analysis software, and control efficacy (%) was calculated according to Formula (2), with results expressed as mean ± SD (retaining two decimal places).

$$P = \frac{D_0 - D_1}{D_1} \times 100 \qquad (2)$$

P-----control effect

D_0---diameter of blank control lesion

D_1---diameter of disease spot treated with medicine

The statistical analysis was conducted using SPSS 27.0 software. The analysis involved one-way ANOVA followed by Duncan’s post hoc test and single sample T test. Significant differences are indicated with appropriate annotations ($P < 0.05$).

Table S1. Preliminary in vitro antifungal activity and statistical analysis of compounds against five fungi at 50 mg/L.

	Average Inhibition Rate ± SD (%) (n = 3) [a, b]				
	R. solani	*P. oryzae*	*C. gloeosporioides*	*B. cinerea*	*B. maydis*
3a	29.16±3.32mn	17.48±3.22k	19.72±0.57jk	24.26±3.32i	49.54±0.52k
3b	19.20±5.62r	11.90±4.77l	9.39±0.82o	27.28±1.38hi	42.94±3.64l
3c	22.23±2.41qr	31.58±2.50hi	23.93±2.38l	23.93±1.00h	57.99±4.99hi
3d	24.21±1.52opq	45.63±0.69f	26.29±1.07l	38.84±1.21g	50.12±1.00k
3e	29.44±2.37mn	53.67±1.13de	33.55±0.59ij	61.86±1.05e	61.15±2.57gh
3f	37.03±2.19jkl	34.18±2.93h	25.84±0.90l	35.58±0.58g	49.88±1.37k
3g	37.92±1.32jk	24.22±0.88j	39.28±1.26h	52.67±0.76f	57.28±2.74hij
3h	28.12±1.29no	40.29±0.31g	51.01±1.34e	80.82±1.56b	38.68±1.56mn
3i	52.72±1.12h	23.26±3.02j	55.87±1.26d	80.42±0.77b	58.45±0.89hi
3j	39.79±0.36j	37.79±0.86g	43.34±0.83g	52.67±0.38f	36.09±0.62n
3k	33.90±7.71kl	17.68±2.93k	41.57±1.28gh	18.90±4.01j	35.56±1.48n
3l	56.78±1.50g	12.62±3.79l	35.92±1.25i	26.09±3.34i	54.79±0.59ij
3m	35.56±1.61jkl	9.88±1.69l	17.31±1.87n	19.15±2.91j	41.21±0.51ml
3n	48.01±0.81i	31.11±0.25hi	29.11±2.11k	55.01±0.95f	58.01±0.41hi
3o	33.15±1.36lm	52.61±0.80de	21.44±1.49m	54.58±0.34f	59.66±1.36h
3p	68.43±0.75f	31.32±0.11hi	21.22±0.72m	38.81±0.57g	31.12±2.28o
3q	47.46±0.66i	39.83±1.90g	24.25±1.95l	38.26±8.46g	53.76±3.19j
3r	27.58±4.29nop	55.16±1.72cd	42.25±1.11g	52.20±3.49f	73.51±2.62e
3s	57.95±0.67g	57.71±0.73c	50.10±1.40e	64.65±0.89e	75.51±4.49e
3t	82.48±2.21d	77.66±041b	61.62±0.69c	55.43±1.25f	86.56±1.35c
3u	100.00±0.00a	75.36±0.77b	46.73±0.41f	91.05±1.07a	81.13±3.34d
3v	77.39±0.49e	83.26±0.59a	55.67±0.85d	67.25±0.65d	92.22±0.14b
3w	87.37±0.02c	81.42+0.69a	66.67±0.35b	73.08±0.49c	89.85±0.57bc
3x	50.12±0.20hi	50.79±0.21e	50.56±2.73e	62.56±1.86e	66.81±0.33f
3y	23.52±1.34pq	29.51±1.67i	18.59±0.79n	24.65±0.46i	49.22±2.50k
CA	91.56±1.33b	74.94±0.42b	62.65±2.13c	84.38±4.55b	64.36±2.73fg
PCA	81.07±0.89ed	53.64±3.57de	77.96±4.53a	81.86±2.72b	98.01±0.74a

[a] CA, Carvacrol; PCA, phenazine-1-carboxylic acid (shenqinmycin). [b] Compared within the same column, values marked with different lowercase letters denote statistically significant differences among the test compounds ($P < 0.05$). The text in red indicates an inhibition rate exceeding 60%; means±SD.

^{1}H NMR, ^{13}C NMR and HRMS Spectra of Synthesized Compounds

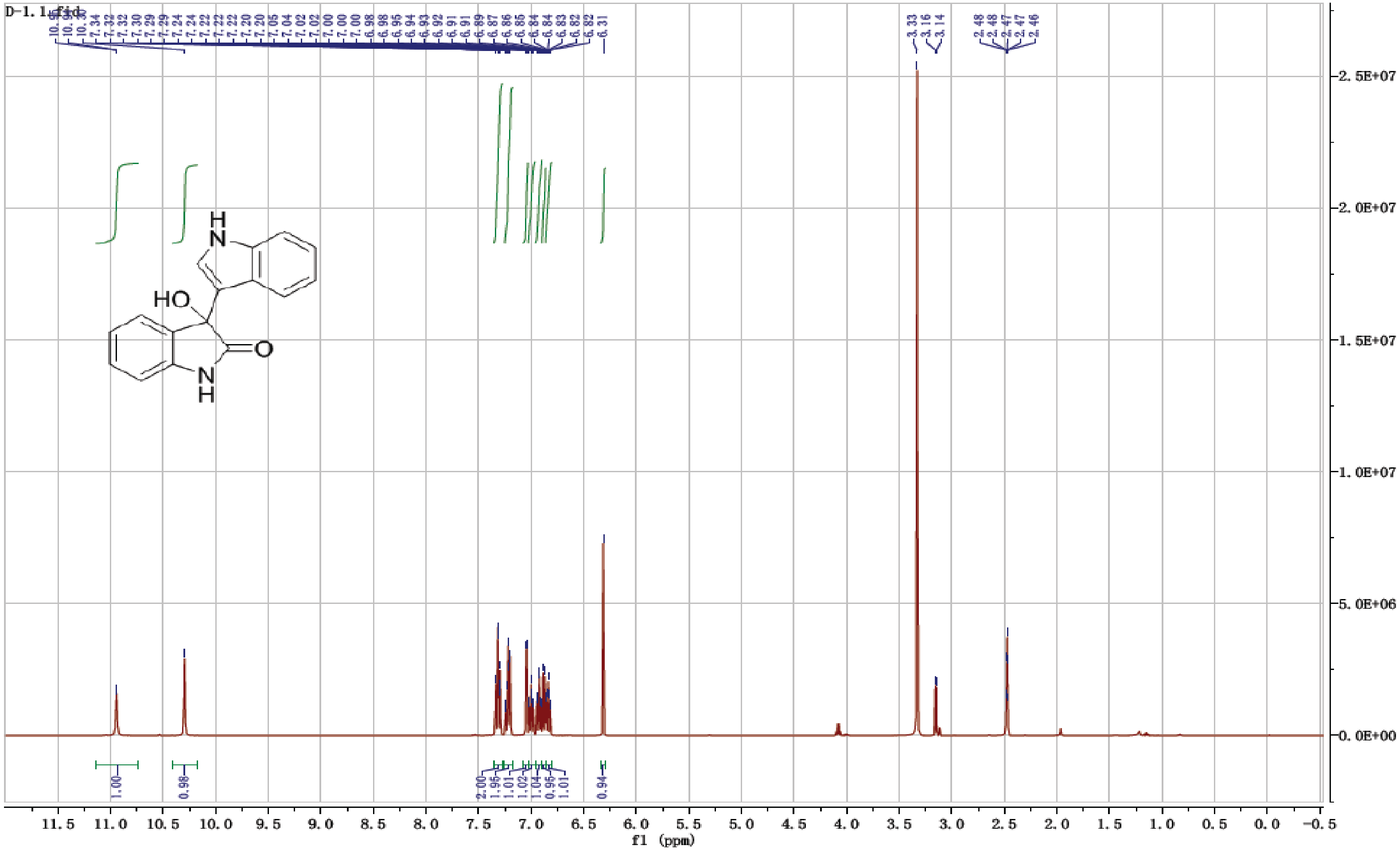

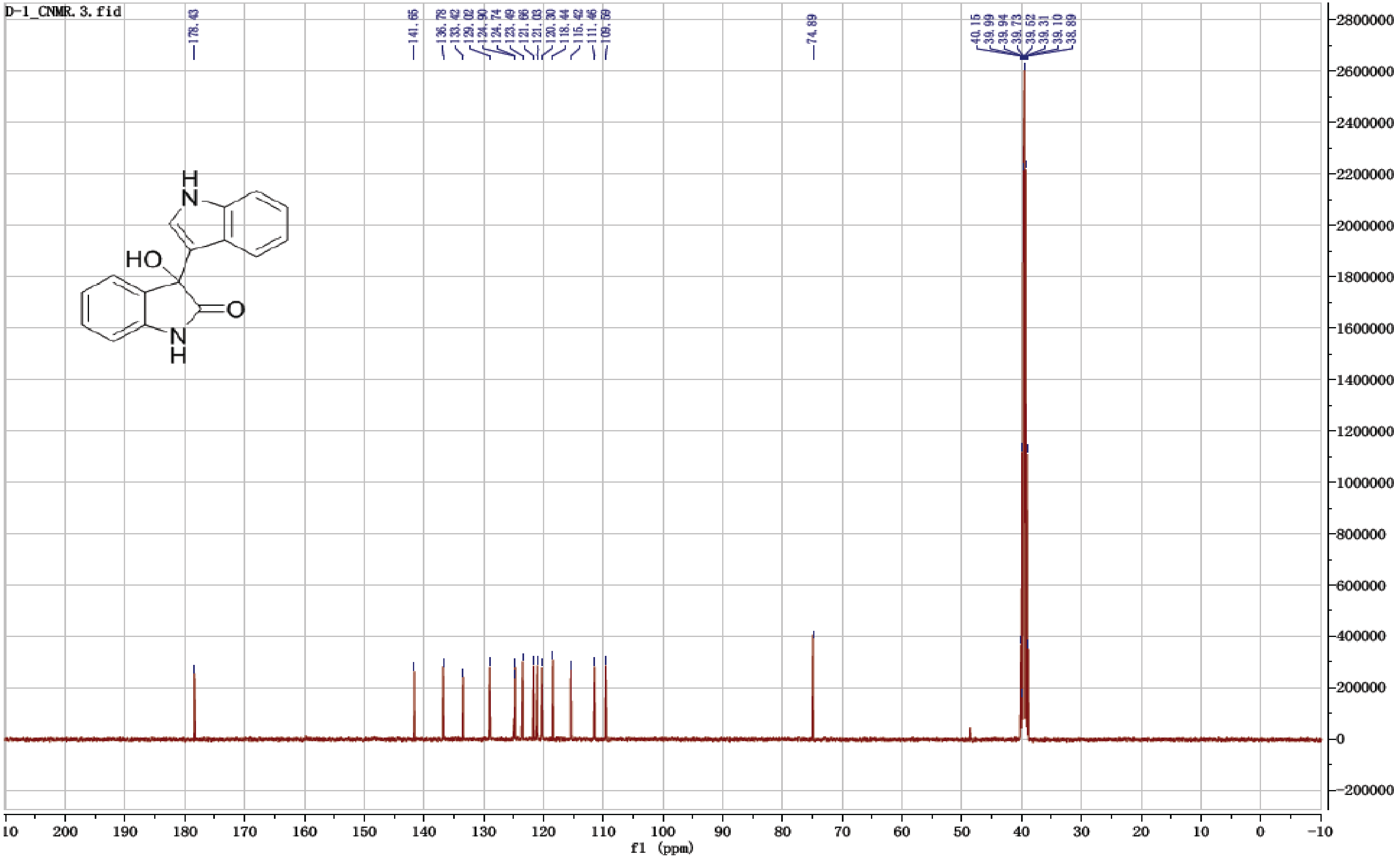
D-1_CNMR.3.fid
178.43
141.66
136.78
133.42
129.02
124.90
124.74
123.49
121.86
121.03
120.30
118.44
115.42
111.46
109.69
74.89
40.15
39.99
39.94
39.73
39.52
39.31
39.10
38.89
HO
f1 (ppm)

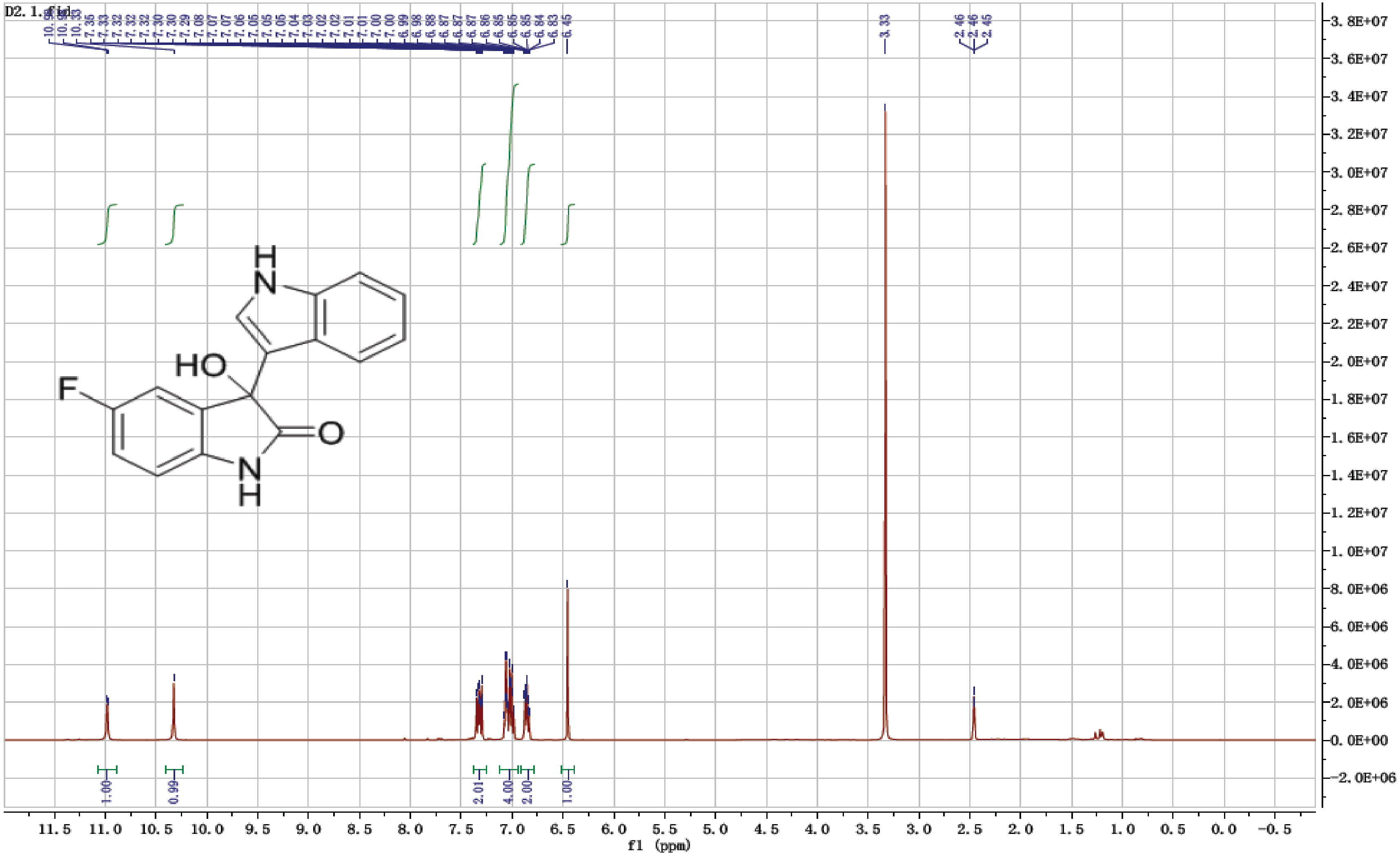
D2. 1. fid
10.98
10.98
10.33
7.35
7.33
7.32
7.32
7.32
7.30
7.30
7.29
7.08
7.07
7.07
7.06
7.05
7.05
7.05
7.04
7.03
7.02
7.02
7.01
7.01
7.00
7.00
6.99
6.98
6.88
6.87
6.87
6.87
6.86
6.85
6.85
6.85
6.84
6.83
6.45
3.33
2.46
2.46
2.45
1.00
0.99
2.01
4.00
2.00
1.00
F
HO
O
N
H
N
H
f1 (ppm)

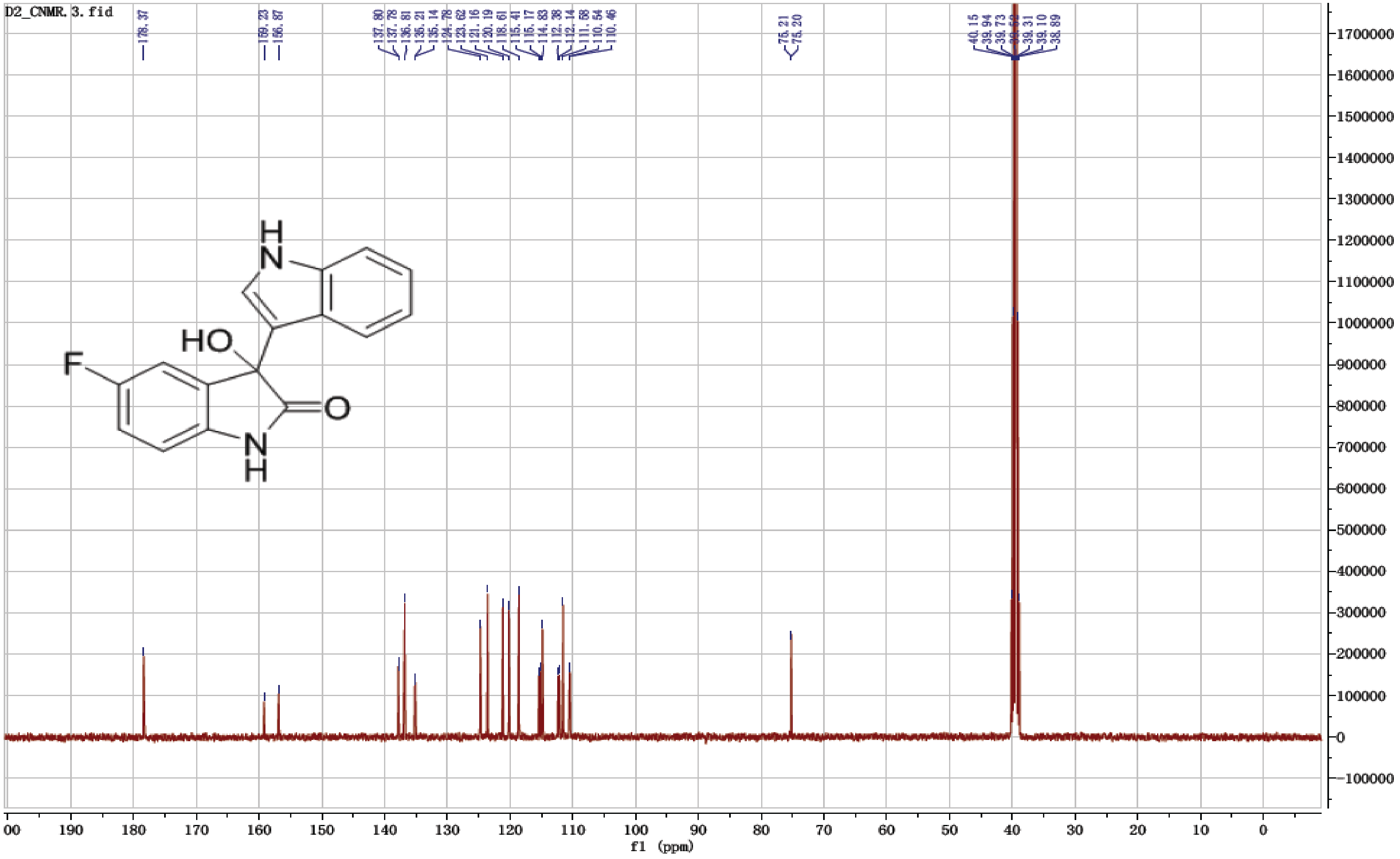
D2_CNMR.3.fid
178.37
159.23
156.87
137.80
137.78
136.81
135.21
135.14
124.78
123.62
121.16
120.19
118.61
115.41
115.17
114.83
112.38
112.14
111.58
110.54
110.46
75.21
75.20
40.15
39.94
39.73
39.31
39.10
38.89
HO
F
O
N
H
f1 (ppm)

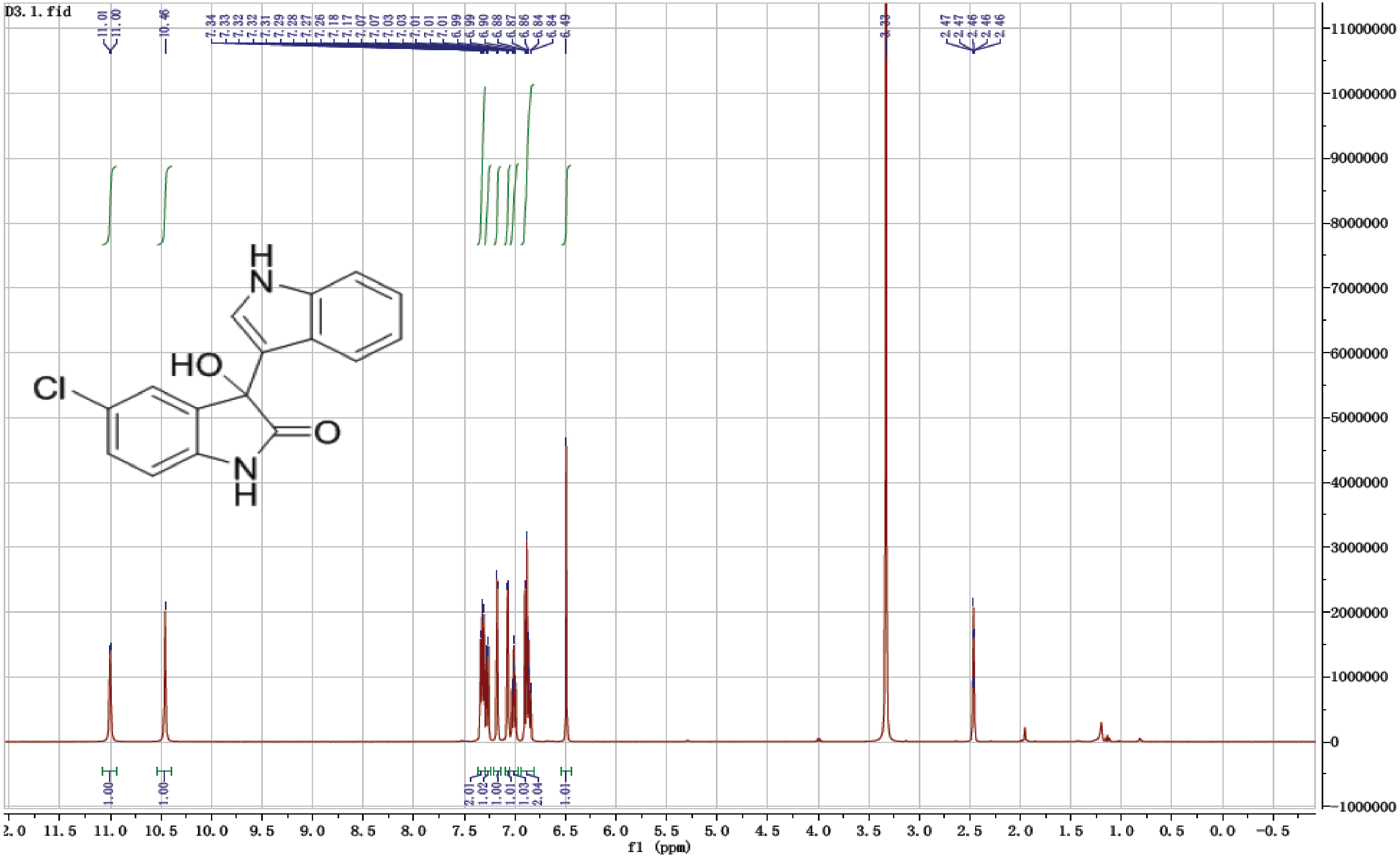
D3.1.fid
f1 (ppm)

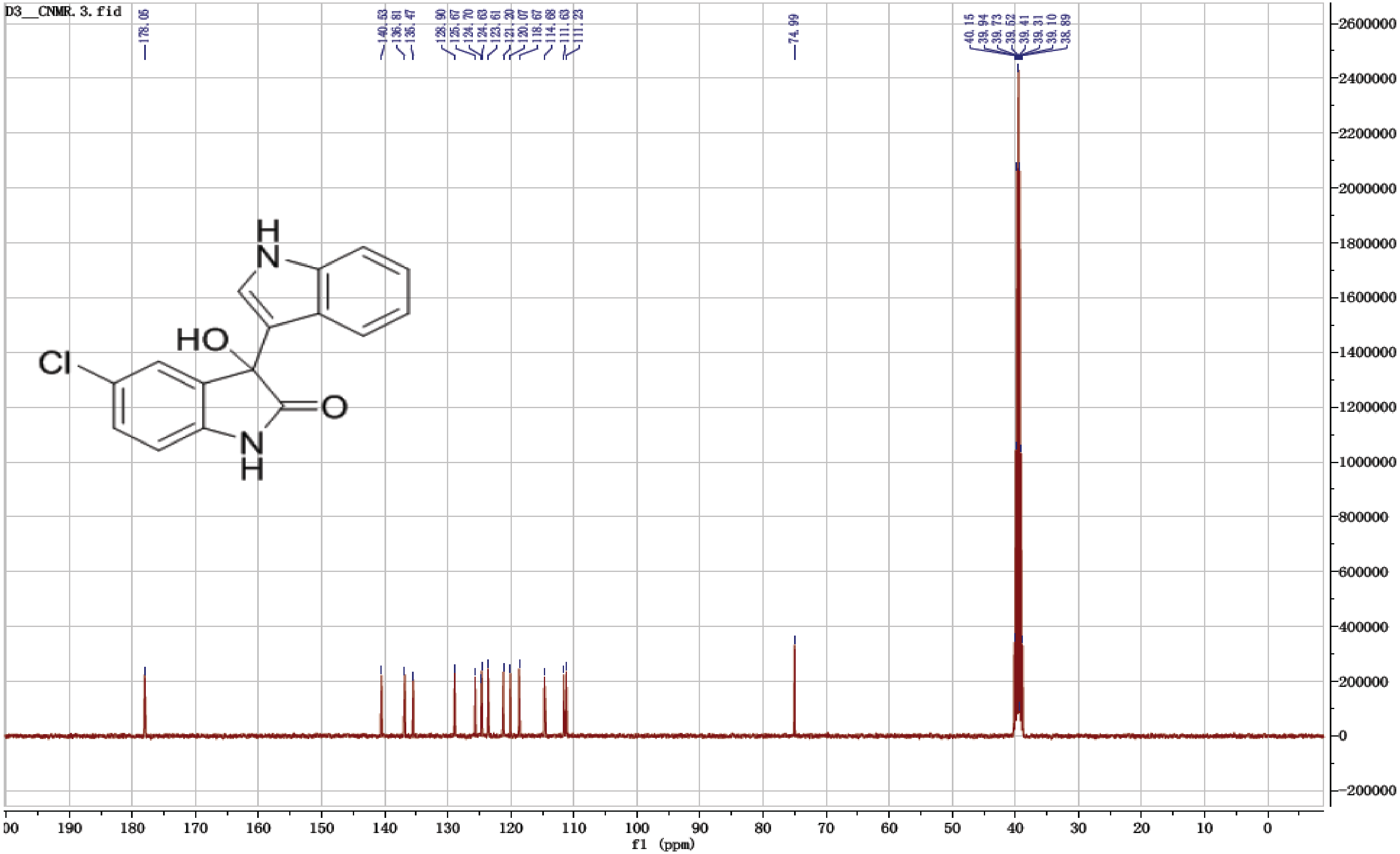

D3__CNMR.3.fid
178.05
140.53
136.81
135.47
128.90
125.67
124.70
124.63
123.61
121.30
120.07
118.67
114.68
111.63
111.23
74.99
40.15
39.94
39.73
39.52
39.41
39.31
39.10
38.89
HO
Cl
O
N
H
f1 (ppm)

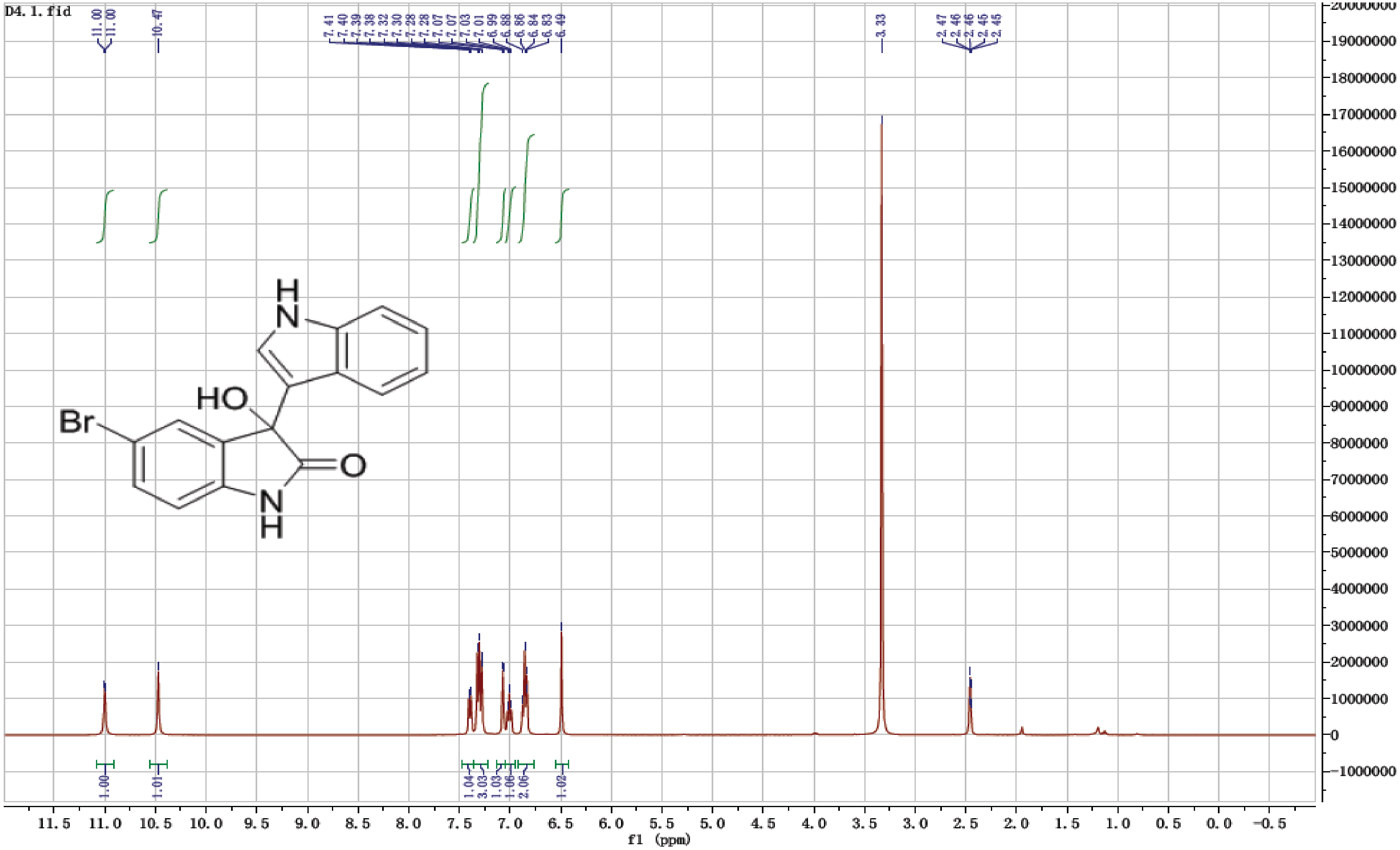

D4.1.fid
11.00
11.00
10.47
7.41
7.40
7.39
7.38
7.32
7.30
7.28
7.28
7.07
7.07
7.03
7.01
6.99
6.88
6.86
6.84
6.83
6.49
3.33
2.47
2.46
2.46
2.45
2.45
HO
Br
H
N
O
1.00
1.01
1.04
3.03
1.03
1.06
2.06
1.02
f1 (ppm)

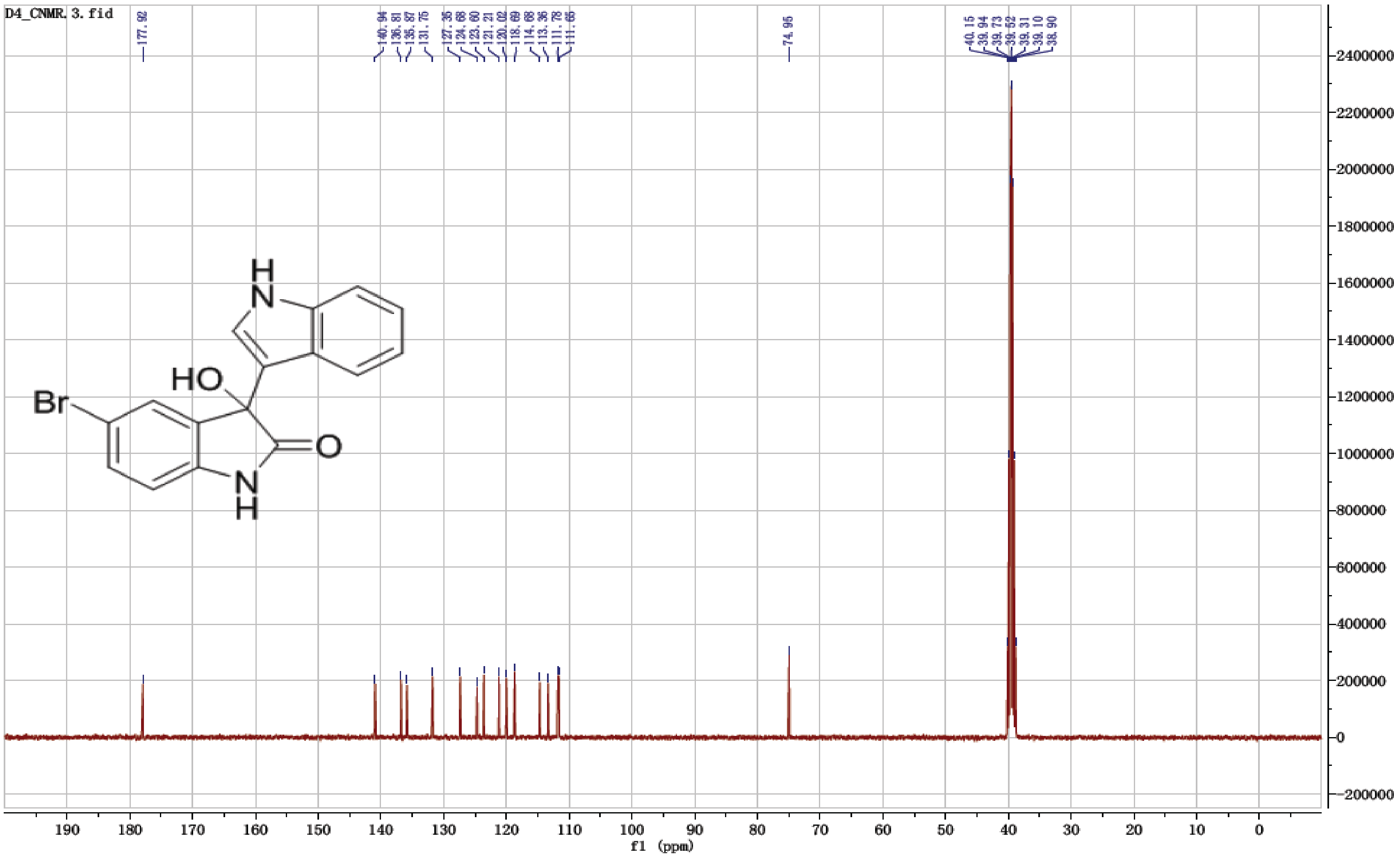
D4_CNMR.3.fid
177.92
140.94
136.81
135.87
131.75
127.36
124.68
123.60
121.21
120.02
118.69
114.68
113.36
111.78
111.65
74.95
40.15
39.94
39.73
39.52
39.31
39.10
38.90
HO
Br
N
H
O
f1 (ppm)

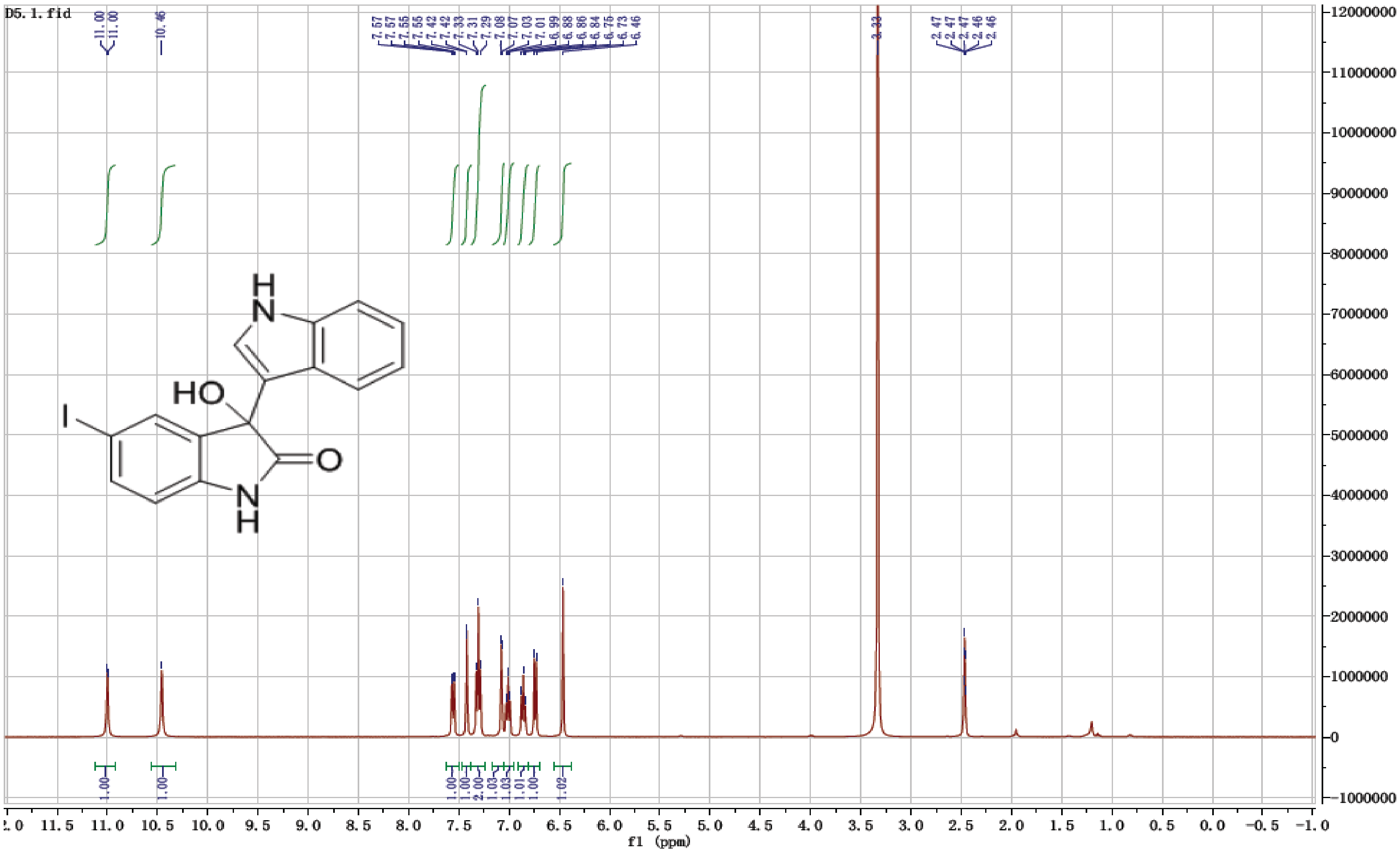

D5.1.fid
11.00
11.00
10.46
7.57
7.57
7.55
7.55
7.42
7.42
7.33
7.31
7.29
7.08
7.07
7.03
7.01
6.99
6.88
6.86
6.84
6.76
6.73
6.46
3.33
2.47
2.47
2.47
2.46
2.46
HO
I
N
H
O
f1 (ppm)

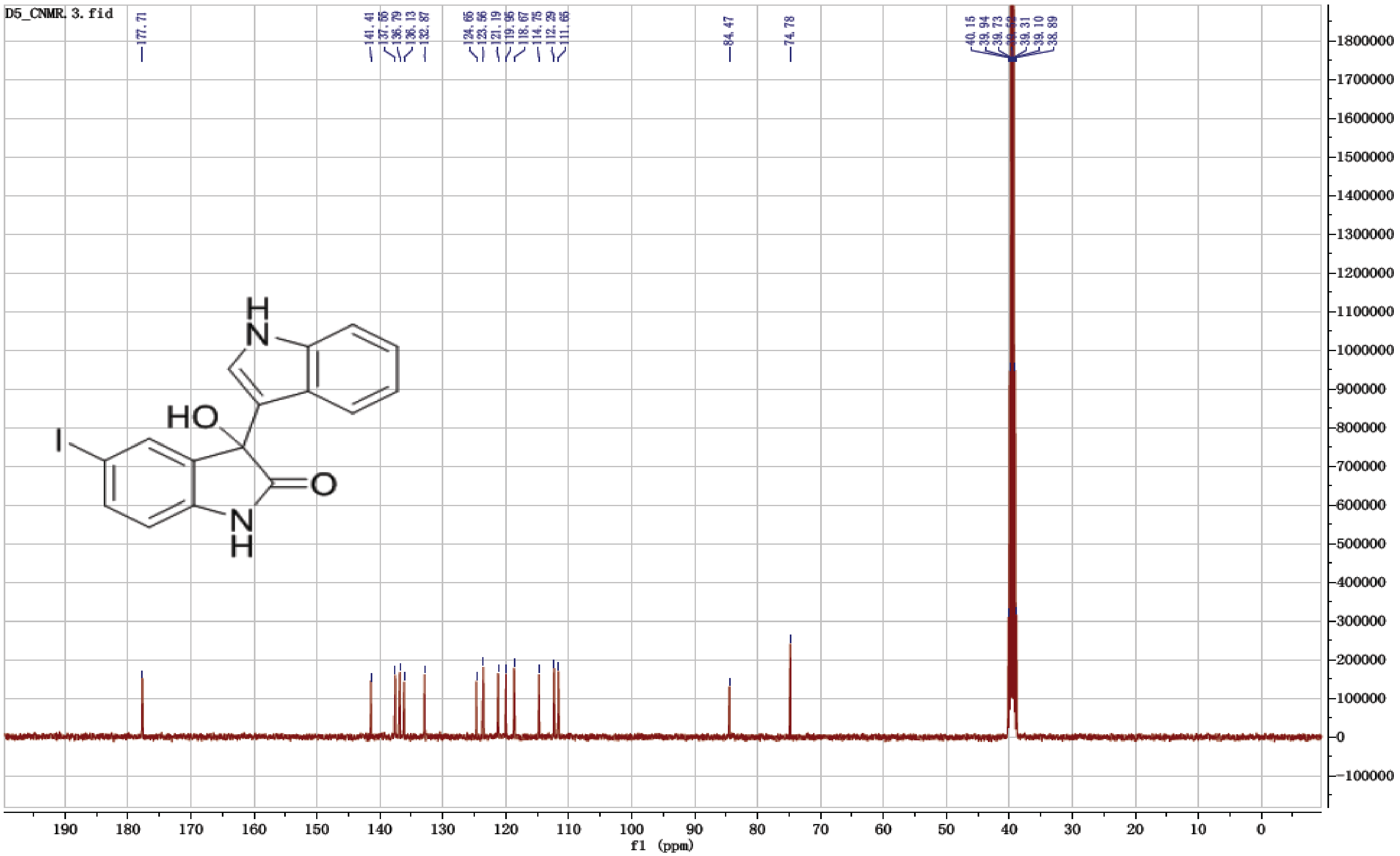

D5_CNMR.3.fid
177.71
141.41
137.65
136.79
136.13
132.87
124.65
123.56
121.19
119.95
118.67
114.75
112.29
111.65
84.47
74.78
40.15
39.94
39.73
39.52
39.31
39.10
38.89
f1 (ppm)
HO
I
NH
O
H
N

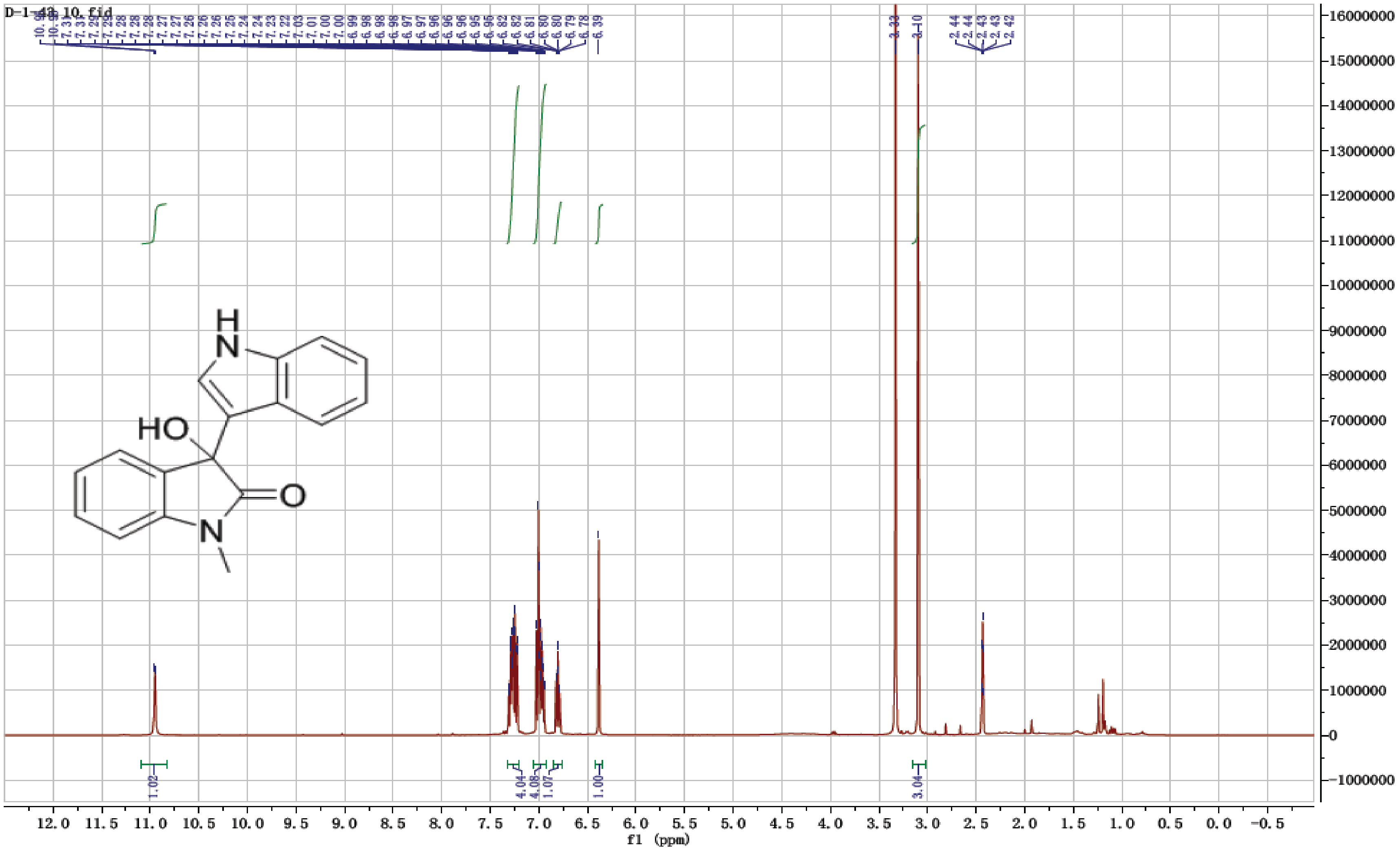
D-1-48.10.fid
HO
N
H
O
f1 (ppm)
1.02
4.04
4.08
1.07
1.00
3.04

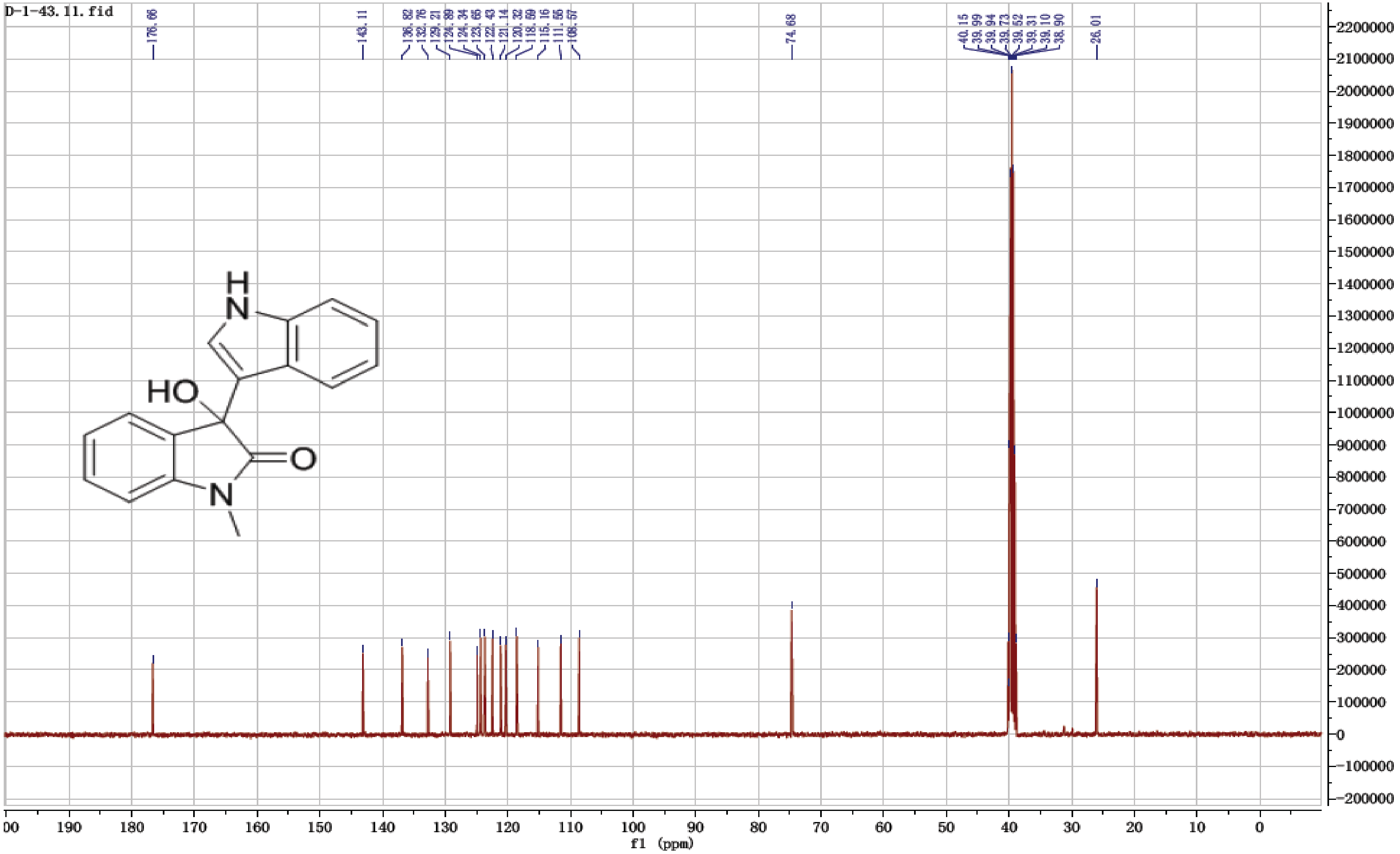
D-1-43.11.fid
176.66
143.11
136.82
132.76
129.21
124.89
124.34
123.65
122.43
121.14
120.32
118.59
115.16
111.65
108.57
74.68
40.15
39.99
39.94
39.73
39.52
39.31
39.10
38.90
26.01
HO
H
N
N
O
f1 (ppm)

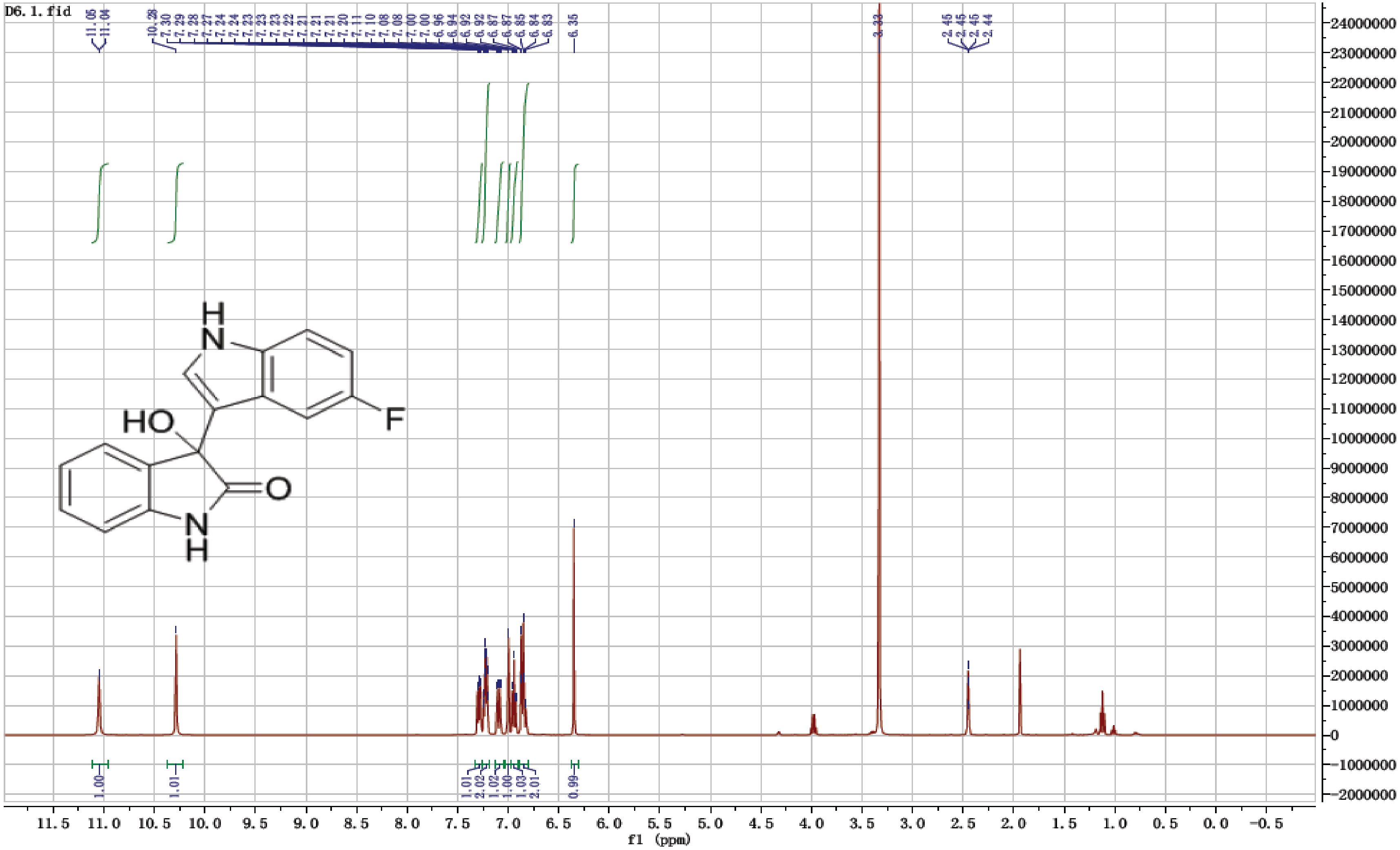
D6.1.fid
HO
F
f1 (ppm)

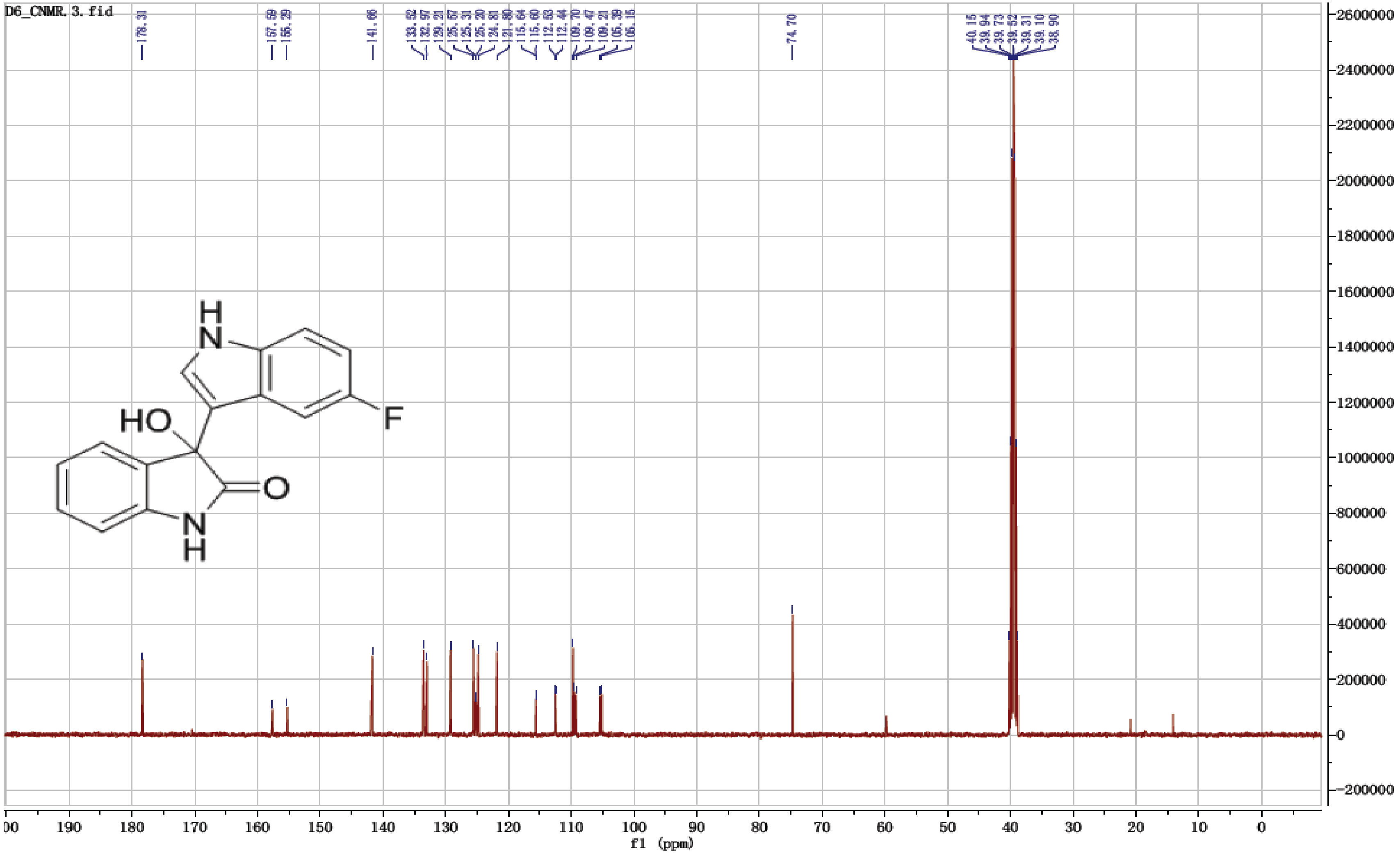
D6_CNMR.3.fid
178.31
157.59
155.29
141.66
133.52
132.97
129.21
125.57
125.31
125.20
124.81
121.80
115.64
115.60
112.53
112.44
109.70
109.47
109.21
105.39
105.15
74.70
40.15
39.94
39.73
39.52
39.31
39.10
38.90
HO
H
N
N
H
O
F
f1 (ppm)

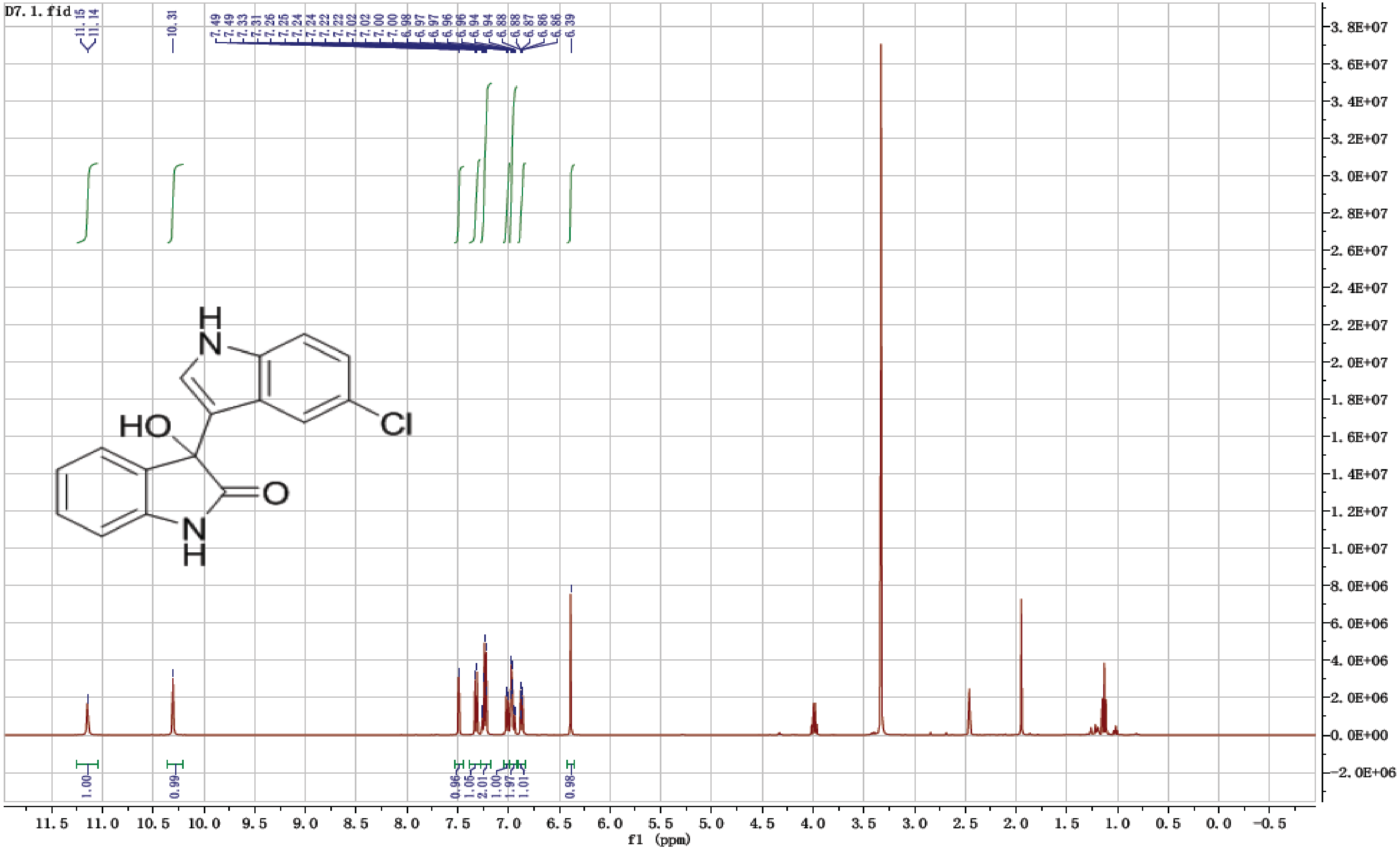
D7.1.fid
11.15
11.14
10.31
7.49
7.49
7.33
7.31
7.26
7.25
7.24
7.24
7.22
7.22
7.22
7.02
7.02
7.00
7.00
6.98
6.97
6.97
6.96
6.96
6.94
6.94
6.88
6.88
6.87
6.86
6.86
6.39
1.00
0.99
0.96
1.05
2.01
1.00
1.97
1.01
0.98
HO
Cl
f1 (ppm)

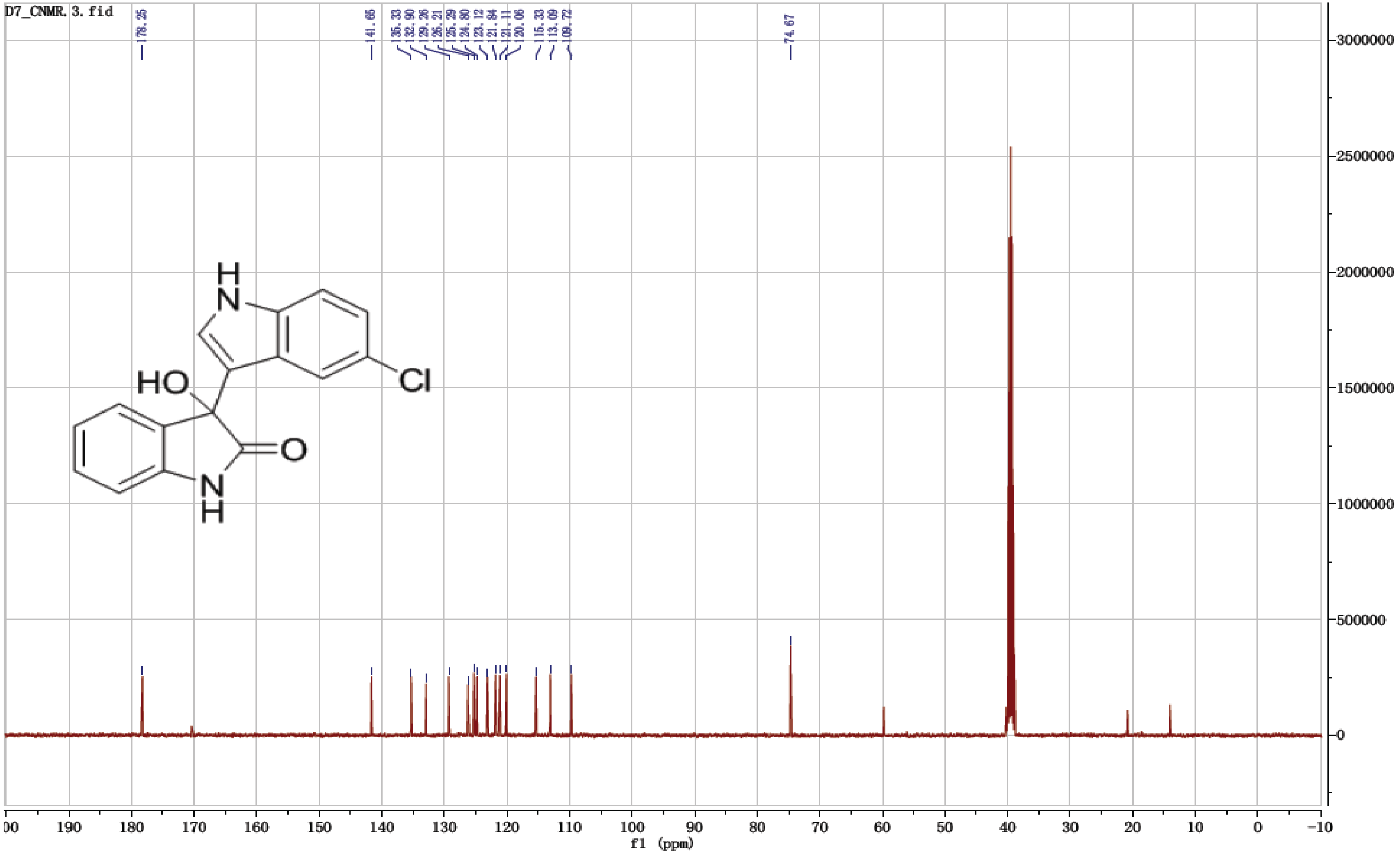
D7_CNMR.3.fid
178.25
141.65
135.33
132.90
129.26
126.21
125.29
124.80
123.12
121.84
121.11
120.06
115.33
113.09
109.72
74.67
HO
Cl
O
N
H
f1 (ppm)

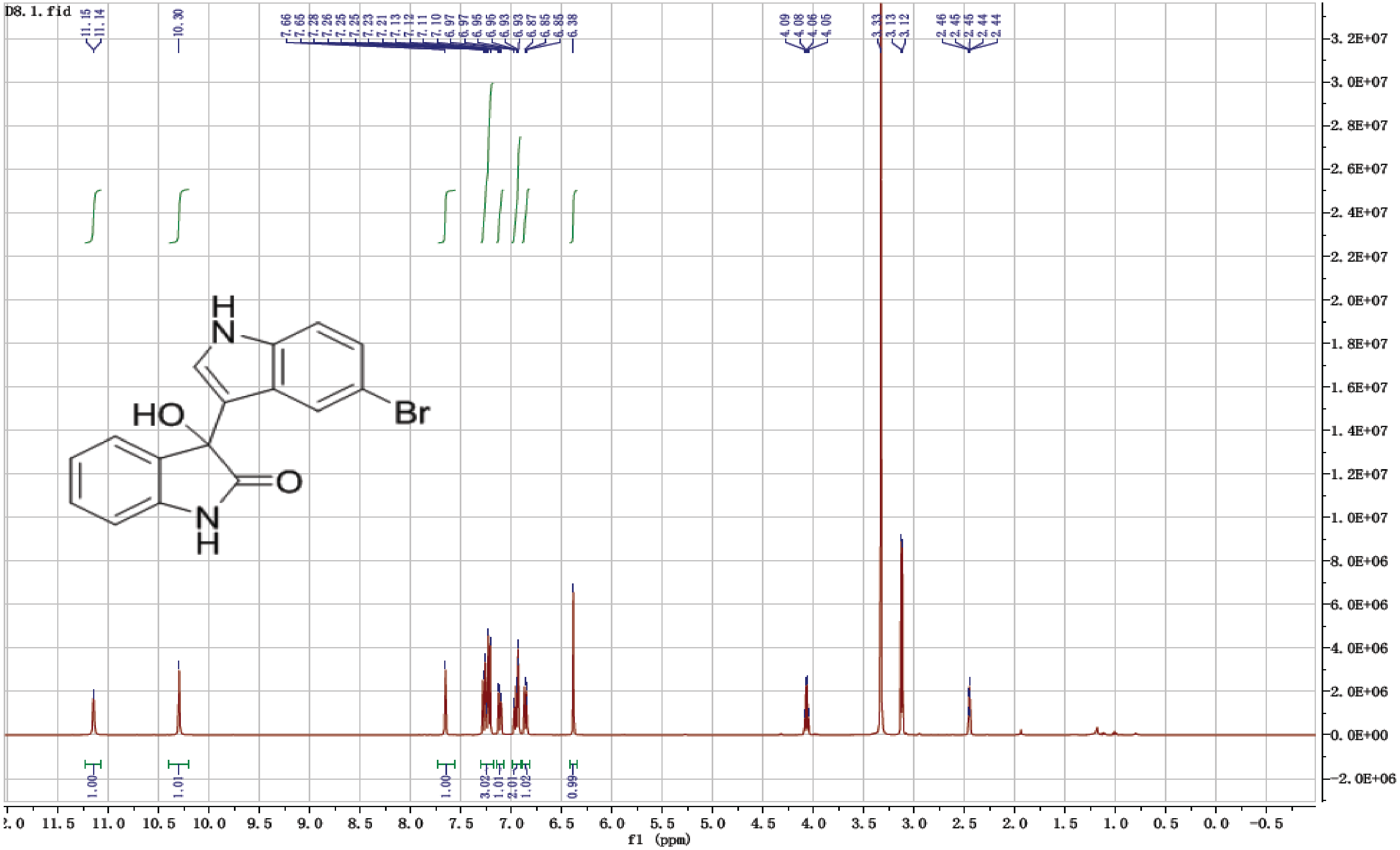
D8.1.fid
11.15
11.14
10.30
7.66
7.65
7.28
7.26
7.25
7.25
7.23
7.21
7.13
7.12
7.11
7.10
6.97
6.97
6.95
6.95
6.93
6.93
6.87
6.85
6.85
6.38
4.09
4.08
4.06
4.05
3.33
3.13
3.12
2.46
2.45
2.45
2.44
2.44
HO
Br
O
1.00
1.01
1.00
3.02
1.01
2.01
1.02
0.99
f1 (ppm)

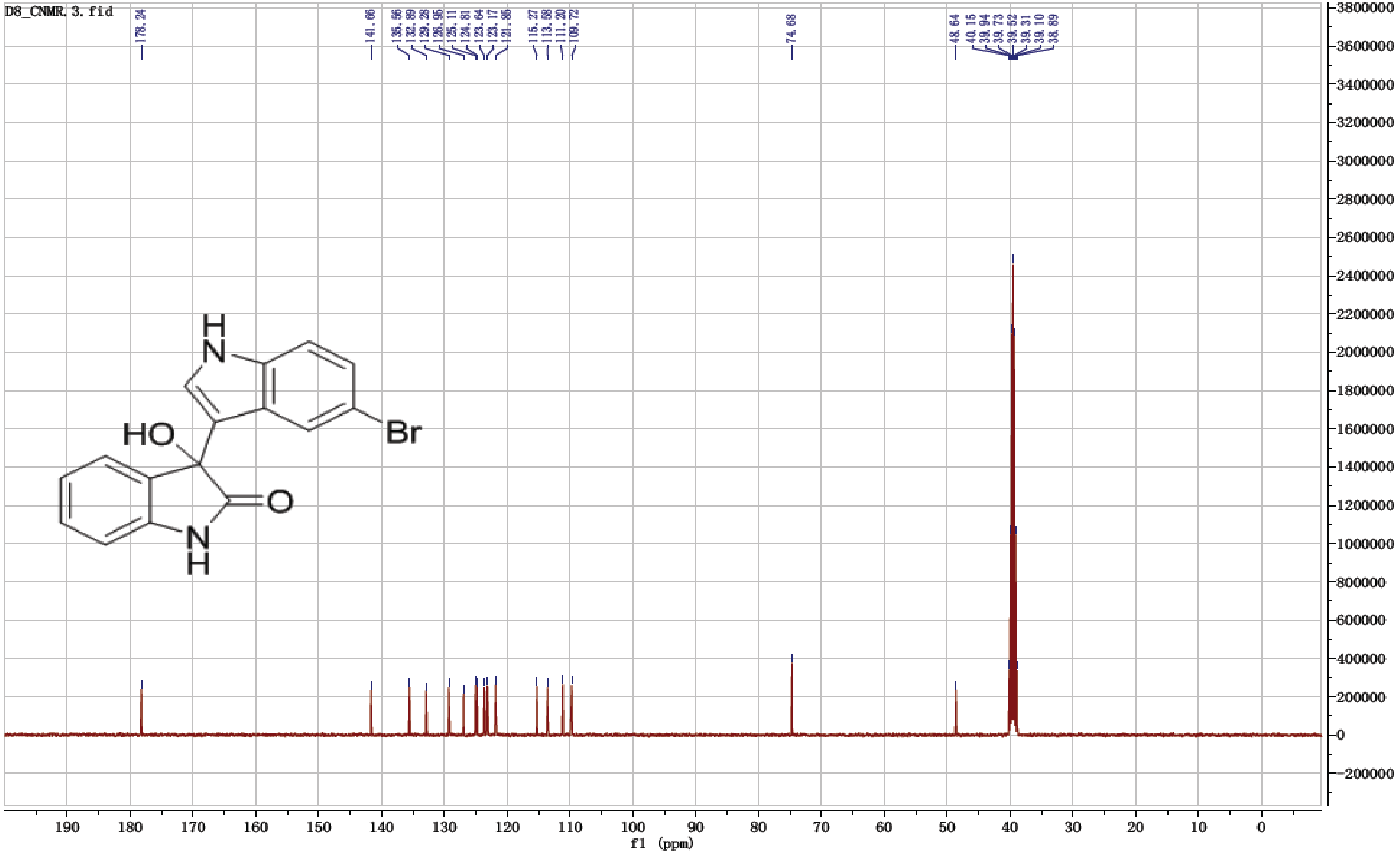

D8_CNMR.3.fid
178.24
141.66
135.56
132.89
129.28
126.95
125.11
124.81
123.64
123.17
121.86
115.27
113.58
111.20
109.72
74.68
48.64
40.15
39.94
39.73
39.52
39.31
39.10
38.89
HO
H
N
Br
N
H
O
f1 (ppm)

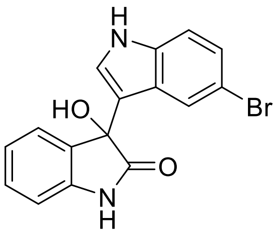
H
N
HO
Br
O
N
H

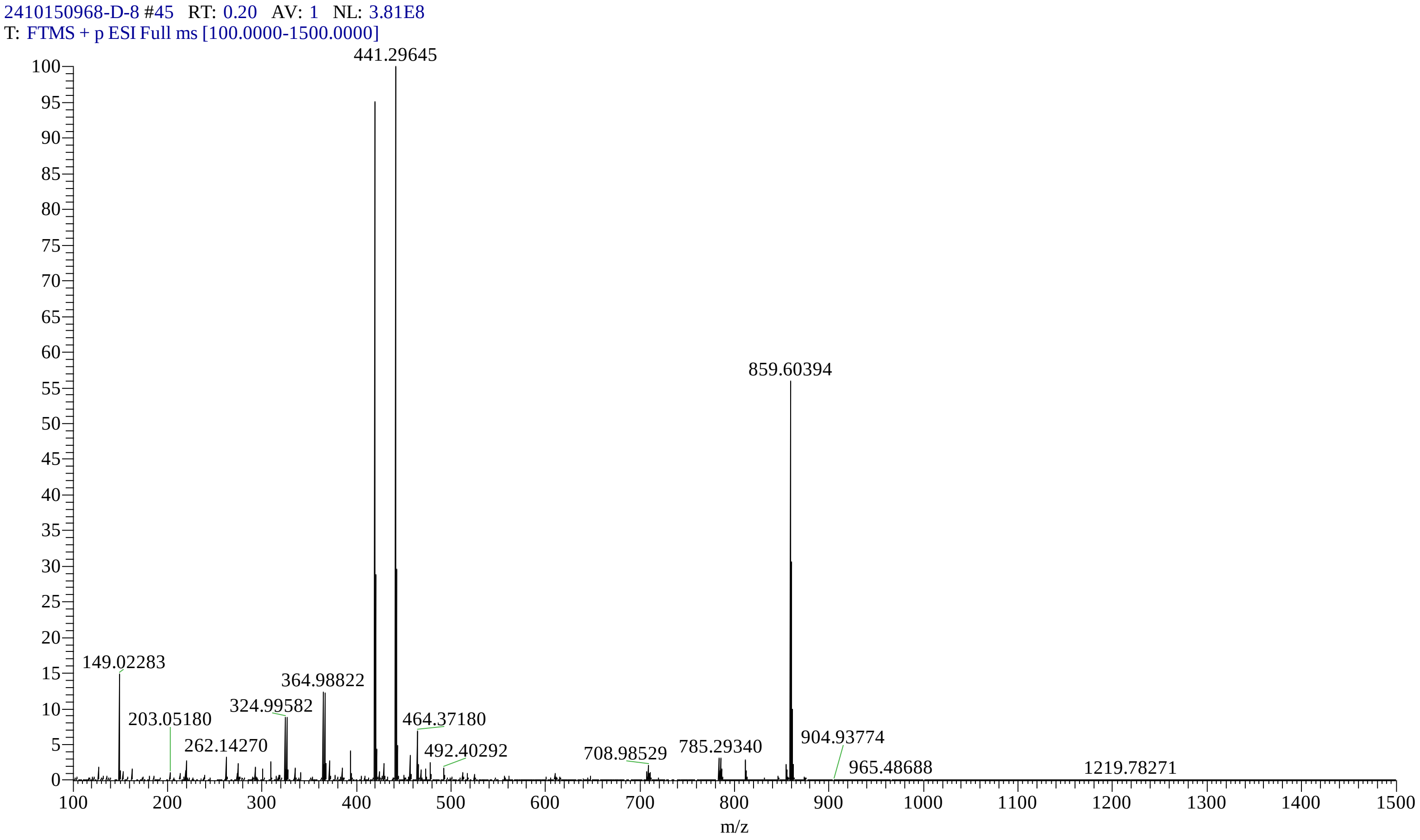
2410150968-D-8 #45 RT: 0.20 AV: 1 NL: 3.81E8
T: FTMS + p ESI Full ms [100.0000-1500.0000]
441.29645
859.60394
149.02283
364.98822
324.99582
203.05180
464.37180
262.14270
492.40292
708.98529
785.29340
904.93774
965.48688
1219.78271
m/z

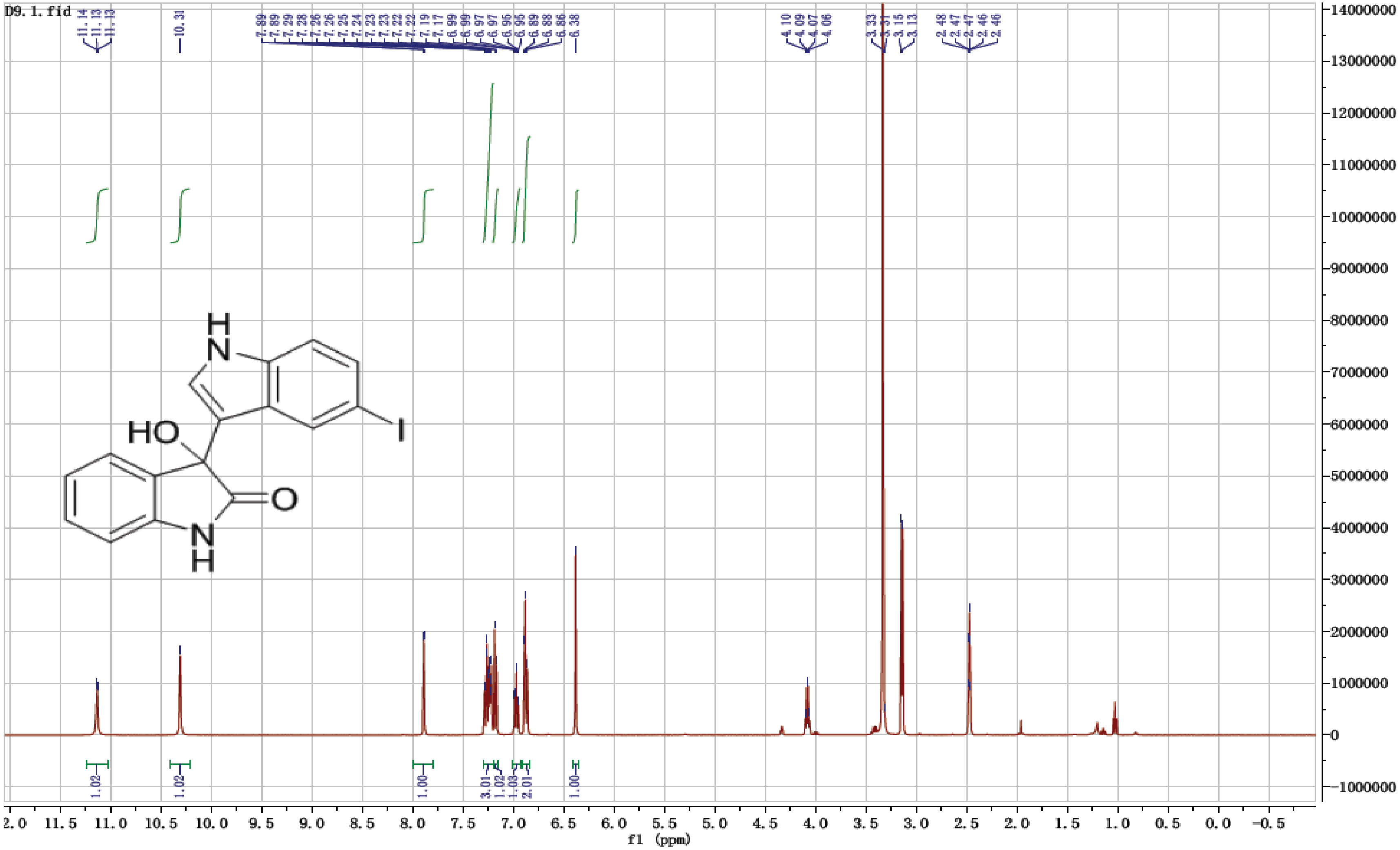
D9.1.fid
11.14
11.13
11.13
10.31
7.89
7.89
7.29
7.28
7.26
7.26
7.25
7.24
7.23
7.23
7.22
7.22
7.19
7.17
6.99
6.99
6.97
6.97
6.95
6.95
6.89
6.88
6.86
6.38
4.10
4.09
4.07
4.06
3.33
3.31
3.15
3.13
2.48
2.47
2.47
2.46
2.46
1.02
1.02
1.00
3.01
1.02
1.03
2.01
1.00
f1 (ppm)
HO
H
N
I
O
N
H

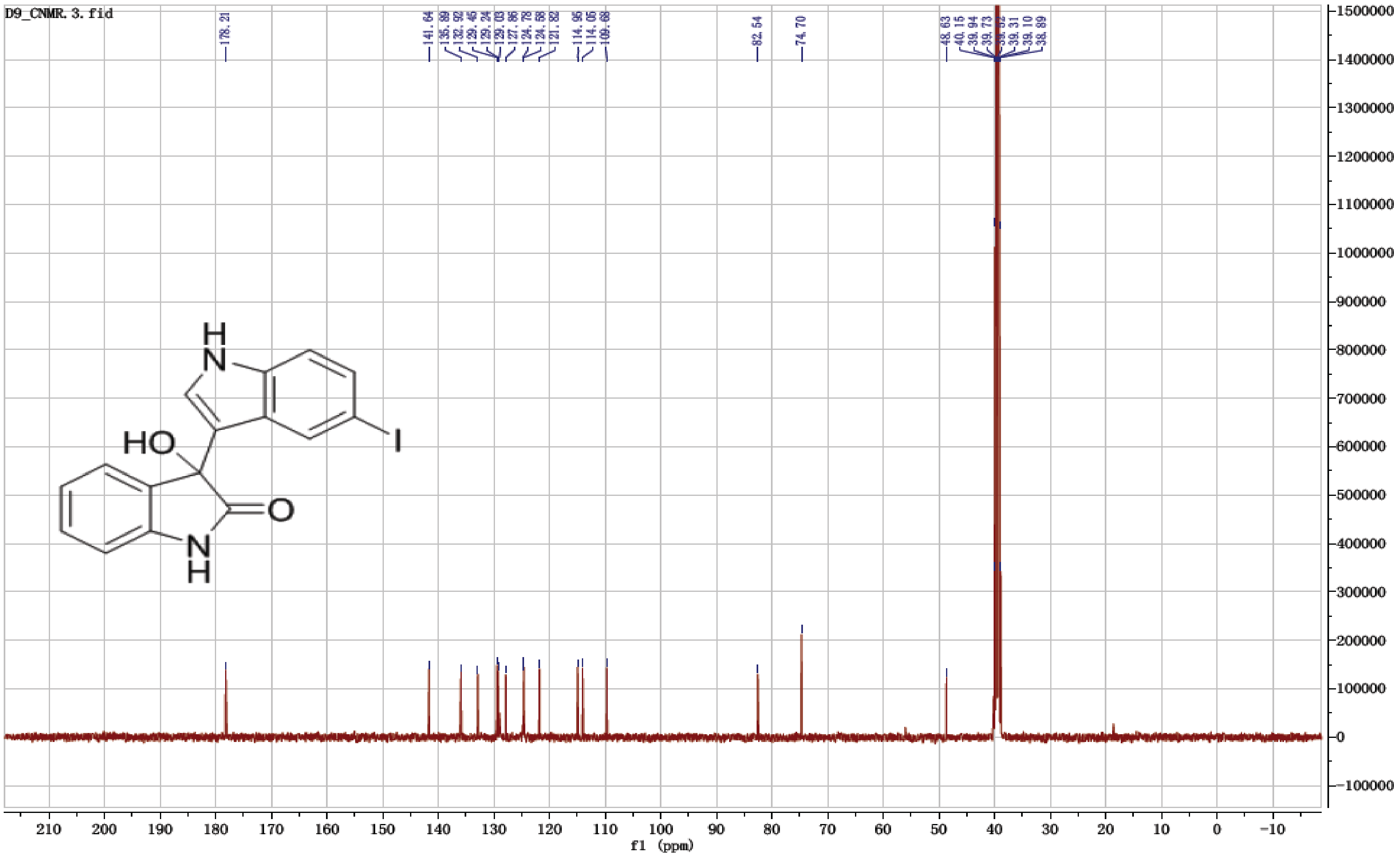
D9_CNMR.3.fid
178.21
141.64
135.89
132.92
129.45
129.24
129.03
127.86
124.78
124.58
121.82
114.95
114.05
109.68
82.54
74.70
48.63
40.15
39.94
39.73
39.31
39.10
38.89
HO
H
N
N
H
O
I
f1 (ppm)

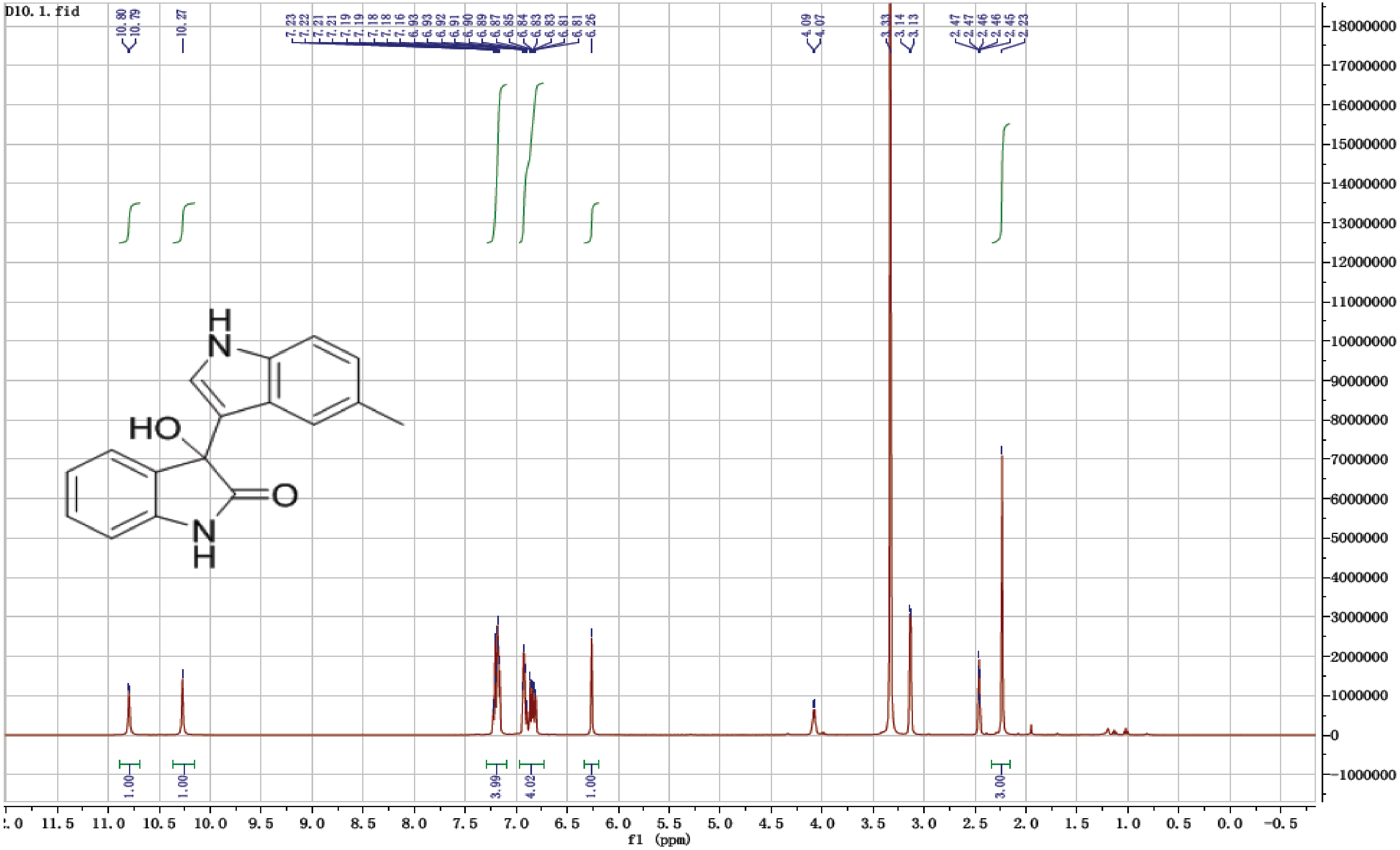

D10. 1. fid
f1 (ppm)
HO
H
N
O

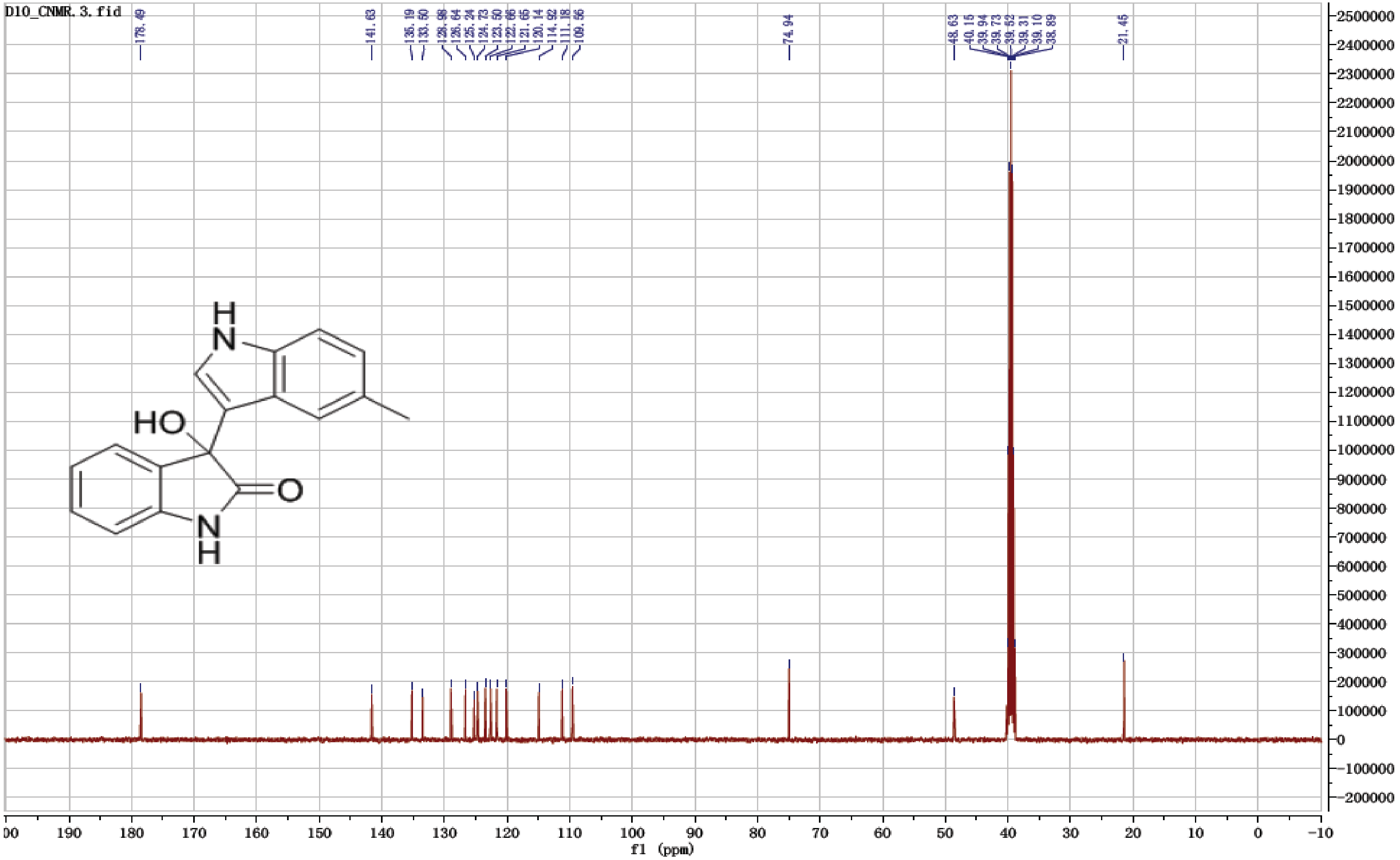

D10_CNMR.3.fid
178.49
141.63
135.19
133.50
128.98
126.64
125.24
124.73
123.50
122.66
121.65
120.14
114.92
111.18
109.56
74.94
48.63
40.15
39.94
39.73
39.52
39.31
39.10
38.89
21.45
f1 (ppm)
HO
NH

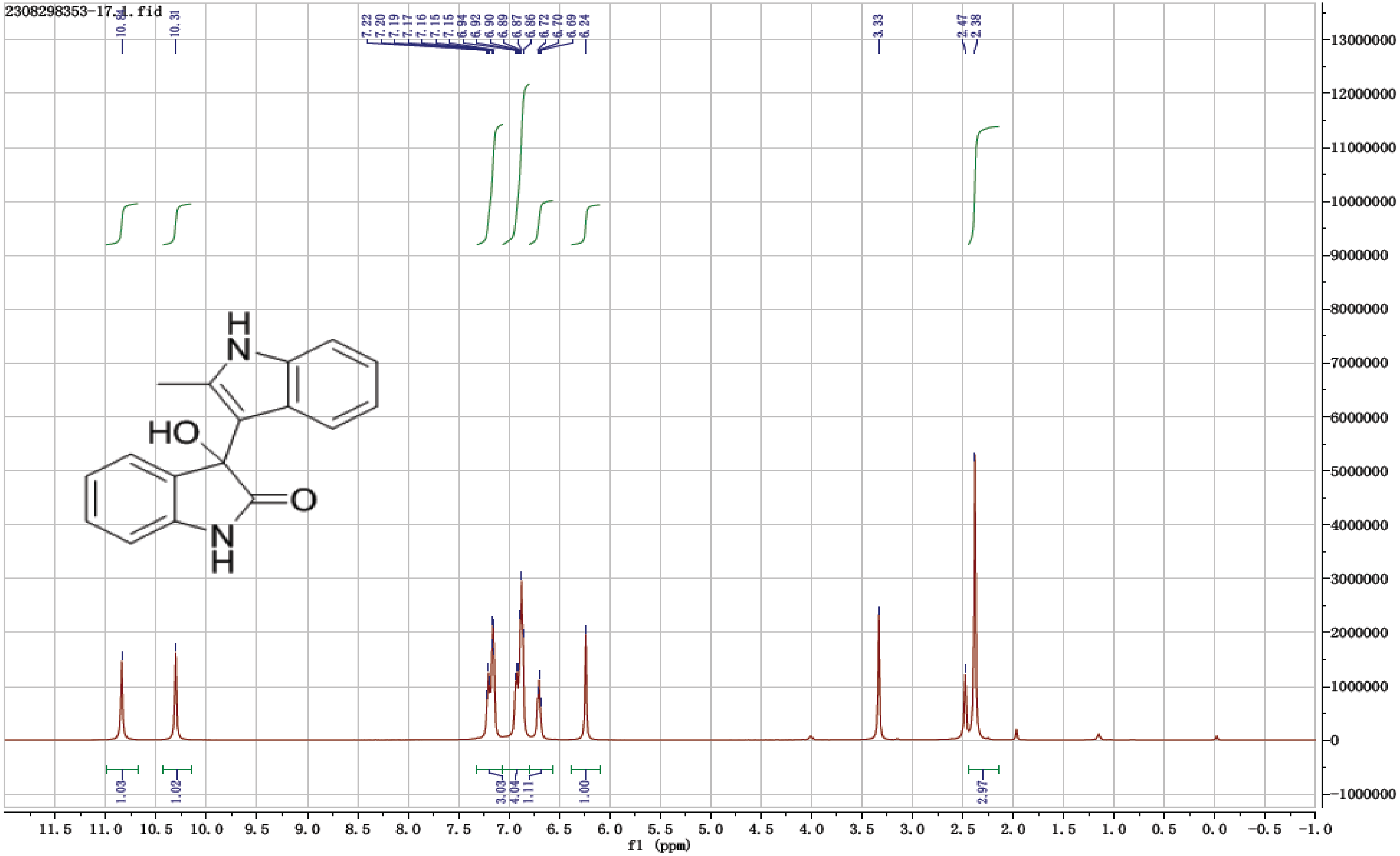

2308298353-17.1.fid
10.84
10.31
7.22
7.20
7.19
7.17
7.16
7.15
7.15
6.94
6.92
6.90
6.89
6.87
6.86
6.72
6.70
6.69
6.24
3.33
2.47
2.38
1.03
1.02
3.03
4.04
1.11
1.00
2.97
HO
N
H
O
f1 (ppm)

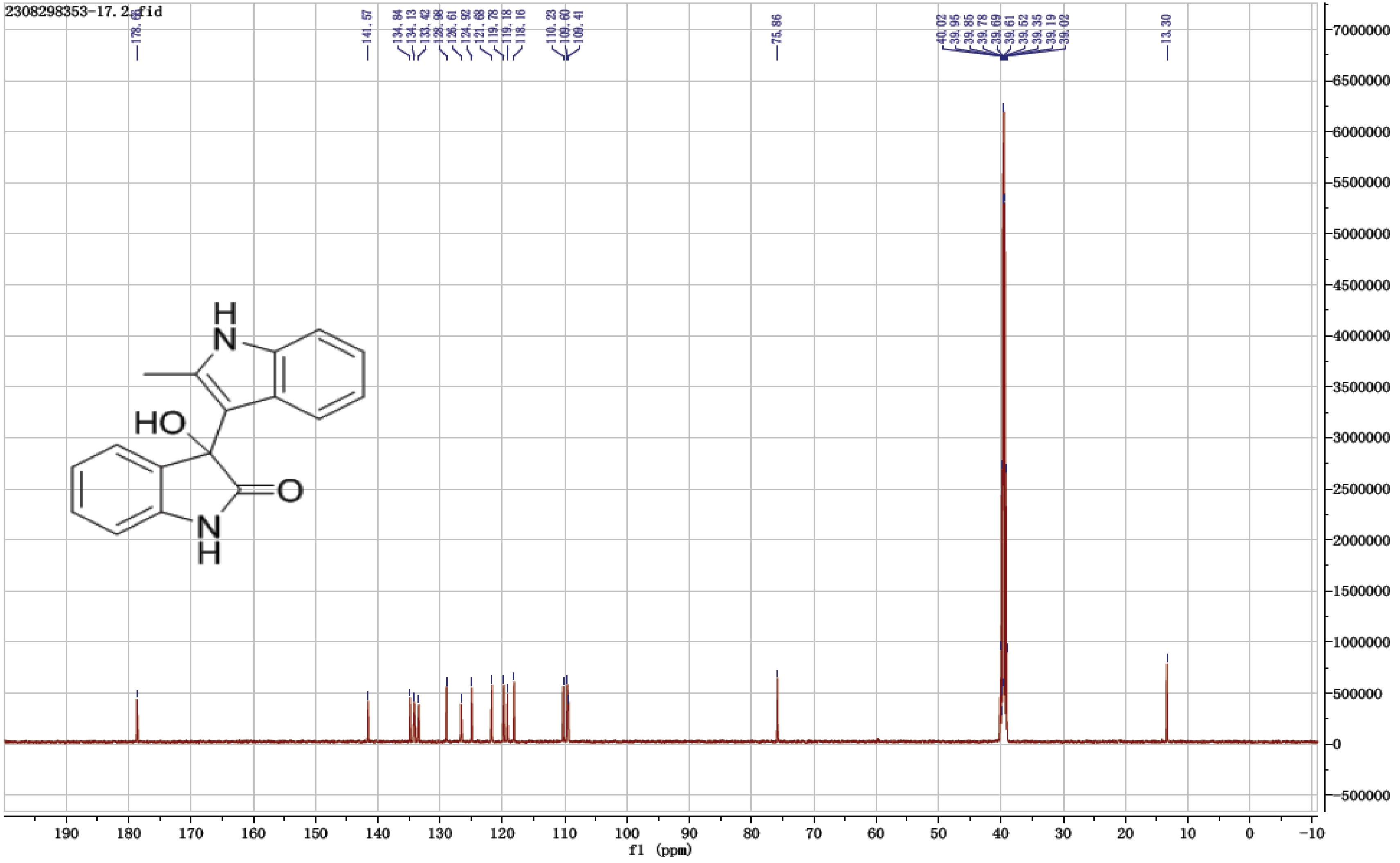
2308298353-17.2.fid
178.61
141.57
134.84
134.13
133.42
128.98
126.61
124.92
121.68
119.78
119.18
118.16
110.23
109.60
109.41
75.86
40.02
39.95
39.85
39.78
39.69
39.61
39.52
39.35
39.19
39.02
13.30
HO
N
H
O
f1 (ppm)

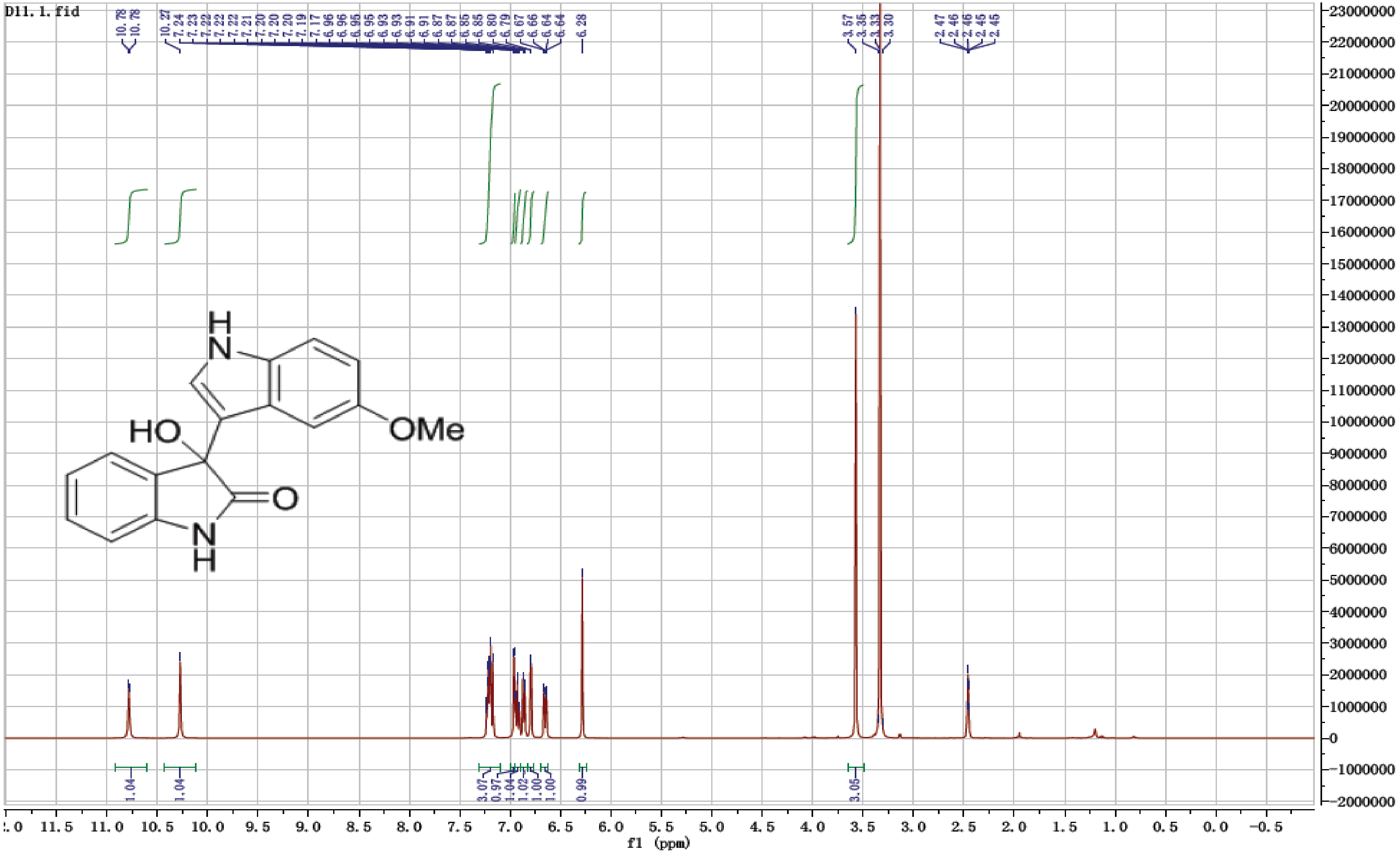
D11.1.fid
f1 (ppm)
HO
OMe
O
N
H

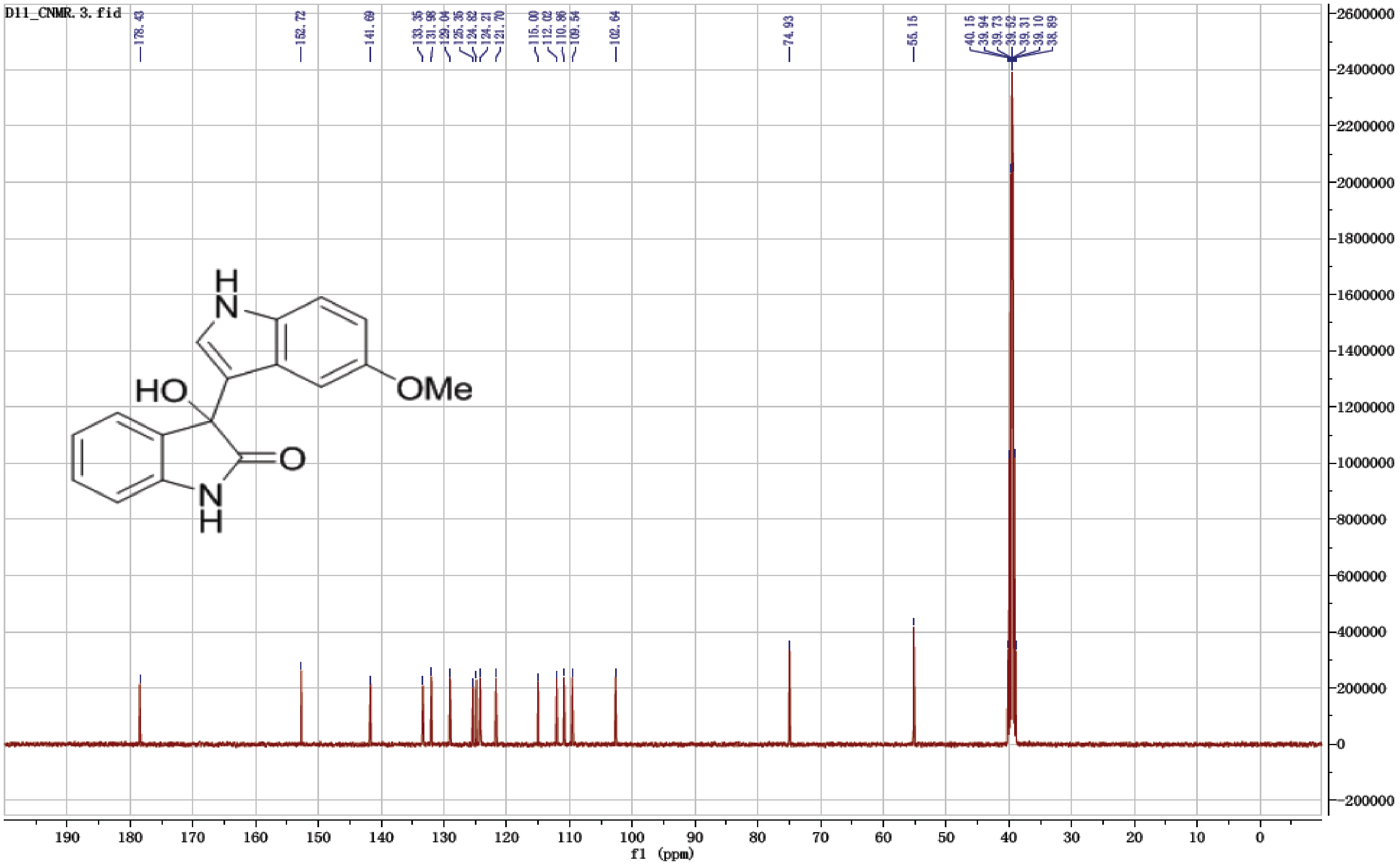

D11_CNMR.3.fid
178.43
152.72
141.69
133.35
131.98
129.04
125.35
124.82
124.21
121.70
115.00
112.02
110.86
109.54
102.64
74.93
55.15
40.15
39.94
39.73
39.52
39.31
39.10
38.89
HO
H
N
OMe
O
N
H
f1 (ppm)

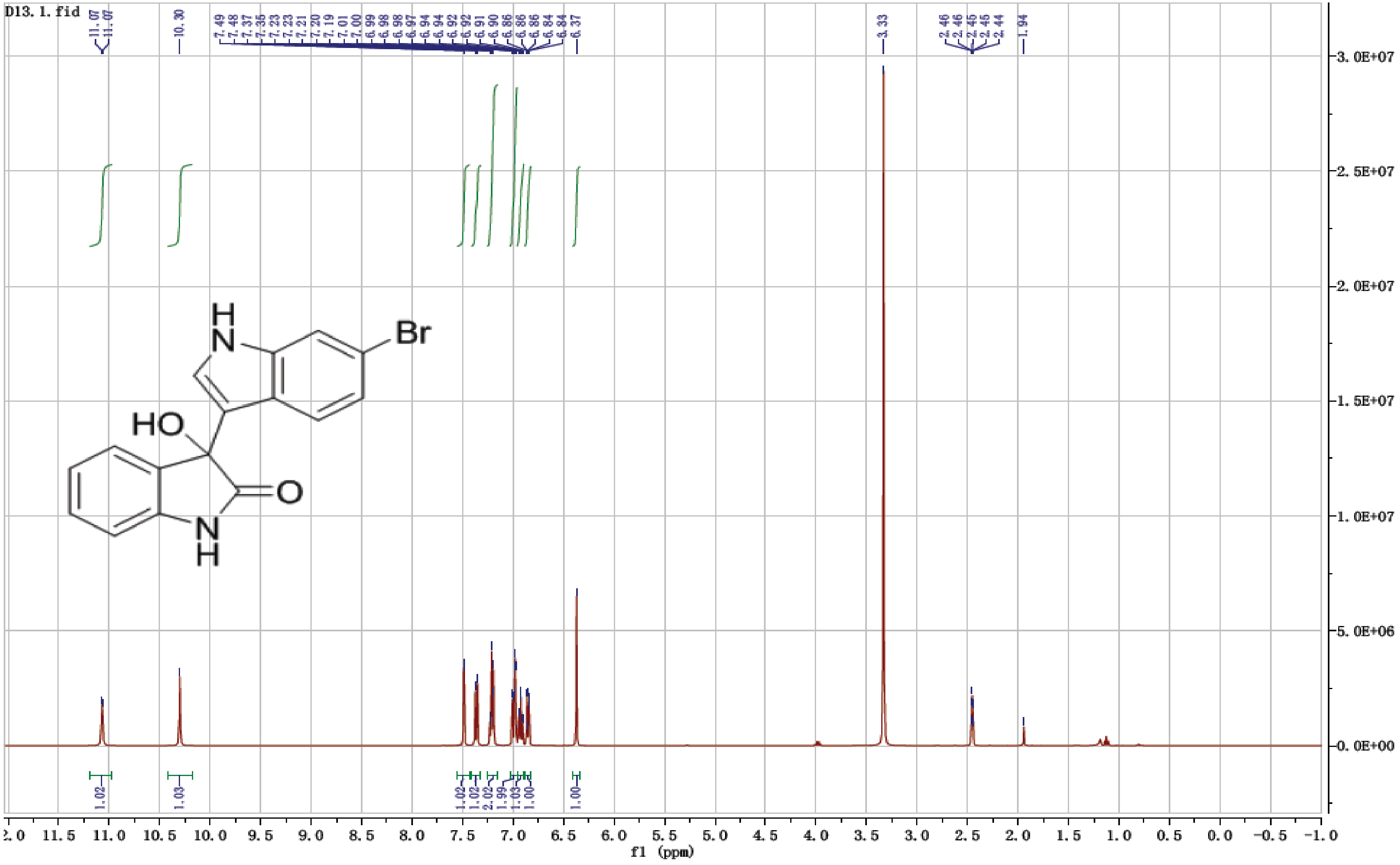
D13.1.fid
11.07
10.30
3.33
1.94
1.02
1.03
1.00
f1 (ppm)
HO
Br
N
H
O

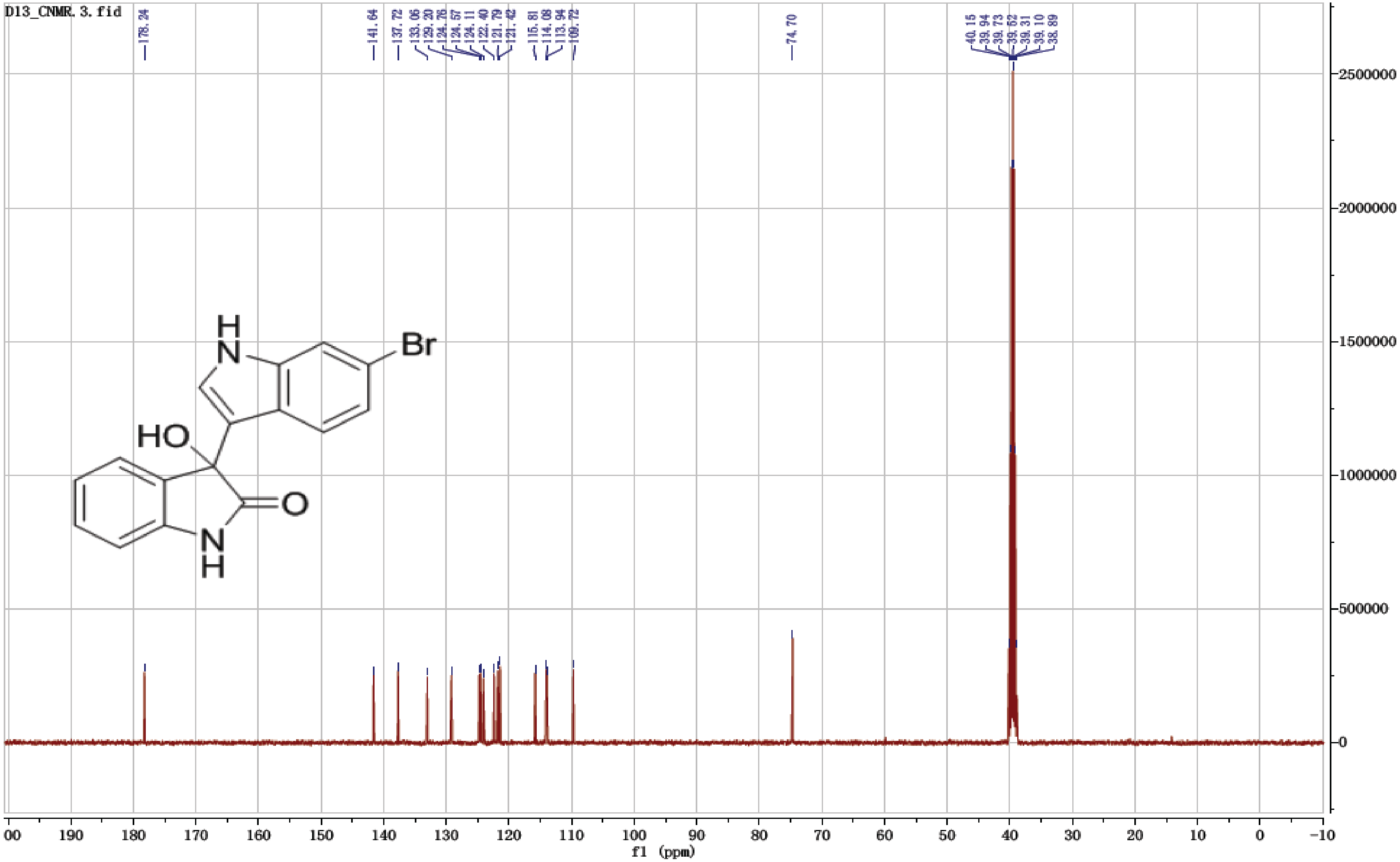

D13_CNMR.3.fid
178.24
141.64
137.72
133.06
129.20
124.76
124.57
124.11
122.40
121.79
121.42
115.81
114.08
113.94
109.72
74.70
40.15
39.94
39.73
39.52
39.31
39.10
38.89
HO
N
H
O
N
H
Br
f1 (ppm)

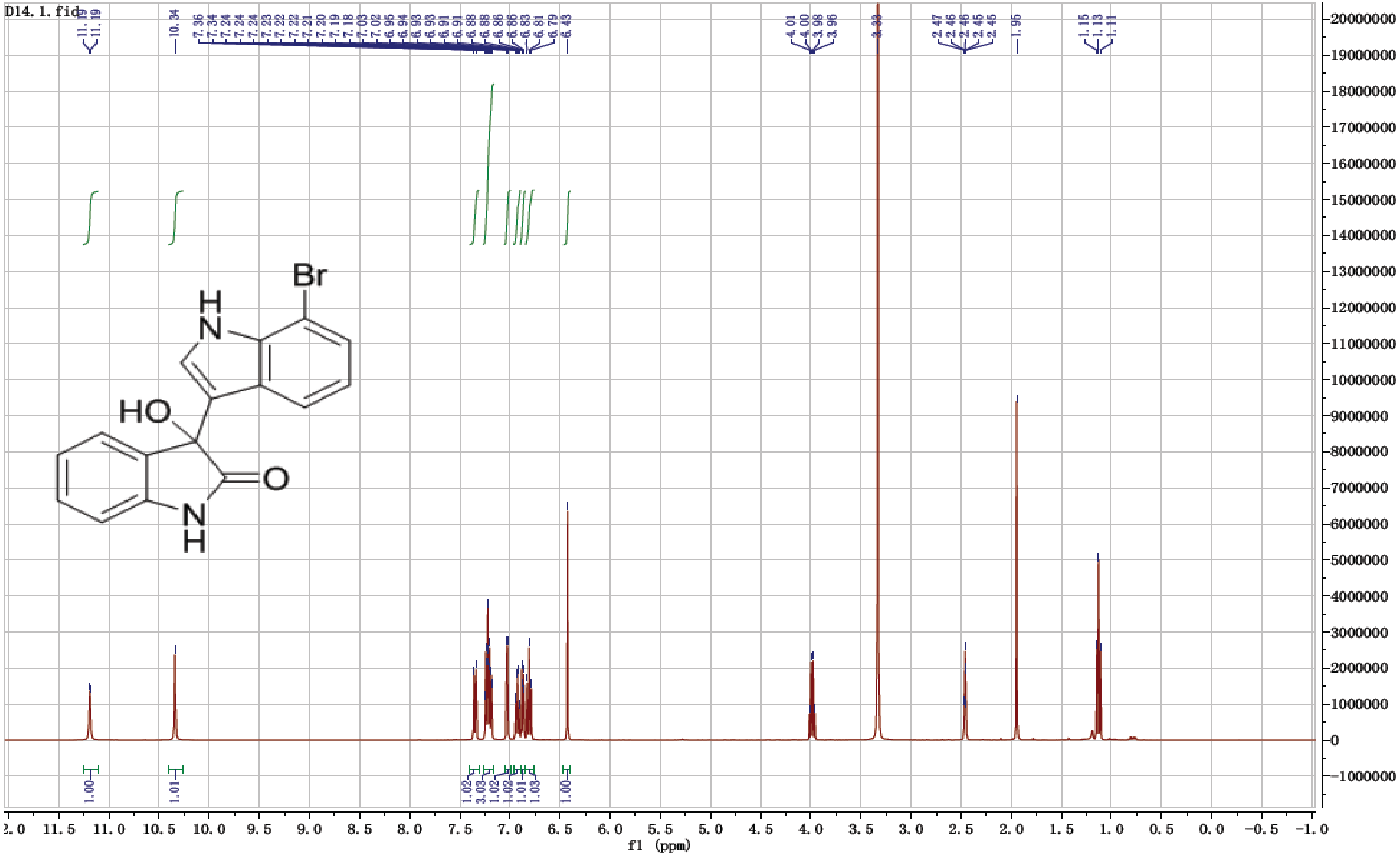
D14.1.fid
Br
HO
O
N
H
f1 (ppm)

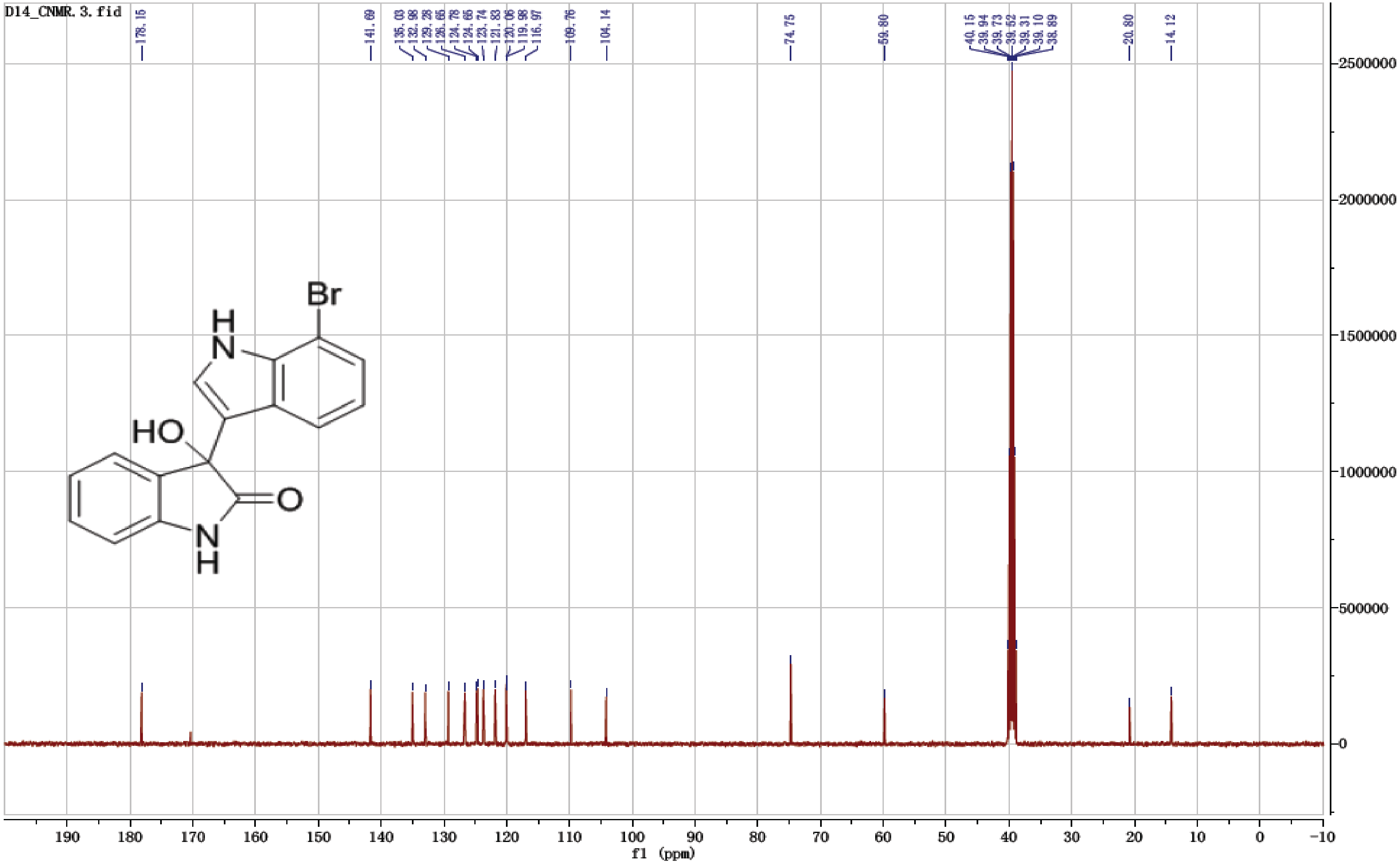
D14_CNMR.3.fid
178.15
141.69
135.03
132.98
129.28
126.65
124.78
124.65
123.74
121.83
120.06
119.98
116.97
109.76
104.14
74.75
59.80
40.15
39.94
39.73
39.52
39.31
39.10
38.89
20.80
14.12
f1 (ppm)
Br
HO
O
N
H

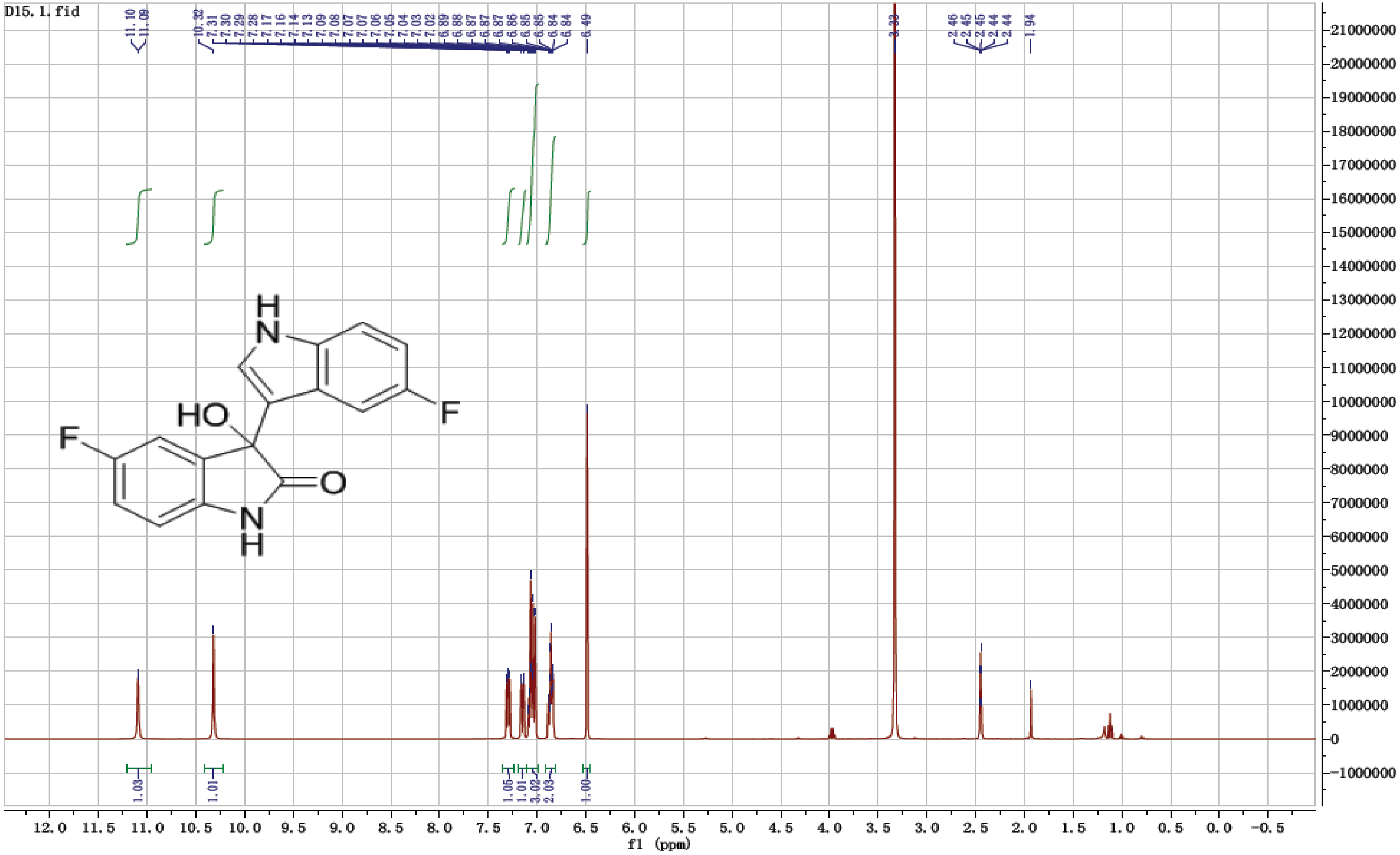
D15.1.fid
f1 (ppm)

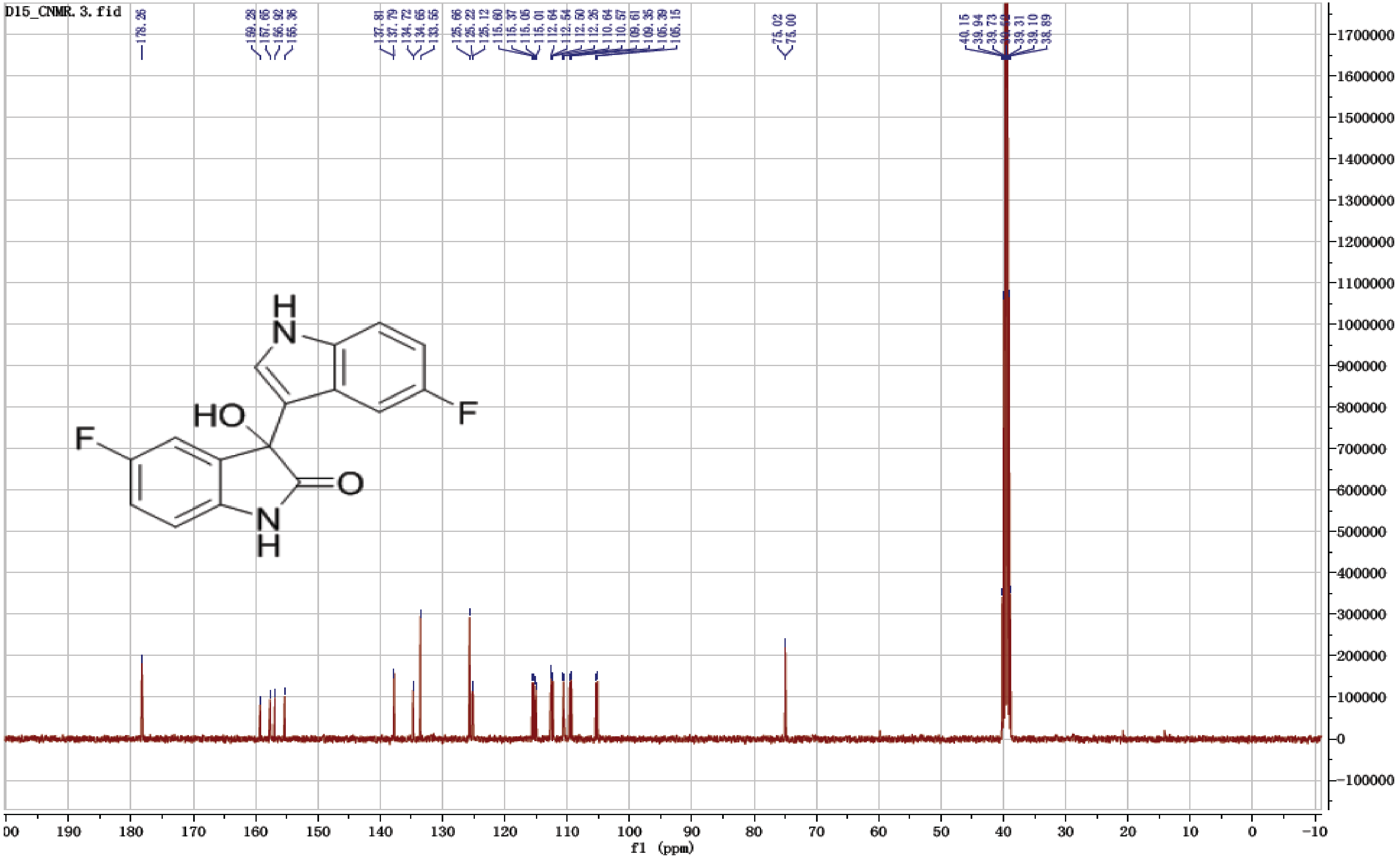
D15_CNMR.3.fid
f1 (ppm)
HO
F
F
N
H
O

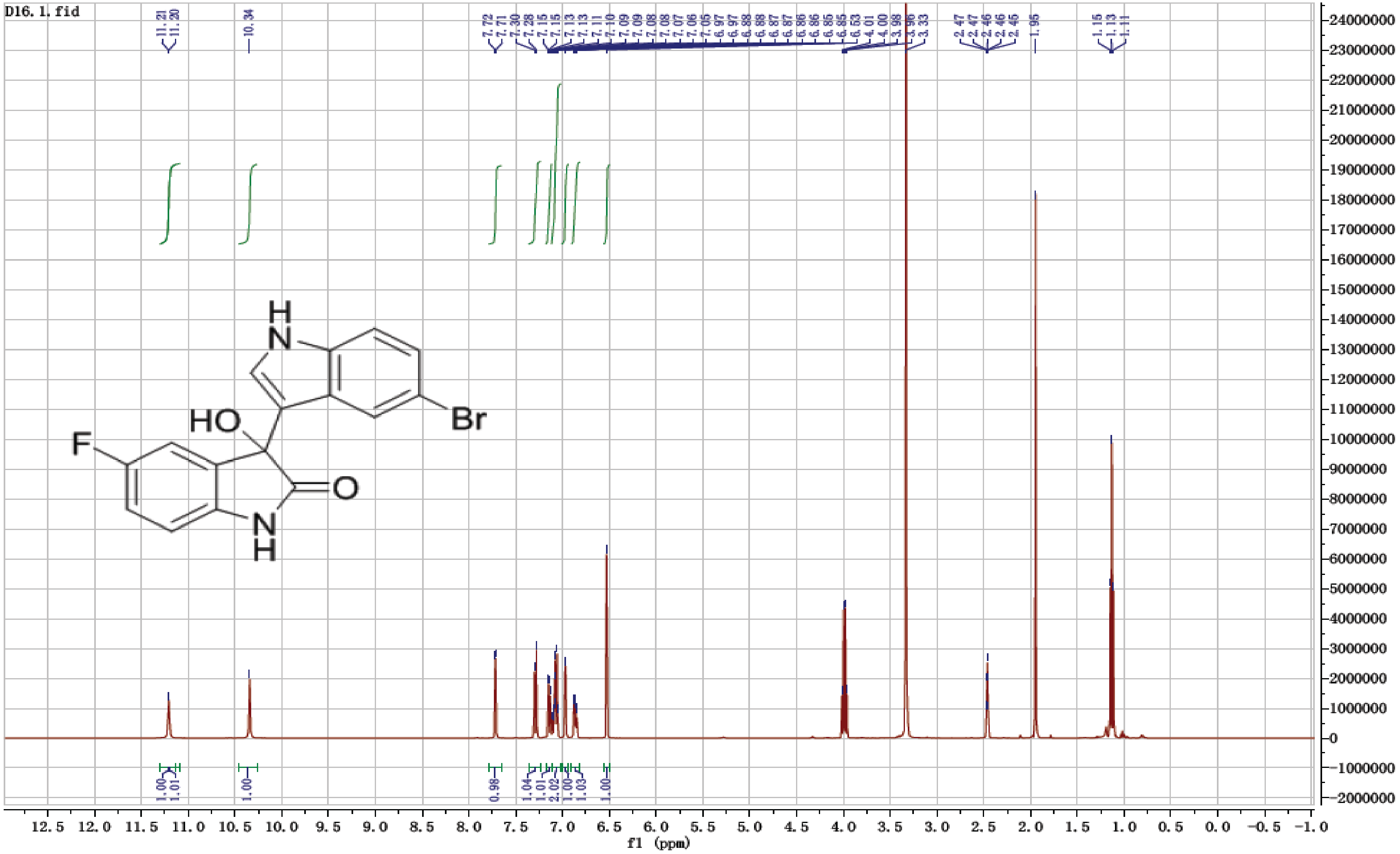

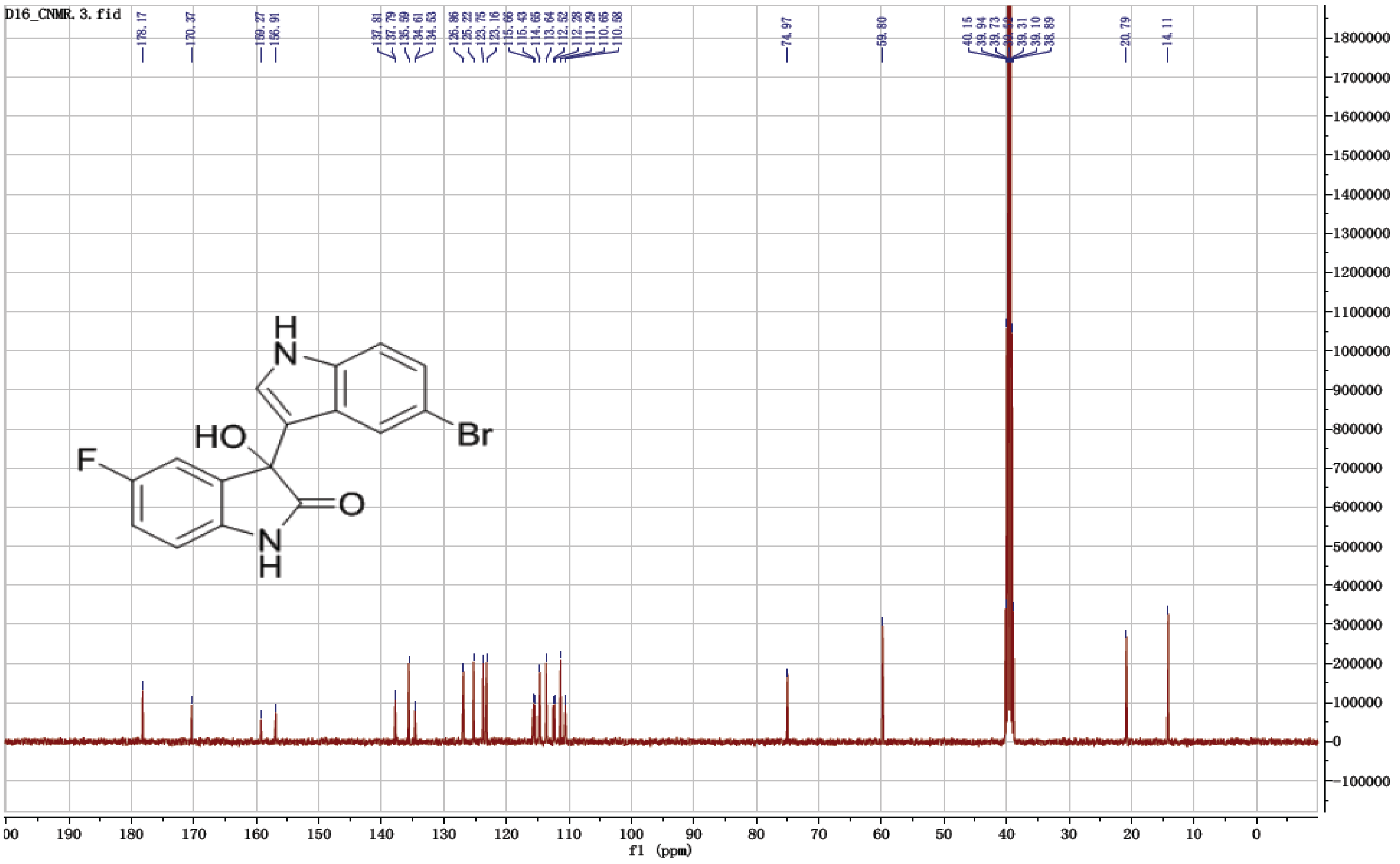
D16_CNMR.3.fid
178.17
170.37
159.27
156.91
137.81
137.79
135.59
134.61
134.53
126.86
125.22
123.75
123.16
115.66
115.43
114.65
113.64
112.62
112.28
111.29
110.65
110.58
74.97
59.80
40.15
39.94
39.73
39.31
39.10
38.89
20.79
14.11
HO
F
O
N
H
Br
f1 (ppm)

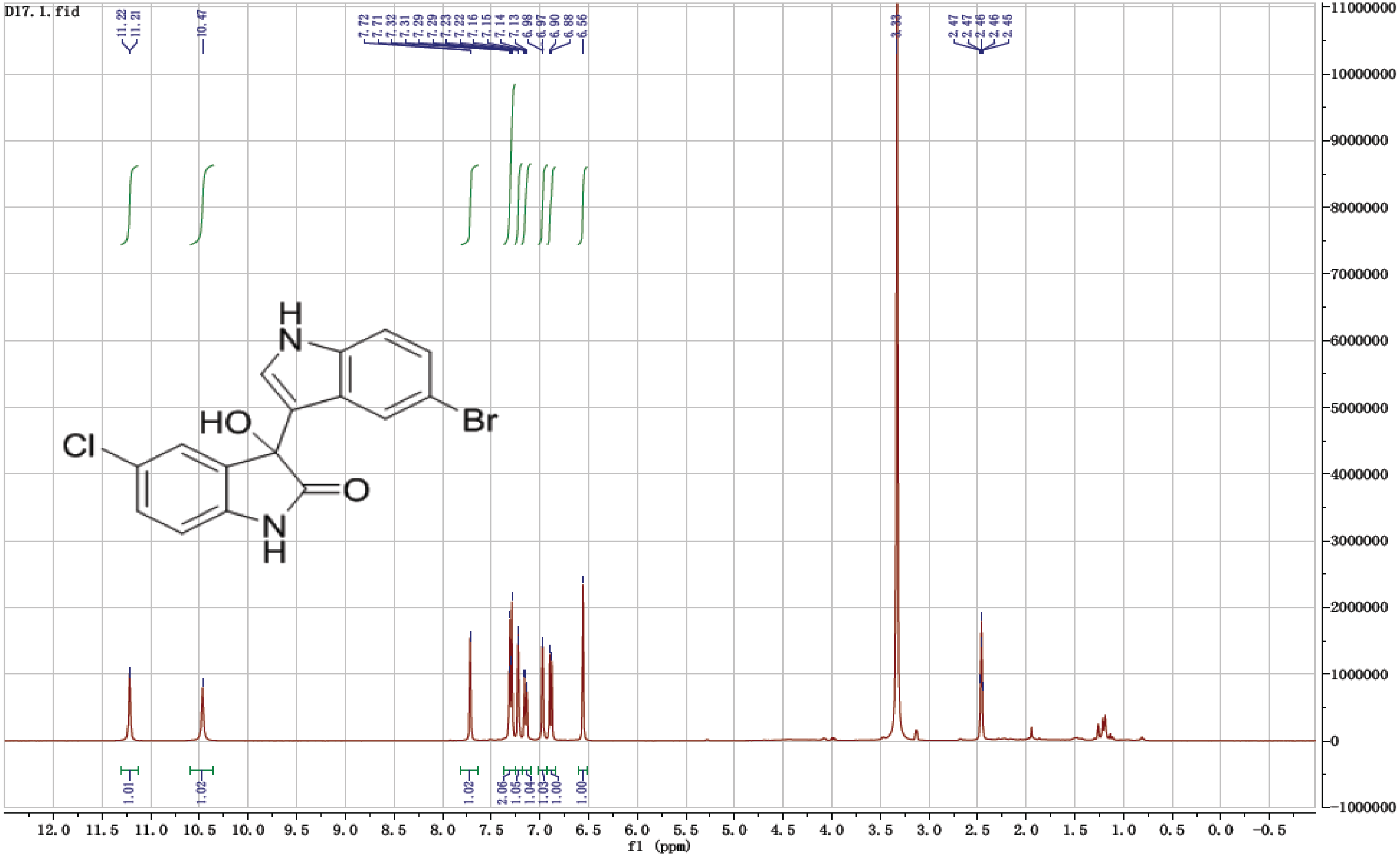
D17.1.fid
11.22
11.21
10.47
7.72
7.71
7.32
7.31
7.29
7.29
7.23
7.22
7.16
7.15
7.14
7.13
6.98
6.97
6.90
6.88
6.56
3.33
2.47
2.47
2.46
2.46
2.45
1.01
1.02
1.02
2.06
1.05
1.04
1.03
1.00
1.00
HO
Cl
Br
N
H
O
f1 (ppm)

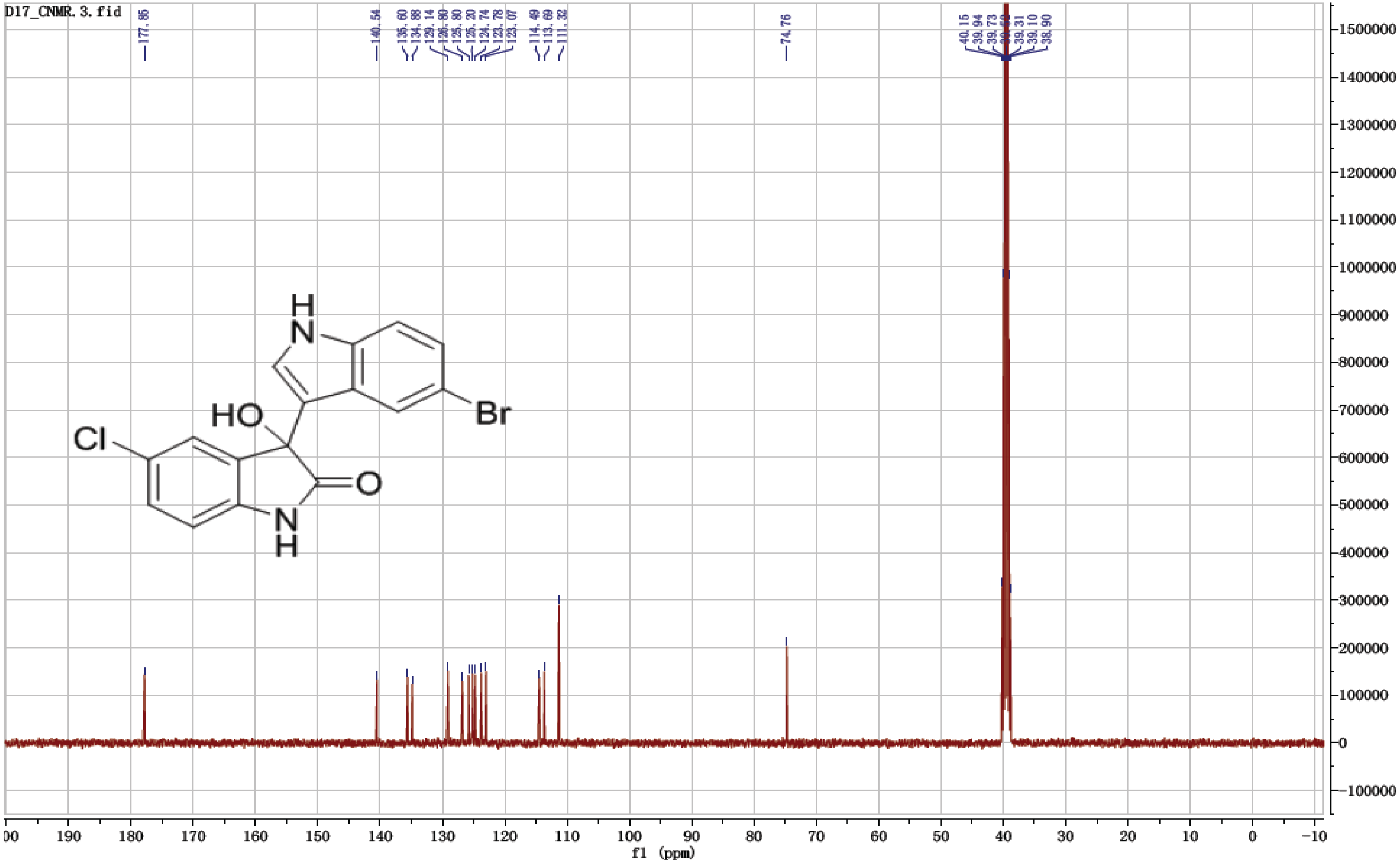

D17_CNMR.3.fid
177.85
140.54
135.60
134.88
129.14
126.80
125.80
125.20
124.74
123.78
123.07
114.49
113.69
111.32
74.76
40.15
39.94
39.73
39.31
39.10
38.90
HO
Cl
Br
O
N
H
f1 (ppm)

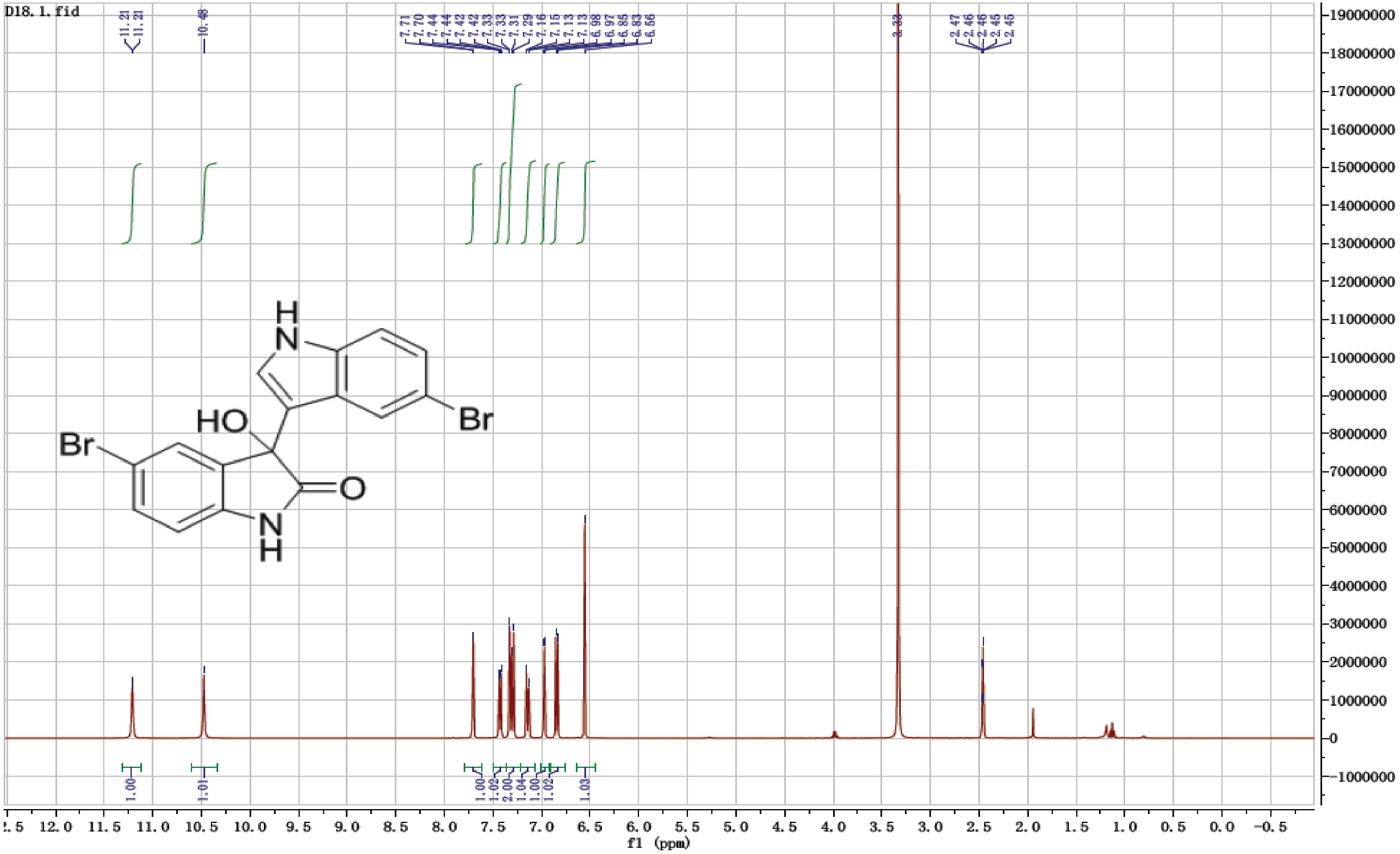

D18.1.fid
11.21
11.21
10.48
7.71
7.70
7.44
7.44
7.42
7.42
7.33
7.31
7.29
7.16
7.15
7.13
7.13
6.98
6.97
6.85
6.83
6.56
3.33
2.47
2.46
2.46
2.45
2.45
1.00
1.01
1.00
1.02
2.00
1.04
1.00
1.02
1.03
HO
Br
Br
N
H
N
H
O
f1 (ppm)

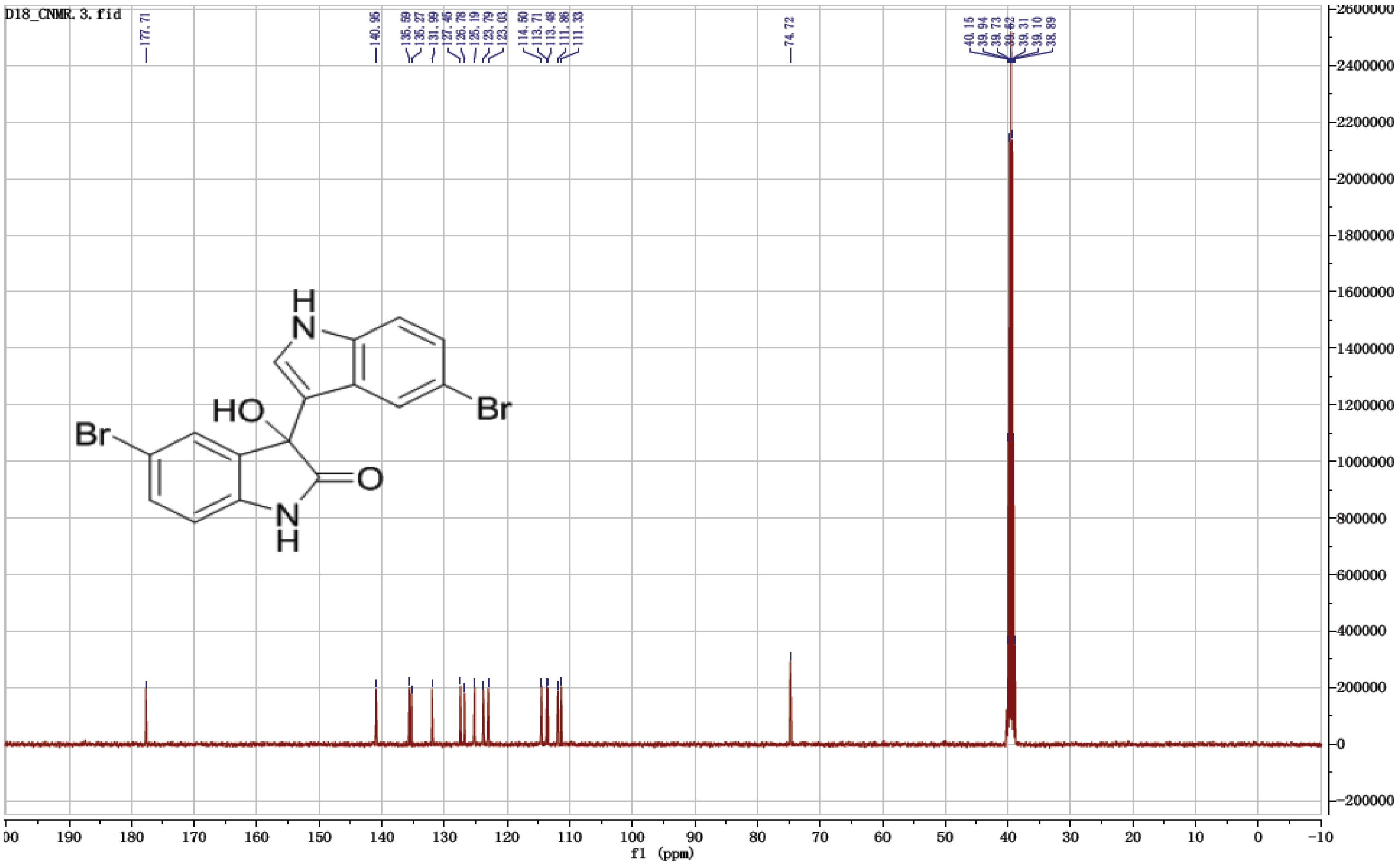
D18_CNMR.3.fid
177.71
140.95
135.59
135.27
131.99
127.40
126.78
125.19
123.79
123.03
114.50
113.71
113.48
111.86
111.33
74.72
40.15
39.94
39.73
39.52
39.31
39.10
38.89
HO
Br
N
H
O
Br
H
N
f1 (ppm)

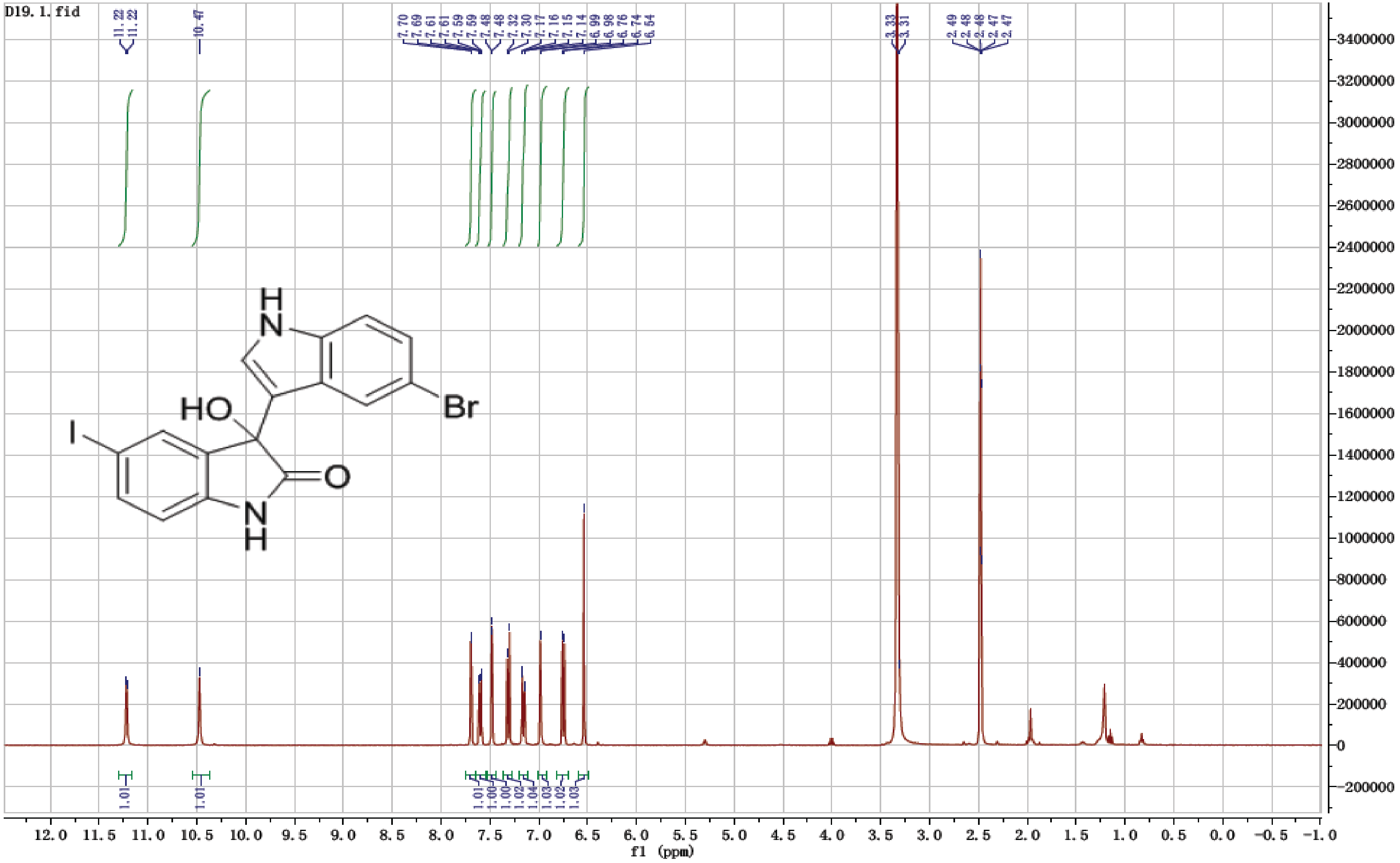
D19.1.fid
f1 (ppm)
HO
Br
I
O
N
H

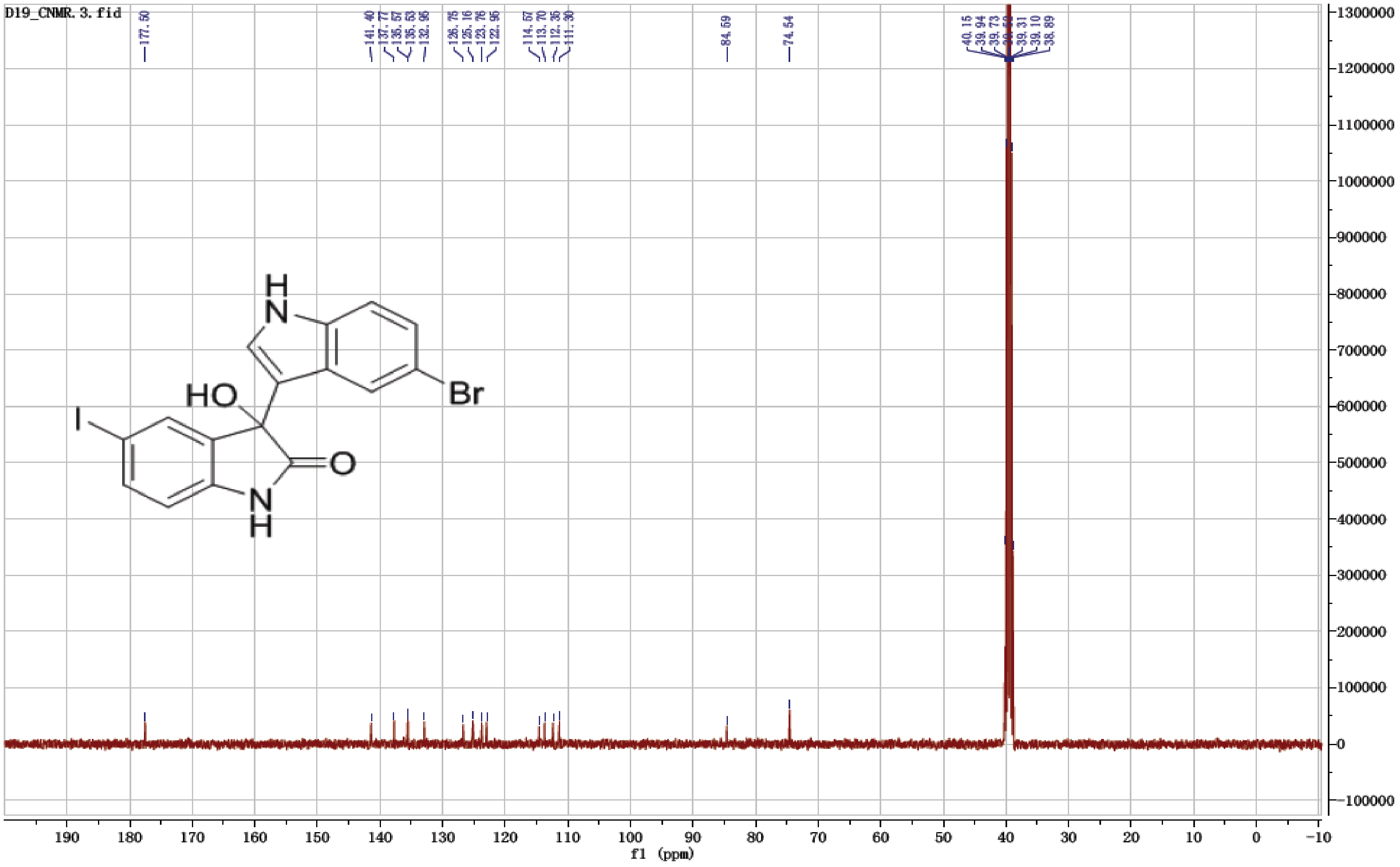
D19_CNMR.3.fid
177.50
141.40
137.77
135.57
135.53
132.95
126.75
125.16
123.76
122.95
114.57
113.70
112.35
111.30
84.59
74.54
40.15
39.94
39.73
39.10
38.89
39.31
HO
I
N
H
O
Br
f1 (ppm)

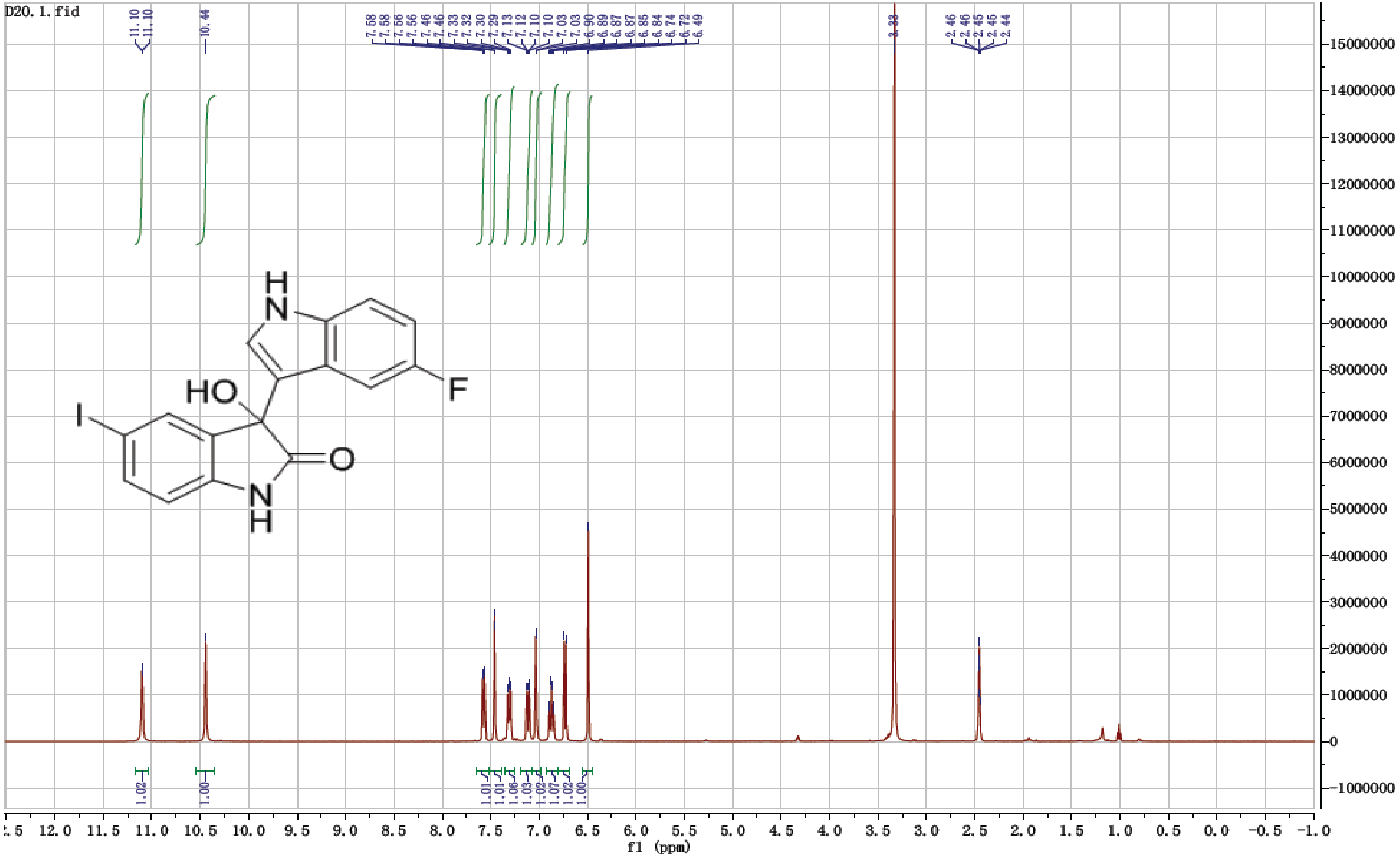

D20.1.fid
11.10
11.10
10.44
7.58
7.58
7.56
7.56
7.46
7.46
7.33
7.32
7.30
7.29
7.13
7.12
7.10
7.10
7.03
7.03
6.90
6.89
6.87
6.85
6.84
6.74
6.72
6.49
3.33
2.46
2.46
2.45
2.45
2.44
HO
I
F
O
N
H
1.02
1.00
1.01
1.01
1.06
1.03
1.02
1.07
1.02
1.00
f1 (ppm)

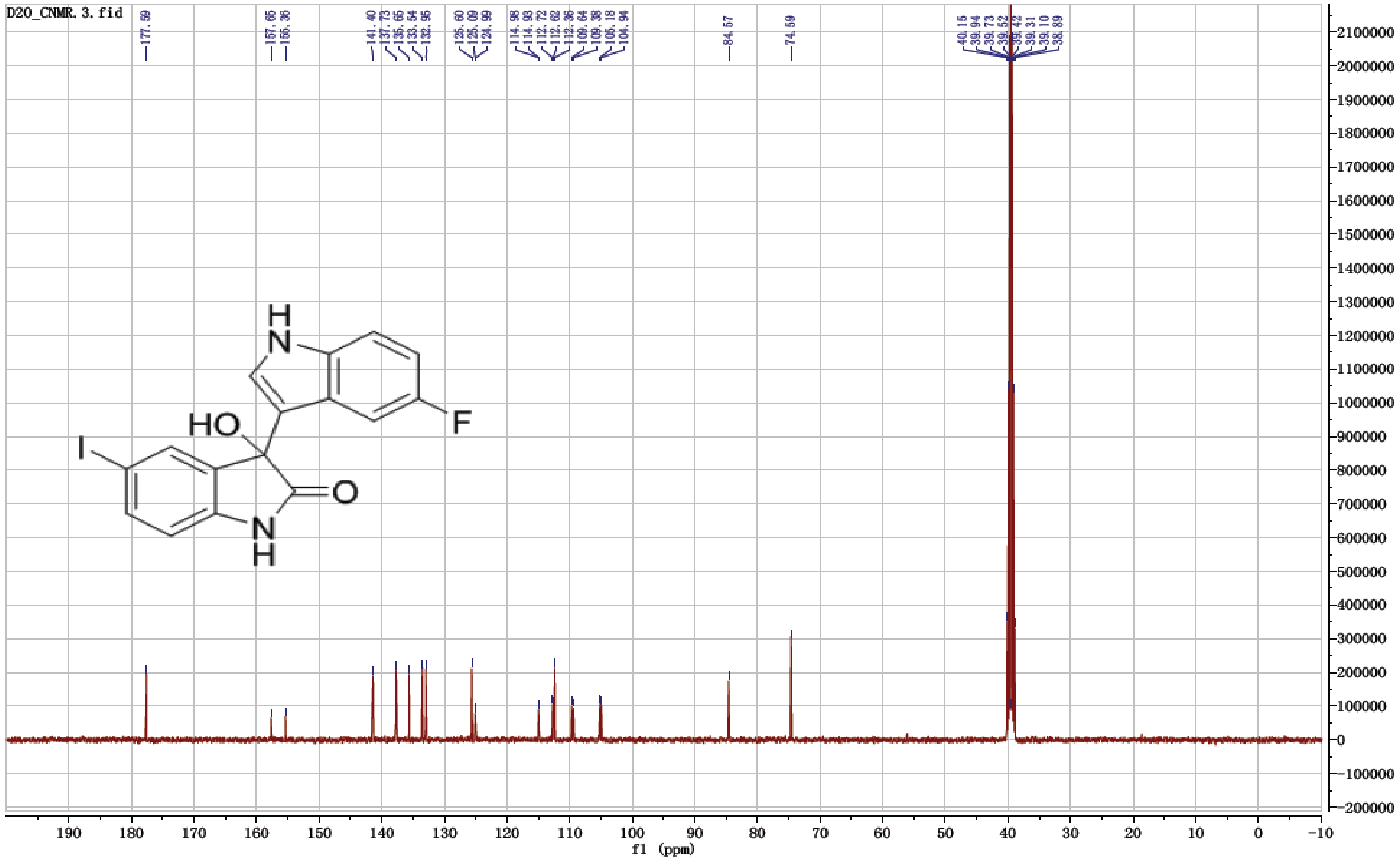
D20_CNMR.3.fid
177.59
157.65
155.36
141.40
137.73
135.65
133.54
132.95
125.60
125.09
124.99
114.98
114.93
112.72
112.62
112.36
109.64
109.38
105.18
104.94
84.57
74.59
40.15
39.94
39.73
39.52
39.42
39.31
39.10
38.89
HO
I
F
O
N
H
f1 (ppm)

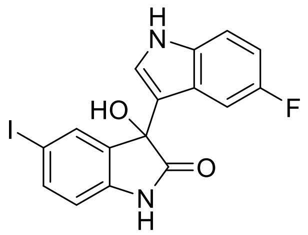

H
N
HO
I
F
O
N
H

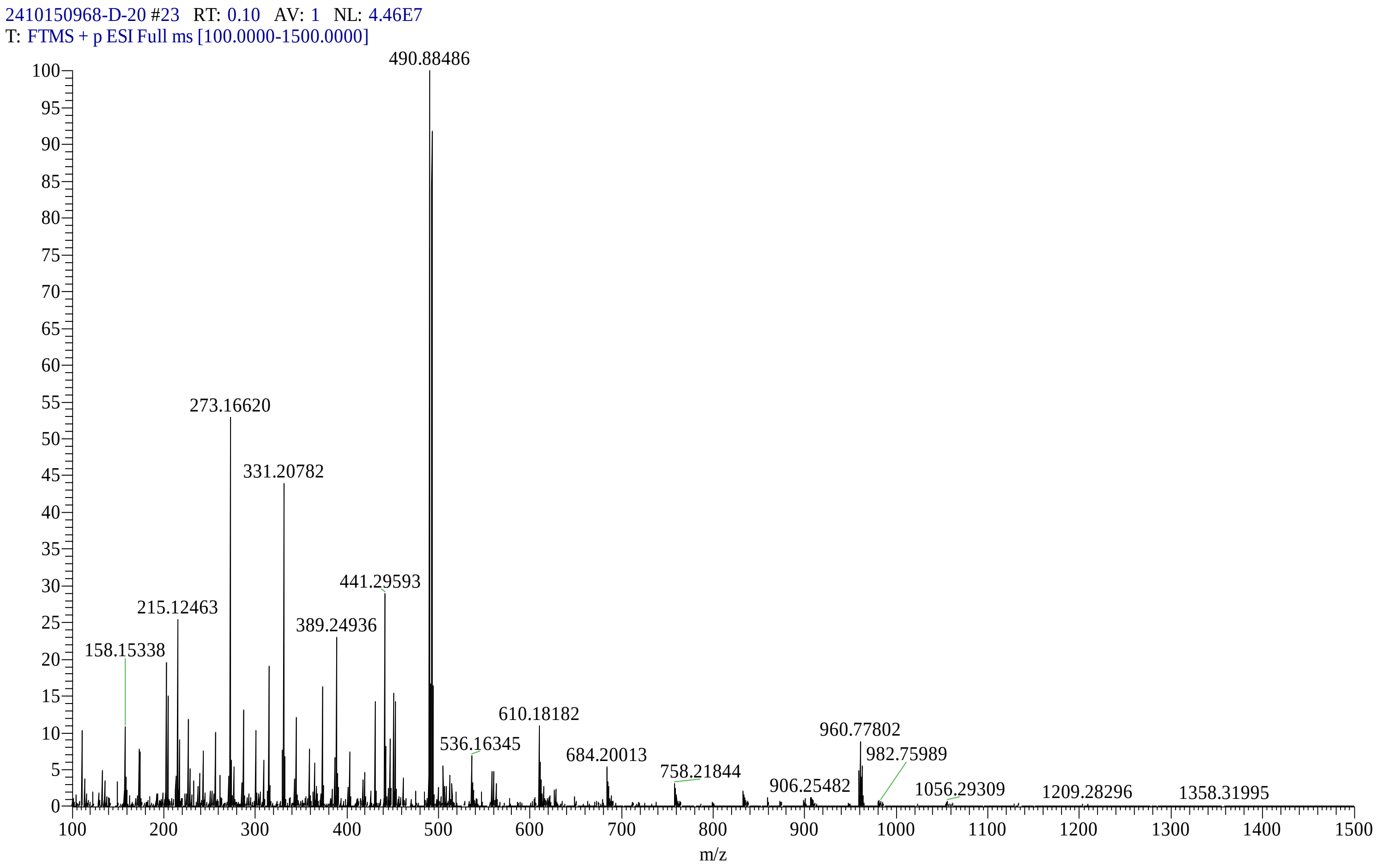

2410150968-D-20 #23 RT: 0.10 AV: 1 NL: 4.46E7
T: FTMS + p ESI Full ms [100.0000-1500.0000]
490.88486
273.16620
331.20782
441.29593
215.12463
389.24936
158.15338
610.18182
536.16345
684.20013
758.21844
906.25482
960.77802
982.75989
1056.29309
1209.28296
1358.31995
m/z

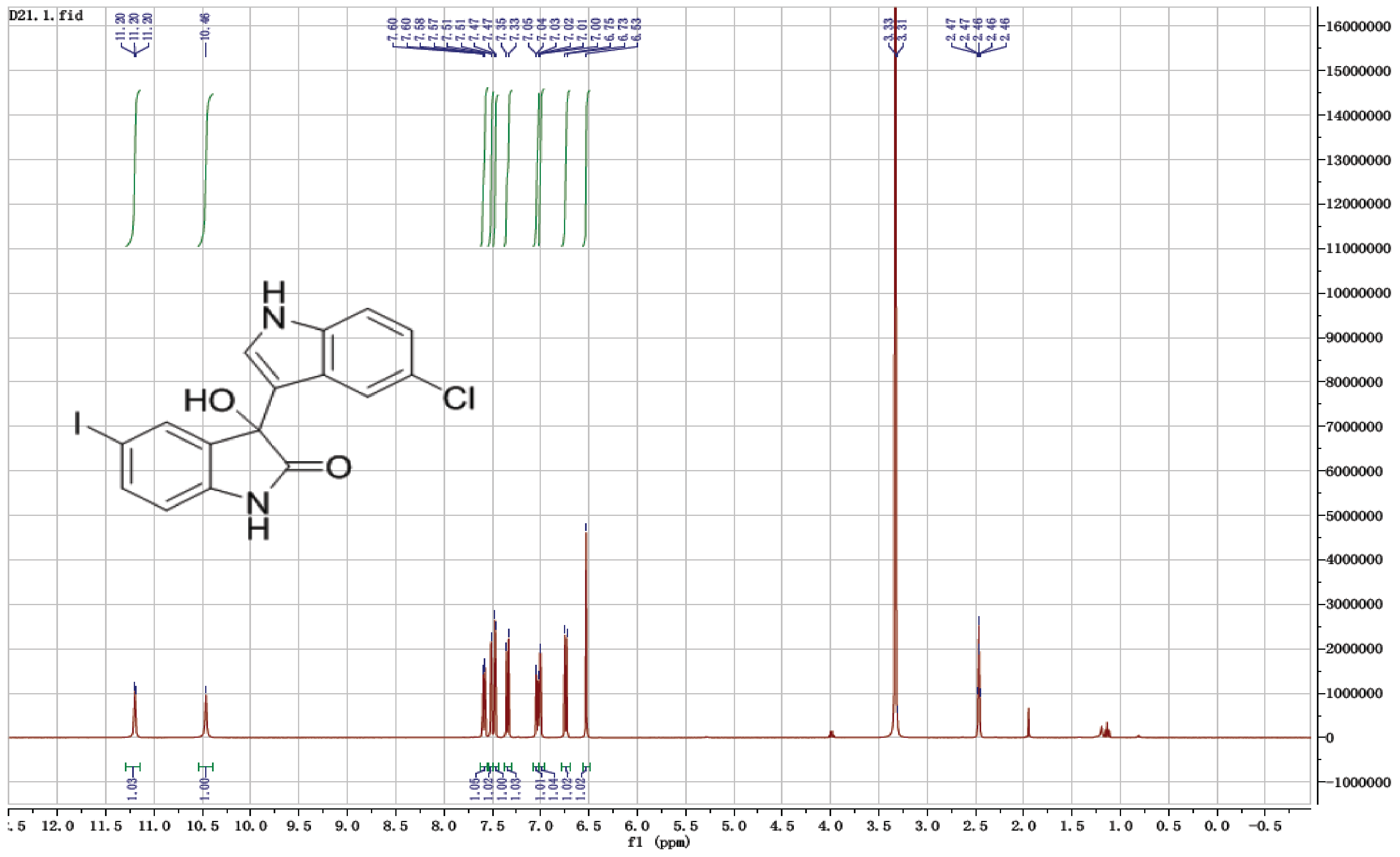

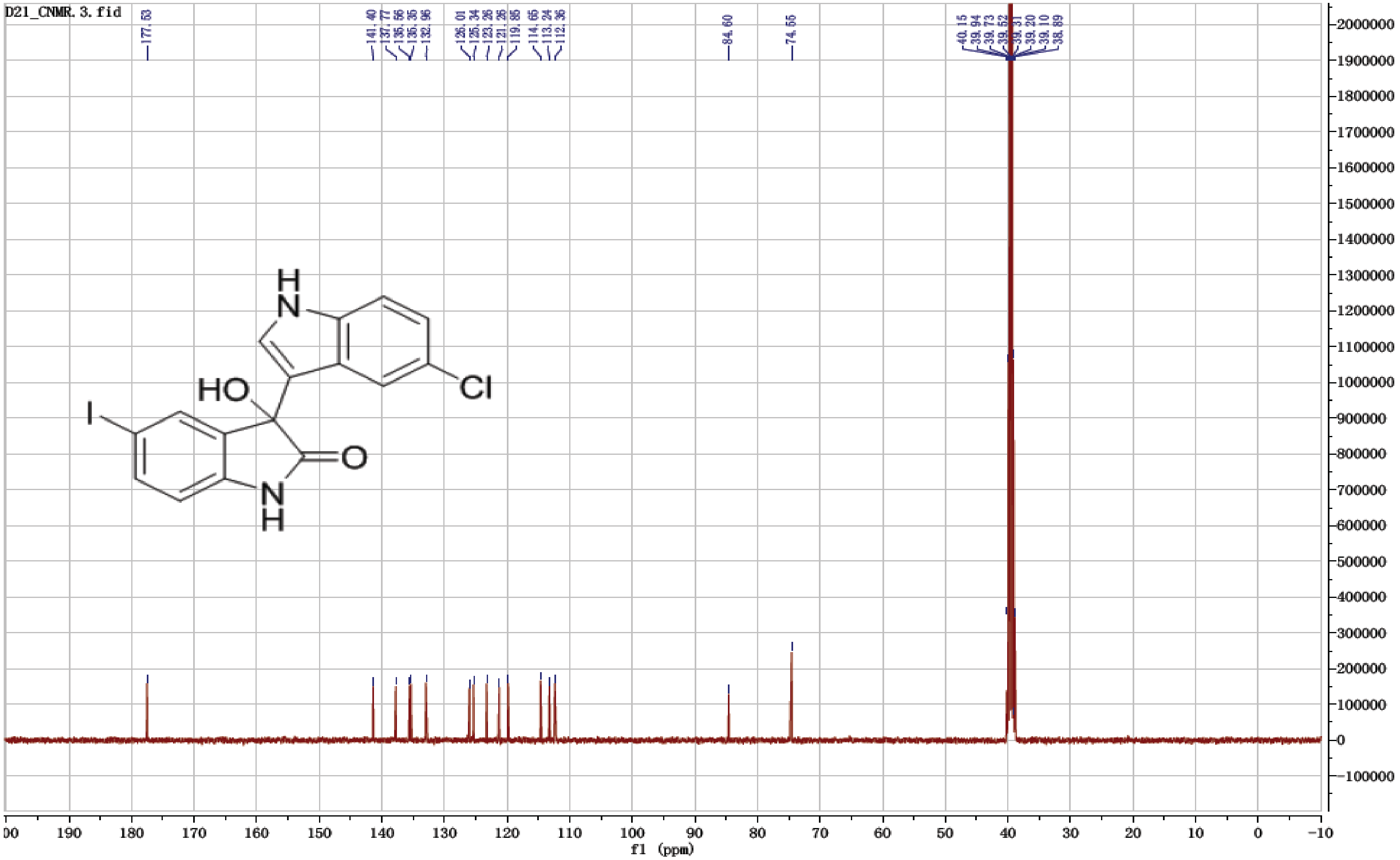
D21_CNMR.3.fid
177.53
141.40
137.77
135.56
135.35
132.96
126.01
125.34
123.26
121.35
119.85
114.65
113.24
112.36
84.60
74.55
40.15
39.94
39.73
39.52
39.31
39.20
39.10
38.89
f1 (ppm)
HO
Cl
I
O
N
H

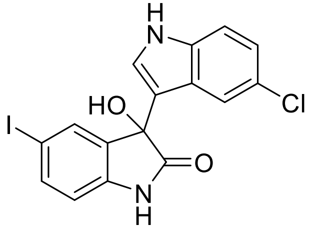

H
N
Cl
HO
I
O
N
H

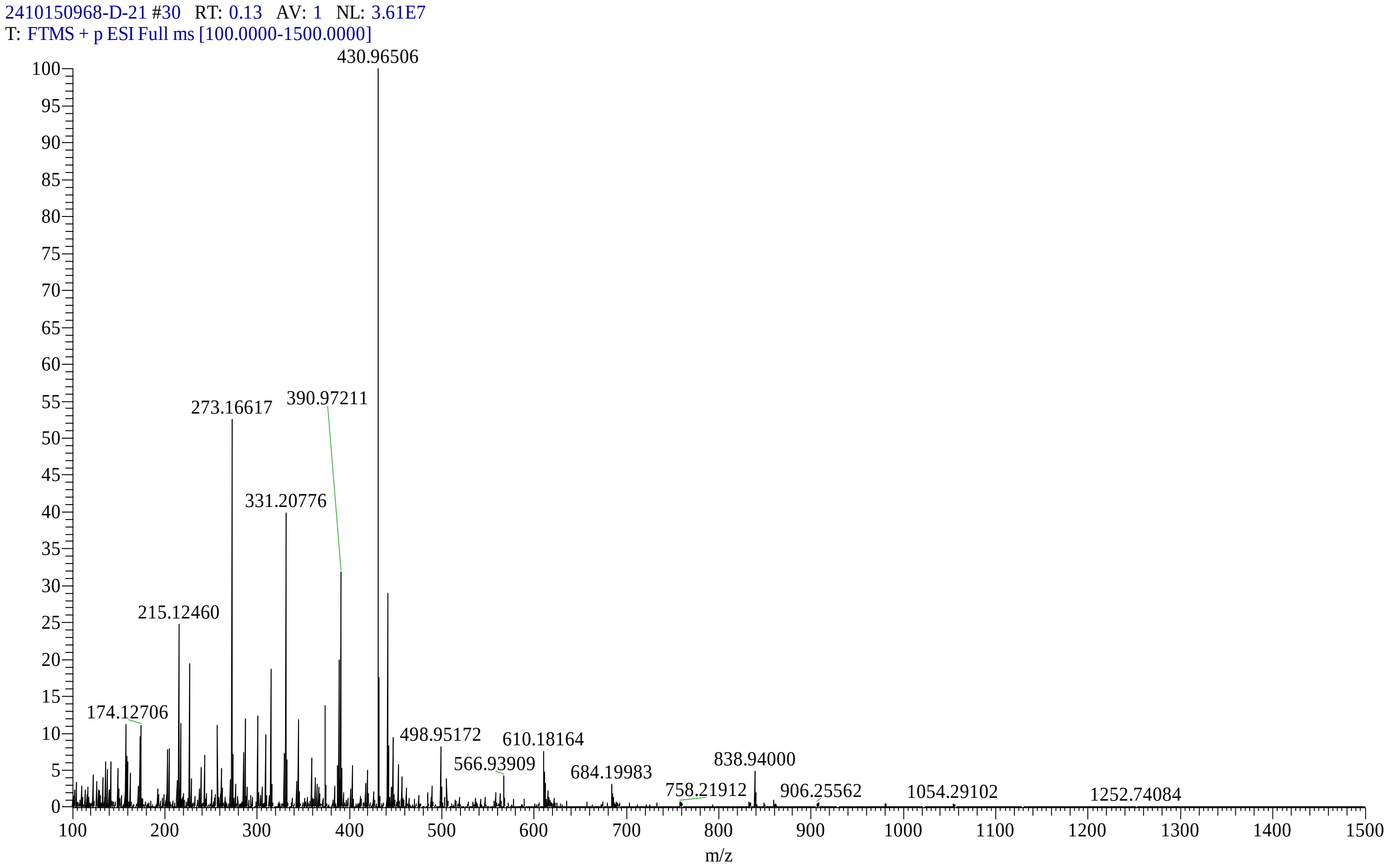

2410150968-D-21 #30 RT: 0.13 AV: 1 NL: 3.61E7
T: FTMS + p ESI Full ms [100.0000-1500.0000]
430.96506
390.97211
273.16617
331.20776
215.12460
174.12706
498.95172
610.18164
566.93909
684.19983
838.94000
758.21912
906.25562
1054.29102
1252.74084
m/z

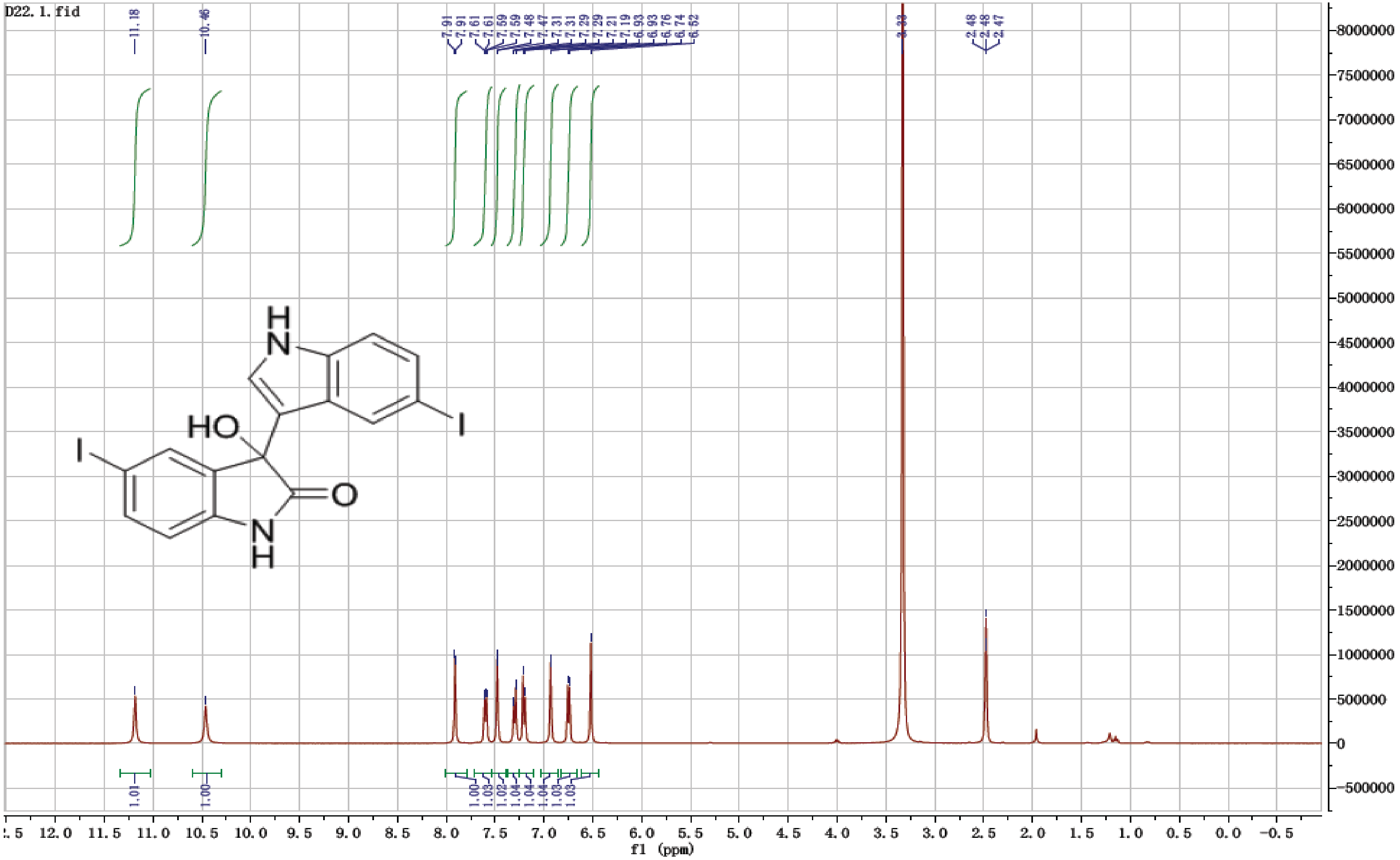
D22.1.fid
11.18
10.46
7.91
7.91
7.61
7.61
7.59
7.59
7.48
7.47
7.31
7.31
7.29
7.29
7.21
7.19
6.93
6.93
6.76
6.74
6.52
3.33
2.48
2.48
2.47
1.01
1.00
1.00
1.03
1.02
1.04
1.04
1.04
1.03
1.03
f1 (ppm)
HO
I
O
N
H

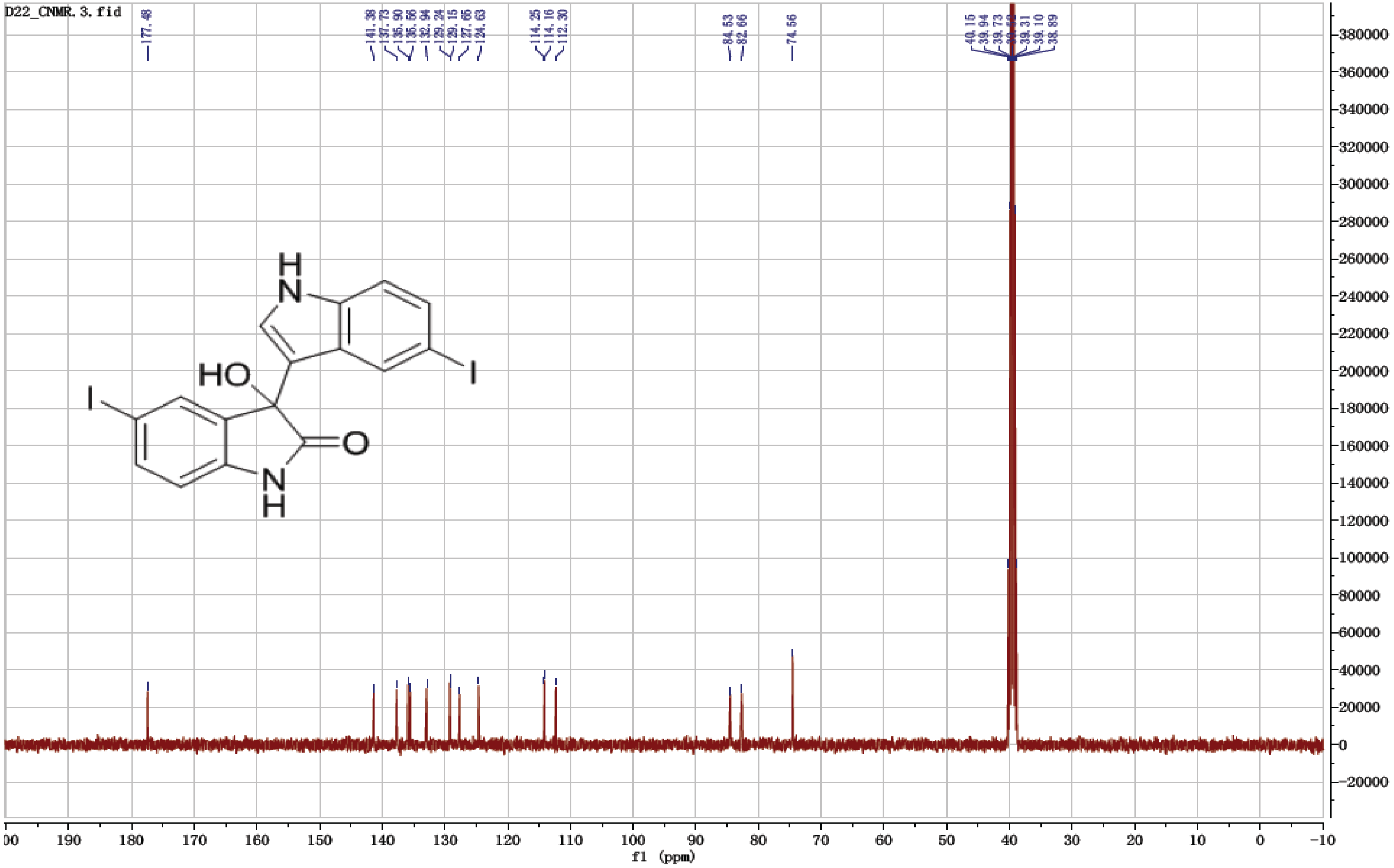
D22_CNMR.3.fid
177.48
141.38
137.73
135.90
135.56
132.94
129.24
129.15
127.65
124.63
114.25
114.16
112.30
84.53
82.66
74.56
40.15
39.94
39.73
39.31
39.10
38.89
HO
O
I
N
H
f1 (ppm)

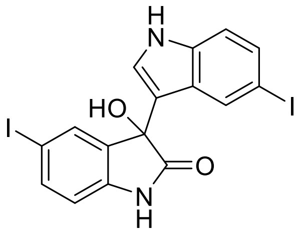

H
N
HO
I
I
O
N
H

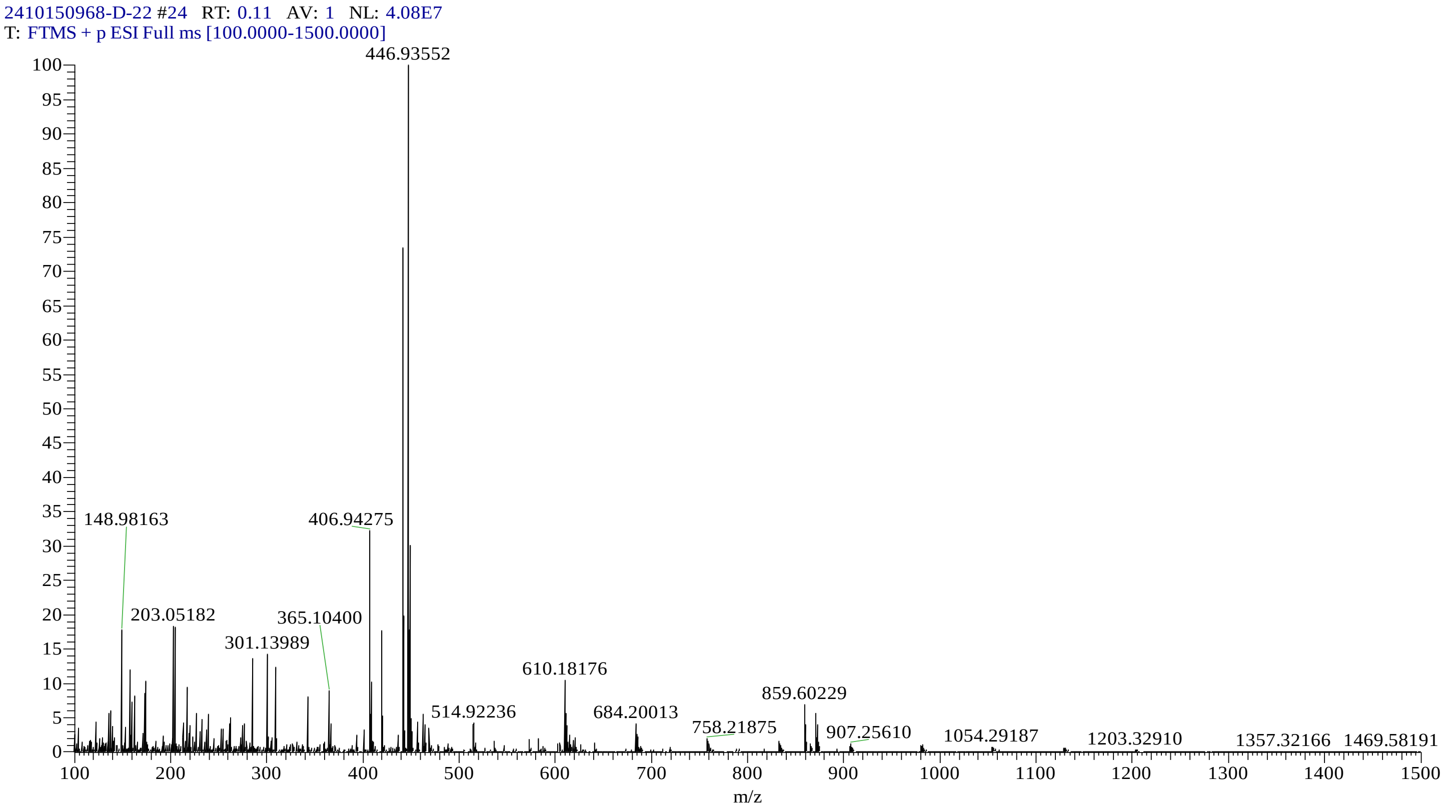

2410150968-D-22 #24 RT: 0.11 AV: 1 NL: 4.08E7
T: FTMS + p ESI Full ms [100.0000-1500.0000]
446.93552
148.98163
406.94275
203.05182
365.10400
301.13989
610.18176
514.92236
684.20013
859.60229
758.21875
907.25610
1054.29187
1203.32910
1357.32166
1469.58191
m/z

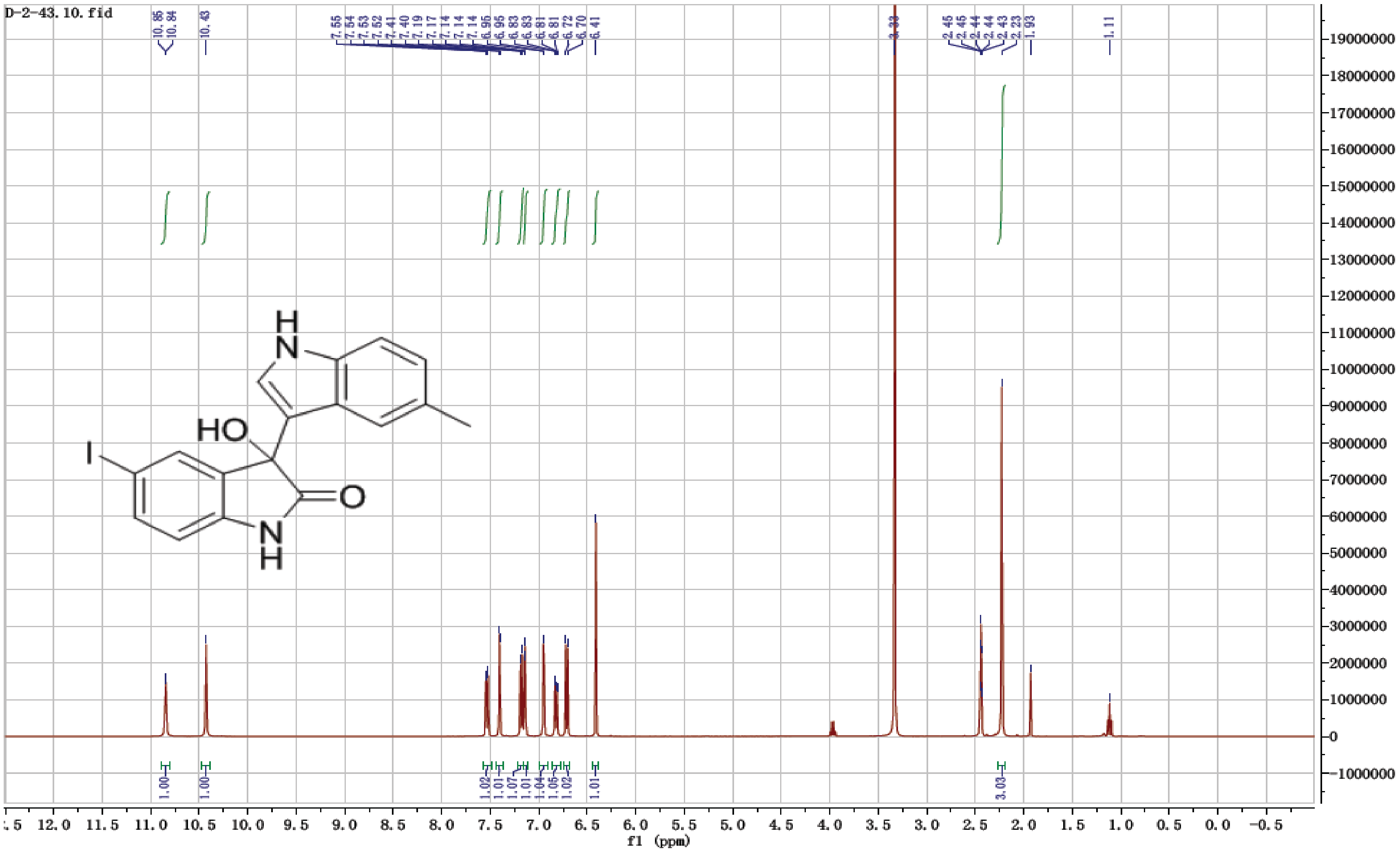

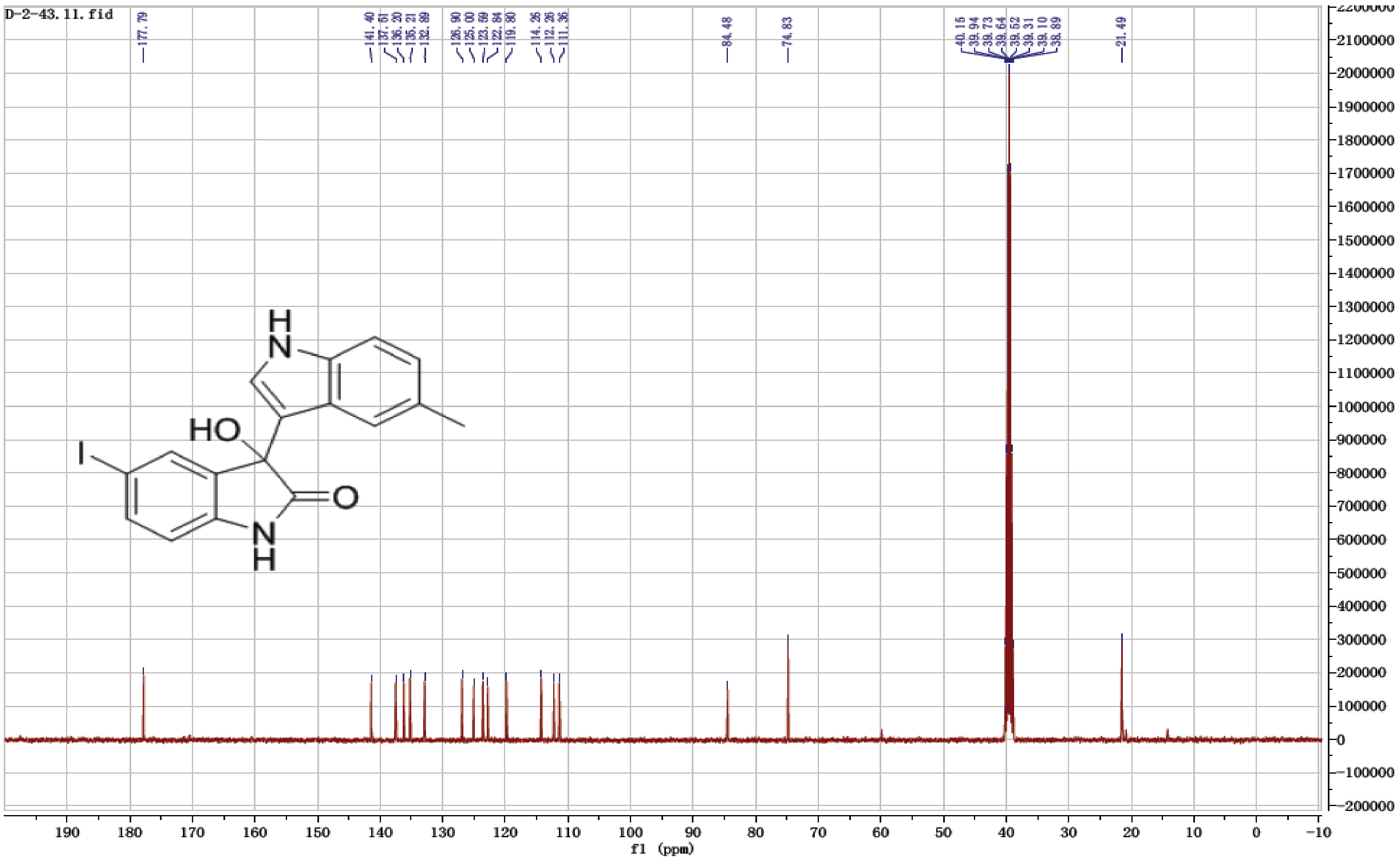
D-2-43.11.fid
177.79
141.40
137.51
136.20
135.21
132.89
126.90
125.00
123.59
122.84
119.80
114.26
112.26
111.36
84.48
74.83
40.15
39.94
39.73
39.64
39.52
39.31
39.10
38.89
21.49
HO
I
O
N
H
f1 (ppm)

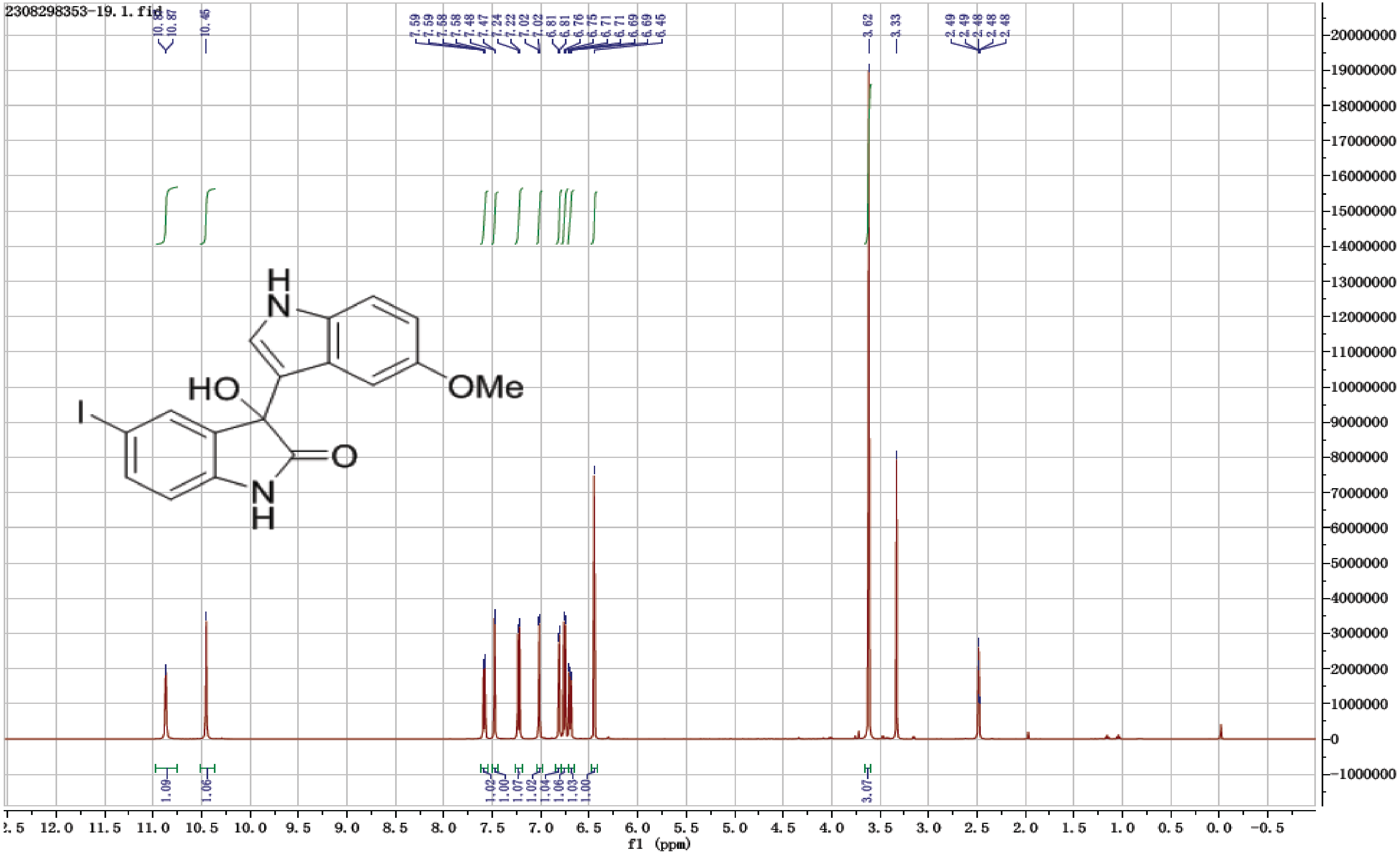

2308298353-19.1.fid
10.88
10.87
10.45
7.59
7.59
7.58
7.58
7.48
7.47
7.24
7.22
7.02
7.02
6.81
6.81
6.76
6.75
6.71
6.71
6.69
6.45
3.62
3.33
2.49
2.49
2.48
2.48
2.48
HO
I
N
H
O
H
N
OMe
1.09
1.06
1.02
1.00
1.07
1.02
1.04
1.06
1.03
1.00
3.07
f1 (ppm)

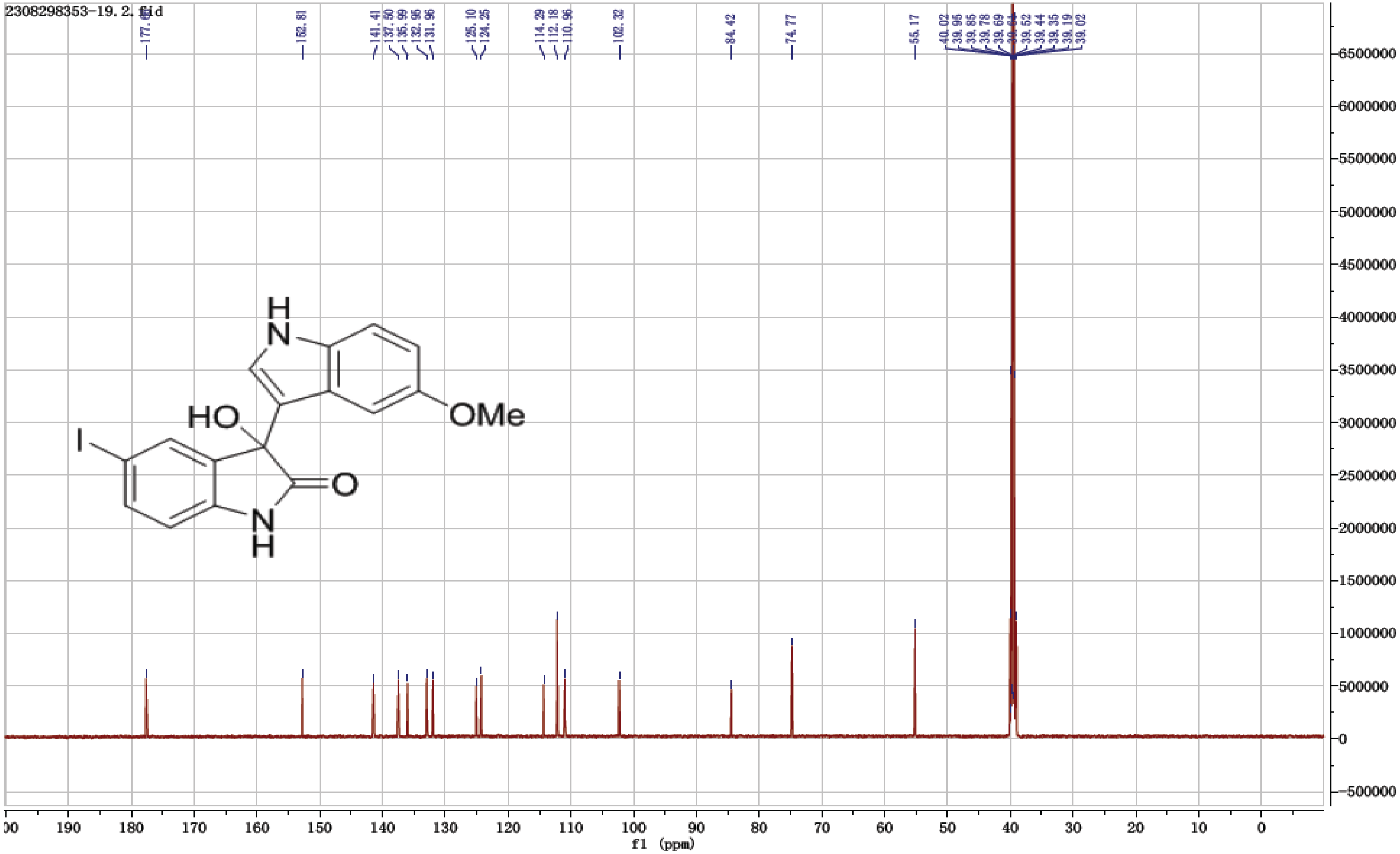
2308298353-19.2.fid
177.69
152.81
141.41
137.50
135.99
132.96
131.96
125.10
124.25
114.29
112.18
110.96
102.32
84.42
74.77
55.17
40.02
39.95
39.85
39.78
39.69
39.52
39.44
39.35
39.19
39.02
HO
I
N
H
O
N
H
OMe
6500000
6000000
5500000
5000000
4500000
4000000
3500000
3000000
2500000
2000000
1500000
1000000
500000
0
-500000
190
180
170
160
150
140
130
120
110
100
90
80
70
60
50
40
30
20
10
0
f1 (ppm)
